# An anisotropic van der Waals dielectric for symmetry engineering in functionalized heterointerfaces

**DOI:** 10.1038/s41467-023-41295-6

**Published:** 2023-09-09

**Authors:** Zeya Li, Junwei Huang, Ling Zhou, Zian Xu, Feng Qin, Peng Chen, Xiaojun Sun, Gan Liu, Chengqi Sui, Caiyu Qiu, Yangfan Lu, Huiyang Gou, Xiaoxiang Xi, Toshiya Ideue, Peizhe Tang, Yoshihiro Iwasa, Hongtao Yuan

**Affiliations:** 1 National Laboratory of Solid State Microstructures, and Collaborative Innovation Center of Advanced Microstructures, Nanjing University, Nanjing, 210093 China; 2 College of Engineering and Applied Sciences, and Jiangsu Key Laboratory of Artificial Functional Materials, Nanjing University, Nanjing, 210023 China; 3 School of Materials Science and Engineering, Beihang University, Beijing, 100191 China; 4 School of Physics, Nanjing University, Nanjing, 210093 China; 5 College of Materials Sciences and Engineering, National Engineering Research Center for Magnesium Alloys, Chongqing University, Chongqing, 400030 China; 6 Center for High Pressure Science and Technology Advanced Research, Beijing, 100094 China; 7 Quantum Phase Electronic Center and Department of Applied Physics, The University of Tokyo, Tokyo, 113-8656 Japan; 8 Institute for Solid State Physics, The University of Tokyo, Chiba, 277-8581 Japan; 9 Max Planck Institute for the Structure and Dynamics of Matter, Center for Free Electron Laser Science, Hamburg, 22761 Germany; 10 RIKEN Center for Emergent Matter Science, Hirosawa 2-1, Wako, 351-0198 Japan

**Keywords:** Electronic devices, Surfaces, interfaces and thin films, Two-dimensional materials, Electronic properties and materials, Electronic and spintronic devices

## Abstract

Van der Waals dielectrics are fundamental materials for condensed matter physics and advanced electronic applications. Most dielectrics host isotropic structures in crystalline or amorphous forms, and only a few studies have considered the role of anisotropic crystal symmetry in dielectrics as a delicate way to tune electronic properties of channel materials. Here, we demonstrate a layered anisotropic dielectric, SiP_2_, with non-symmorphic twofold-rotational *C*_2_ symmetry as a gate medium which can break the original threefold-rotational *C*_3_ symmetry of MoS_2_ to achieve unexpected linearly-polarized photoluminescence and anisotropic second harmonic generation at SiP_2_/MoS_2_ interfaces. In contrast to the isotropic behavior of pristine MoS_2_, a large conductance anisotropy with an anisotropy index up to 1000 can be achieved and modulated in SiP_2_-gated MoS_2_ transistors. Theoretical calculations reveal that the anisotropic moiré potential at such interfaces is responsible for the giant anisotropic conductance and optical response. Our results provide a strategy for generating exotic functionalities at dielectric/semiconductor interfaces via symmetry engineering.

## Abstract (web summary)

Here, the authors demonstrate that a layered anisotropic dielectric material, SiP_2_, can break the rotational symmetry of 2D MoS_2_, leading to linearly polarized photoluminescence emission and conductance anisotropy ratios up to 1000 in gated SiP_2_/MoS_2_ heterostructures.

## Introduction

Symmetry breaking in low dimensional heterostructures can provide unprecedented possibilities to generate emergent quantum phenomena in condensed matter physics^1–8^. In general, van der Waals (vdW) dielectric at atomically-sharp semiconductor/dielectric interfaces can break the symmetry of the target materials and form moiré patterns with specific lattice mismatch^2,9,10^, exhibiting remarkable capabilities to control electronic states and further realize exotic quantum phenomena therein. Examples of these interfacial phenomena such as Chern insulating states^11,12^, charge density wave states^13,14^ and topological valley currents^15^ have been demonstrated in the heterointerfaces based on *h-*BN dielectric^2,9,11–17^, in which both *h*-BN dielectric and channel materials (graphene or transition metal dichalcogenides, TMDCs) show threefold-rotational symmetry (*C*_3_) along the out-of-plane axis at their interface. In contrast, a low-symmetric dielectric material without *C*_3_ symmetry (for example, with *C*_2_ symmetry) can in principle break the *C*_3_ symmetry in monolayer semiconductors by forming anisotropic moiré potentials at the interface^5^ and result in exotic optical response and anisotropic electronic transport, while retaining the gating capability as a dielectric medium. Therefore, the vdW dielectrics with lower lattice symmetry can generate unique moiré physics and additional device functionalities at the symmetry-mismatched interfaces. However, an experimental confirmation of such a strategy remains elusive.

Herein, we demonstrate a unique anisotropic layered dielectric material SiP_2_ and reveal its capability to generate giant anisotropy in optical response and electronic transport in isotropic TMDC semiconductors via symmetry engineering. We realize a high-performance SiP_2_-gating MoS_2_ transistor with large on/off ratios >10^5^ and low leakage currents (far below the low power limit) and further observe an insulator-to-metal transition in SiP_2_-gated 1L-MoS_2_, indicating a great dielectric capability of SiP_2_ material. Surprisingly, a linearly-polarized photoluminescence and an anisotropic second harmonic generation signals are observed in 1L-MoS_2_/SiP_2_ heterostructure, which are in sharp contrast to the isotropic features of pristine 1L-MoS_2_. Remarkably, we find a large anisotropic conductance in the 1L-MoS_2_/SiP_2_ heterostructure and the tunable anisotropy index reaches a considerable value of 1000 with SiP_2_ gating, which is among the largest values reported so far, including intrinsically anisotropic materials^18^. Our first-principles calculations reveal that such giant anisotropy in optical response and electronic transport result from the generated anisotropic moiré potential in 1L-MoS_2_/SiP_2_ heterostructure that strongly renormalizes the structural and electronic properties of 1L-MoS_2_ at the heterointerface. Note that the interfacial symmetry engineering by breaking the *C*_3_ symmetry of 1L-MoS_2_ using SiP_2_ dielectric with *C*_2_ symmetry can be regarded as a strategy to tune electronic properties of channel semiconductors and realize moiré physics at the heterointerfaces.

## Results

### MoS_2_ transistors gated with SiP_2_ dielectric

**Fig. 1:**
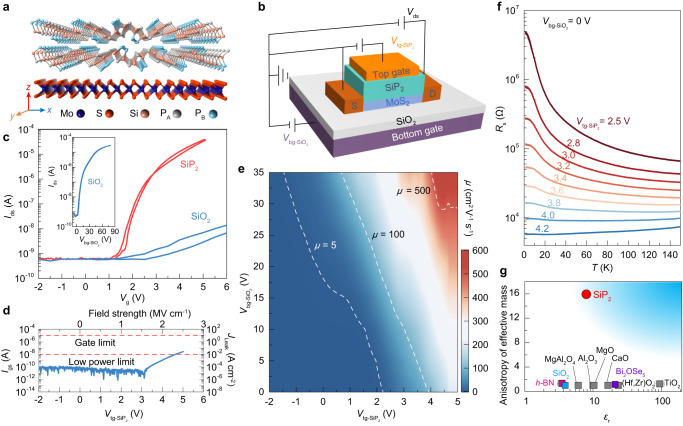
High-performance MoS_2_ transistors gated with a SiP_2_ dielectric with non-symmorphic *C*_2_ rotational symmetry. **a** Schematic structure of the cross-section of a MoS_2_/SiP_2_ heterostructure. Orange, gray, light blue, red, and dark blue spheres represent Si, P_A_, P_B_, S, and Mo atoms, respectively. The P_B_ atoms form the unique quasi-1D P_B_–P_B_ chains along the *y* direction in SiP_2_ crystal lattice. **b** Schematic illustration of MoS_2_/SiP_2_ dual-gated device. The top gate medium is 20-nm-thick SiP_2_, and the bottom gate medium is 300-nm-thick SiO_2_. D and S represent the drain and source electrodes. ${V}_{{{{{{\rm{tg}}}}}}{-{{{{{\rm{SiP}}}}}}}_{2}}$ and ${V}_{{{{{{\rm{bg}}}}}}{-{{{{{\rm{SiO}}}}}}}_{2}}$ are the top gate voltage and back gate voltage, respectively. And ${V}_{{{{{{\rm{ds}}}}}}}$ is the source-drain voltage applied on the MoS_2_ channel material. **c** Transfer curves of the 5-nm-MoS_2_-based transistor at 2 K when sweeping top (red curve) and bottom (blue curve) gate voltages via the SiP_2_ and SiO_2_ dielectric media. The inset is a transfer curve with scanning ${V}_{{{{{{\rm{bg}}}}}}{-{{{{{\rm{SiO}}}}}}}_{2}}$. **d** Leakage current ${I}_{{{{{{\rm{gs}}}}}}}$ as a function of ${V}_{{{{{{\rm{t}}}}}}{{{{{\rm{g}}}}}}{-{{{{{\rm{SiP}}}}}}}_{2}}$ on a 5-nm MoS_2_ device at 2 K. ${I}_{{{{{{\rm{gs}}}}}}}$ and ${V}_{{{{{{\rm{t}}}}}}{{{{{\rm{g}}}}}}{-{{{{{\rm{SiP}}}}}}}_{2}}$ are rescaled to the leakage current density (${J}_{{{{{{\rm{Leak}}}}}}}$) and the electric field strength for a better comparison (right and top axes). Horizontal red lines mark the limits of leakage current density for various types of integrated circuits. **e** The field-effect mobility $\mu$ as a function of ${V}_{{{{{{\rm{t}}}}}}{{{{{\rm{g}}}}}}{-{{{{{\rm{SiP}}}}}}}_{2}}$ and ${V}_{{{{{{\rm{bg}}}}}}{-{{{{{\rm{SiO}}}}}}}_{2}}$ for the 5-nm-MoS_2_/SiP_2_ device. The white dashed lines highlight that $\mu$ equals 5, 100, and 500 cm^2^ V^–1^ s^–1^. **f** Sheet resistances (${R}_{{{{{{\rm{s}}}}}}}$) versus temperature of a 1L-MoS_2_ device under different ${V}_{{{{{{\rm{tg}}}}}}{-{{{{{\rm{SiP}}}}}}}_{2}}$ values. **g** Comparison of the anisotropy ratio of the effective mass for dielectrics with different relative dielectric constants (${\varepsilon }_{{{{{{\rm{r}}}}}}}$). The anisotropy ratio is defined as the ratio of the electron effective mass (*m*_e_) along the *y* and *x* directions of the corresponding lattice.

To evaluate the performance of the SiP_2_ dielectric, we measured the transfer characteristics of MoS_2_ transistors with dual-gate geometry in which 20-nm-thick SiP_2_ and 300-nm-thick SiO_2_ are used as top and bottom gate media (Fig. 1a, b and Supplementary Fig. 1). As shown in Fig. 1c, when sweeping the top gate voltage ${V}_{{{{{{\rm{t}}}}}}{{{{{\rm{g}}}}}}{-{{{{{\rm{SiP}}}}}}}_{2}}$ up to 5 V, the 5-nm-thick MoS_2_ transistor shows an on/off ratio as high as 10^5^, which is comparable to those values in *h-*BN-gated MoS_2_ transistors (Supplementary Table 2) and meets the well-known criterion for practical logic circuit applications^19^. In contrast, when sweeping the bottom gate voltage ${V}_{{{{{{\rm{bg}}}}}}{-{{{{{\rm{SiO}}}}}}}_{2}}$ to ~5 V, the transistor generates an on/off ratio as small as 10, and requires a large ${V}_{{{{{{\rm{bg}}}}}}{-{{{{{\rm{SiO}}}}}}}_{2}}$ over 80 V to achieve an on/off ratio of 10^5^ (inset of Fig. 1c). This comparison directly demonstrates that, compared to SiO_2_, the SiP_2_ gate dielectric with larger dielectric constant and smaller thickness can achieve great capacitive capability. The measured leakage current of SiP_2_-gated MoS_2_ transistor is as small as approximately 10^–5^ A cm^–2^ at an external electric field strength of 1.5 MV cm^–1^ (Fig. 1d). Such a low leakage current is comparable to those of transistors gated by high-*κ* dielectrics^19–21^ such as Al_2_O_3_, HfO_2_ or Bi_2_SeO_5_, and better than the criteria of the low-power limit and the standard complementary metal–oxide–semiconductor gate limit^19^. With increasing ${V}_{{{{{{\rm{t}}}}}}{{{{{\rm{g}}}}}}{-{{{{{\rm{SiP}}}}}}}_{2}}$, the field-effect mobility $\mu$ at 2 K can reach ~600 cm^2^ V^–1^ s^–1^ when ${V}_{{{{{{\rm{bg}}}}}}{-{{{{{\rm{SiO}}}}}}}_{2}}$ is fixed at 35 V (Fig. 1e). Even for SiP_2_-gated 1L-MoS_2_ transistors with the same device geometry (Supplementary Fig. 2), the mobility of 330 cm^2^ V^–1^ s^–1^ at 2 K is better than those reported values in HfO_2_-gated 1L-MoS_2_ devices^22^ (174 cm^2^ V^–1^ s^–1^ at 4 K), indicating that the vdW SiP_2_ material with a large dielectric constant can effectively reduce the charge scattering and increase the mobility of MoS_2_ transistors. Such excellent performance with high on/off ratio, low leakage current, and high mobility in MoS_2_/SiP_2_ devices suggests that layered SiP_2_ can be a high-performance dielectric in switching devices.

To demonstrate the great gate tunability of SiP_2_ dielectric, we measured the temperature-dependent sheet resistance (${R}_{{{{{{\rm{s}}}}}}}$–$T$) and observed a gating-induced insulator–metal transition in the 1L-MoS_2_/SiP_2_ transistor. As shown in Fig. 1f, the ${R}_{{{{{{\rm{s}}}}}}}$–$T$ curves show typical insulating behavior with negative temperature coefficients ${{{{{\rm{d}}}}}}{R}_{{{{{{\rm{s}}}}}}}/{{{{{\rm{d}}}}}}T$ and follow a thermal activation dependence^23^ when ${V}_{{{{{{\rm{tg}}}}}}{-{{{{{\rm{SiP}}}}}}}_{2}}$ < 3.8 V and ${V}_{{{{{{\rm{bg}}}}}}{-{{{{{\rm{SiO}}}}}}}_{2}}$ = 0 V (Supplementary Fig. 3a). The extracted activation energy decreases monotonically from ~7 meV to near zero as ${V}_{{{{{{\rm{tg}}}}}}{-{{{{{\rm{SiP}}}}}}}_{2}}$ increases from 2.5 V to 3.8 V (Supplementary Fig. 3b). As a result, ${R}_{{{{{{\rm{s}}}}}}}$ starts to decrease with cooling temperature and the positive ${{{{{\rm{d}}}}}}{R}_{{{{{{\rm{s}}}}}}}/{{{{{\rm{d}}}}}}T$ shows the typical metallic behavior when ${V}_{{{{{{\rm{tg}}}}}}{-{{{{{\rm{SiP}}}}}}}_{2}}$ $>$ 3.8 V, directly indicating an insulator–metal transition^23^ in SiP_2_-gated 1L-MoS_2_. Such a transition in SiP_2_-gated 1L-MoS_2_ transistor directly manifests the excellent dielectric property of layered SiP_2_ as a gate medium to modulate the electronic states of ultrathin semiconductors.

To experimentally evaluate the dielectric constant of SiP_2_, we measured the sheet carrier density (${n}_{2{{{{{\rm{D}}}}}}}$) of MoS_2_ (5 nm) as a function of ${V}_{{{{{{\rm{tg}}}}}}{-{{{{{\rm{SiP}}}}}}}_{2}}$ based on Hall effect measurements. The ${n}_{2{{{{{\rm{D}}}}}}}$ values of top-gated MoS_2_ remain nearly unchanged below the threshold voltage of 1.7 V and can be continually modulated to 8 × 10^12 ^cm^–2^ by increasing ${V}_{{{{{{\rm{tg}}}}}}{-{{{{{\rm{SiP}}}}}}}_{2}}$ to 5 V (Supplementary Fig. 4a). Note that the dual-gate-modulated ${n}_{2{{{{{\rm{D}}}}}}}$ can reach a maximum value close to 10^13 ^cm^–2^ (Supplementary Fig. 4b). The relative dielectric constant ${\varepsilon }_{{{{{{\rm{r}}}}}}}$ is evaluated to be 8.1 for SiP_2_ by fitting the linear part of the ${n}_{2{{{{{\rm{D}}}}}}}$–${V}_{{{{{{\rm{tg}}}}}}{-{{{{{\rm{SiP}}}}}}}_{2}}$ data using ${n}_{2{{{{{\rm{D}}}}}}}={\varepsilon }_{0}{\varepsilon }_{{{{{{\rm{r}}}}}}}{V}_{{{{{{\rm{tg}}}}}}{-{{{{{\rm{SiP}}}}}}}_{2}}/(e{t}_{{{{{{{\rm{SiP}}}}}}}_{2}})$, where $e$ is the electron charge, ${\varepsilon }_{0}$ is the vacuum permittivity, and ${t}_{{{{{{{\rm{SiP}}}}}}}_{2}}$ = 20 nm is the thickness of SiP_2_ (more details in “Methods”). Such a dielectric constant of 8.1 in layered SiP_2_ is larger than those of SiO_2_ and *h*-BN dielectrics^24,25^ and comparable to that of Al_2_O_3_ dielectric^24^ (Fig. 1g, a detailed comparison is given in Supplementary Tables 3 and 4), well consistent with the theoretical estimation from first-principles calculations^26^.

**Fig. 2:**
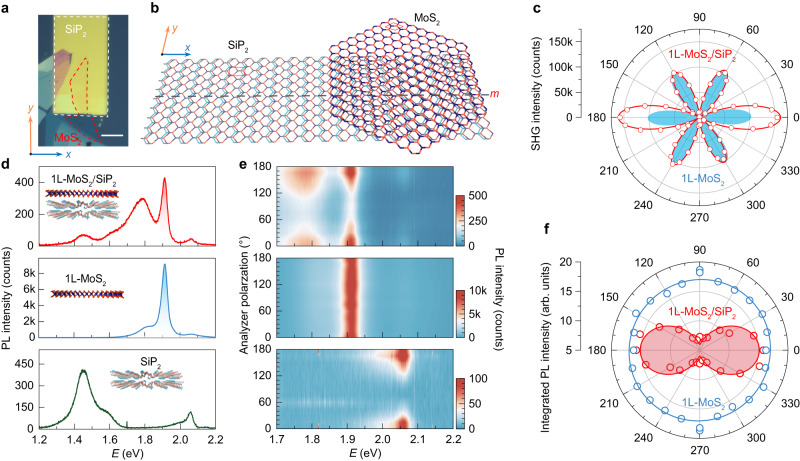
Anisotropic optical response at the TMDC/SiP_2_ interface. **a**, **b** Optical image (**a**) and schematic illustration of the top view (**b**) of a 1L-MoS_2_/SiP_2_ heterostructure, in which the armchair direction of MoS_2_ and the *x* direction of SiP_2_ are parallel. The red and white dashed lines in (**a**) highlight the 1L-MoS_2_ and SiP_2_ sample areas. Scale bar is 20 μm. The red rectangle and diamond in (**b**) represent the unit cells of SiP_2_ and MoS_2_, respectively. The black dashed line represents the mirror plane *m*. **c** Polar plot of polarization-resolved second harmonic generation (SHG) intensities of bare 1L-MoS_2_ (blue shadow) and 1L-MoS_2_/SiP_2_ (red) under the parallel configuration (the detection polarization is parallel to the excitation polarization). The red solid line represents the fitting with Eq. (2) in the “Methods”. **d** Photoluminescence (PL) spectra of SiP_2_ (green curve), 1L-MoS_2_ (blue curve), and 1L-MoS_2_/SiP_2_ heterostructure (red curve) at 77 K. Insets are the corresponding schematics of the measured sample geometry. The exciton emission of 1L-MoS_2_ is highlighted with shadows. **e** Corresponding color plot of the PL intensity in (**d**) as a function of emission photon energy at different detection polarization angles *θ*. Here *θ* denotes the angle between the analyzer polarization direction and the *x* direction, as defined in (**b**). **f** Polar plots of polarization-resolved PL integrated intensities of 1L-MoS_2_ (blue curve) and 1L-MoS_2_/SiP_2_ (red curve and red shaded area). The circles represent the experimental data and the solid curves represent the sine fitting results.

As a typical vdW dielectric with excellent performance, another distinctive nature of SiP_2_ is the anisotropic lattice structure with non-symmorphic *C*_2_ symmetry. In sharp contrast to the highly symmetric crystal structure of those widely used dielectrics (oxides and *h*-BN), such an anisotropic in-plane lattice structure of SiP_2_ leads to a highly anisotropic ratio of electron effective masses (~16, Fig. 1g) for the band edge states in its electronic band structure^26^, and provides an opportunity to engineer the interfacial symmetry of vdW heterostructure combined monolayer TMDCs with SiP_2_. For example, the 1L-MoS_2_/SiP_2_ heterostructure shows no rotational symmetry and can exhibit in-plane anisotropic optical and electronic properties (details discussed below). In particular, if the zigzag direction of MoS_2_ and the P_B_–P_B_ chain of SiP_2_ (the direction parallel to the P_B_–P_B_ chain of SiP_2_ is defined as the *y* direction, while the perpendicular direction is defined as the *x* direction) are aligned in parallel (Fig. 2a, b), the mirror symmetry along the *x* direction can remain in the 1L-MoS_2_/SiP_2_ heterostructure; otherwise, all crystal symmetries in MoS_2_ are broken. The perturbation for the electronic structures of stacked heterostructures can be used to generate in-plane polarization and Berry curvature dipole at the interface^27^, realizing emergent interfacial phenomena such as directional quantum shift current^5^, nonlinear Hall effect^1^ and circular photo-galvanic effect (Supplementary Fig. 5).

### SiP_2_-induced anisotropic optical response in 1L-MoS_2_

To understand the engineered symmetry of heterointerfaces, we performed second harmonic generation (SHG) measurements on 1L-MoS_2_/SiP_2_ heterostructures under a parallel measurement geometry (Fig. 2c). The SHG signal in pristine 1L-MoS_2_ shows a sixfold-rotational symmetric pattern with maxima of SHG intensity along its armchair direction and can be well fitted with Eq. (1), implying the *C*_3_-rotational symmetry of 1L-MoS_2_ samples^28^. While in 1L-MoS_2_/SiP_2_ heterostructure, the SHG signal presents an additional twofold component imposed to the six symmetric petals (detailed analysis in “Methods”). Such a twofold component does not originate from the *C*_3_-symmetric lattice of 1L-MoS_2_ itself, but results from the reduced symmetry at the 1L-MoS_2_/SiP_2_ heterointerface. The SHG intensities can be well fitted with Eq. (2) in the “Methods”, similar to those distorted SHG scenarios in uniaxially-strained 1L-MoS_2_ samples^29,30^. Note that SiP_2_ itself has no SHG signal due to the existing inversion symmetry in its crystal lattice (Supplementary Fig. 9c), ensuring that the observed SHG signal of 1L-MoS_2_/SiP_2_ heterostructure mainly originates from 1L-MoS_2_ whose band structure is effectively modified by the potential on the heterointerface created by the bottom *C*_2_ symmetric SiP_2_. More importantly, the anisotropic SHG response at 1L-MoS_2_/SiP_2_ changes little with increasing SiP_2_ thickness (Supplementary Fig. 11), further confirming that this is an interfacial phenomenon induced by symmetry breaking at the 1L-MoS_2_/SiP_2_ heterointerface between the 1L-MoS_2_ and the topmost SiP_2_ layer.

To investigate the effect of symmetry engineering on the optical properties of such an interface, we performed polarization-dependent photoluminescence (PL) measurements in the 1L-MoS_2_/SiP_2_ heterostructure at 77 K (Fig. 2d, e and Supplementary Fig. 6). For SiP_2_, the PL signal with an excitonic emission energy of 2.06 eV at 77 K shows a linear polarization along the *x* direction of the lattice^26^. In contrast, for 1L-MoS_2_ without SiP_2_ stacking layer, the excitonic emission of 1L-MoS_2_ at 1.91 eV at 77 K remains unchanged with the detection polarization angle and shows no linear polarization. While, for the 1L-MoS_2_/SiP_2_ heterostructure, the excitonic state of 1L-MoS_2_ with an emission energy of 1.91 eV becomes linearly polarized along the *x* direction of SiP_2_ (Fig. 2f and Supplementary Fig. 7). The similar results can be observed at 77 K and 300 K in 1L-WS_2_/SiP_2_ heterostructure (Supplementary Fig. 8), indicating the anisotropic PL response in TMDC/SiP_2_ is robust with temperatures. Similar anisotropic SHG responses are also observed in 1L-WS_2_/SiP_2_ heterostructure (details in Supplementary Fig. 9), indicating that SiP_2_ dielectric can effectively engineer the symmetry of its neighboring monolayer TMDC through tunable interlayer interactions. More interestingly, the anisotropic PL and SHG responses strongly depended on the twist angle between MoS_2_ and SiP_2_, and the corresponding anisotropy dramatically decreased when the mirror symmetry of moiré superlattice varnishes with changing the twist angle (Supplementary Figs. 12 and 13). This result indicates that the mirror symmetry of MoS_2_/SiP_2_ moiré superlattice plays an important role in controlling the magnitude of anisotropic optical responses at the heterointerface. The symmetry breaking induced anisotropic behavior can exist at those interfaces stacked with the *C*_3_-symmetric monolayer TMDCs and *C*_2_-symmetric dielectrics, enabling a strategy to explore applications such as polarization-sensitive photodetectors^31^.

### Giant anisotropic conductance in SiP_2_-gated MoS_2_ transistors

**Fig. 3:**
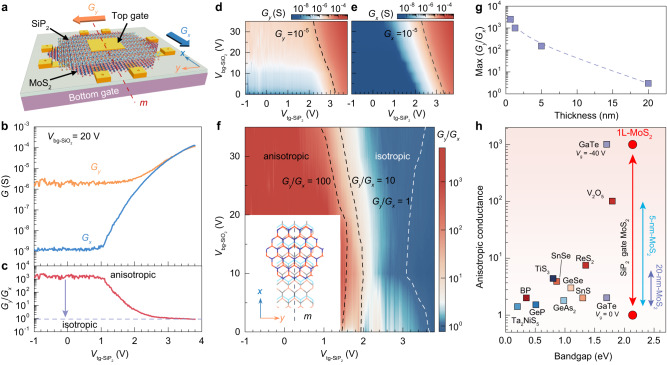
Gate tunable anisotropic transfer characteristics of MoS_2_/SiP_2_ heterointerfaces. **a** Schematic diagram of a SiP_2_-gated MoS_2_ transistor. The top gate medium is 20-nm-thick SiP_2_, and the bottom gate medium is 300-nm-thick SiO_2_. ${G}_{x}$ and ${G}_{y}$ are the sheet conductance along the *x* and *y* directions of the heterostructure, corresponding to the measurement geometries described in Supplementary Fig. 10. **b** Transfer characteristics of ${G}_{x}$ and ${G}_{y}$ for 1L-MoS_2_ at ${V}_{{{{{{\rm{bg}}}}}}{-{{{{{\rm{SiO}}}}}}}_{2}}$ = 20 V and 2 K. Note that ${V}_{{{{{{\rm{bg}}}}}}{-{{{{{\rm{SiO}}}}}}}_{2}}$ is necessarily applied to reduce contact resistance and activate top-gated channel for achieving measurable four-terminal conductance and a good signal-to-noise ratio. **c** Anisotropy index ${G}_{y}$/${G}_{x}$ as a function of ${V}_{{{{{{\rm{tg}}}}}}{-{{{{{\rm{SiP}}}}}}}_{2}}$ at ${V}_{{{{{{\rm{bg}}}}}}{-{{{{{\rm{SiO}}}}}}}_{2}}$ = 20 V. **d**, **e** ${G}_{y}$ (**d**) and ${G}_{x}$ (**e**) as a function of ${V}_{{{{{{\rm{tg}}}}}}{-{{{{{\rm{SiP}}}}}}}_{2}}$ and ${V}_{{{{{{\rm{bg}}}}}}{-{{{{{\rm{SiO}}}}}}}_{2}}$. **f** Color plot of anisotropy index ${G}_{y}$/${G}_{x}$ as a function of ${V}_{{{{{{\rm{tg}}}}}}{-{{{{{\rm{SiP}}}}}}}_{2}}$ and ${V}_{{{{{{\rm{bg}}}}}}{-{{{{{\rm{SiO}}}}}}}_{2}}$. The dashed lines highlight where ${G}_{y}$/${G}_{x}$ equals 1, 10, and 100. ${G}_{y}$/${G}_{x}$ values for ${V}_{{{{{{\rm{tg}}}}}}{-{{{{{\rm{SiP}}}}}}}_{2}}$ < 1.4 V and ${V}_{{{{{{\rm{bg}}}}}}{-{{{{{\rm{SiO}}}}}}}_{2}}$ < 20 V are not given since ${G}_{x}$ is too small to achieve measurable four-terminal conductance. The inset is a schematic illustration of the top view of a 1L-MoS_2_/SiP_2_ heterostructure. **g** The maximum ${G}_{y}$/${G}_{x}$ value of the MoS_2_/SiP_2_ device as a function of MoS_2_ thickness. **h** The anisotropic conductance in layered materials as a function of their bandgap values. Note that all other anisotropic materials host intrinsically-anisotropic conductance in nature while in our case we can drive the intrinsically-isotropic conductance in MoS_2_ into the anisotropic state. The values of anisotropic conductance and bandgaps for other anisotropic materials are generated from previous reports^18,35^.

To investigate the interfacial symmetry modulation on the electronic transport properties of MoS_2_, we measured the conductance ${G}_{x}$ (${G}_{y}$) along the *x* (*y*) directions of SiP_2_-gated 1L-MoS_2_ transistors (Fig. 3a, Supplementary Figs. 14 and 15). Figure 3b shows a comparison between ${G}_{x}$ and ${G}_{y}$ under different ${V}_{{{{{{\rm{tg}}}}}}{-{{{{{\rm{SiP}}}}}}}_{2}}$. One can see that the anisotropy index ${G}_{y}$/${G}_{x}$ can be as high as 10^3^ at the off-state with ${V}_{{{{{{\rm{tg}}}}}}{-{{{{{\rm{SiP}}}}}}}_{2}}$ < 1 V (Fig. 3c), implying that the symmetry engineering using SiP_2_ dielectric can drive the isotropic conductivity of *C*_3_-symmetric 1L-MoS_2_ into highly-anisotropic electronic states. With further increasing ${V}_{{{{{{\rm{tg}}}}}}{-{{{{{\rm{SiP}}}}}}}_{2}}$, the anisotropy index gradually approaches the value of 1, suggesting that 1L-MoS_2_ recovers back to isotopically conducting states at the on-state. Such continuous modulation of ${G}_{x}$, ${G}_{y}$, and ${G}_{y}$/${G}_{x}$ index can also be achieved at a wide range of ${V}_{{{{{{\rm{bg}}}}}}{-{{{{{\rm{SiO}}}}}}}_{2}}$ (Fig. 3d–f). The tunable conductance from anisotropic to isotropic characteristics suggests that SiP_2_ with in-plane anisotropy is anticipated to stimulate device functionality exploration for anisotropic digital inverters^32^, anisotropic memorizers^33^, or artificial synaptic devices^34^.

To confirm that such anisotropic conductance originates from the MoS_2_/SiP_2_ heterointerface, we compared the ${G}_{x}$ and ${G}_{y}$ values of SiP_2_-gated MoS_2_ transistors by increasing the thickness of MoS_2_ from monolayer to 20 nm. As a result, the observed anisotropy index ${G}_{y}$/${G}_{x}$ at the off-state decreases rapidly to ~1 when the thickness of MoS_2_ is increased to 20 nm (Fig. 3g, Supplementary Figs. 16 and 17), suggesting a nearly isotropic conductance in thicker samples. Such a thickness-dependent behavior is proposed to be attributed to the competition between the surface and bulk conductance, as qualitatively described in Supplementary Fig. 18. Specifically, the anisotropy index of MoS_2_ can be written as $\frac{{G}_{y}}{{G}_{x}}=\frac{{G}_{y}^{{{{{{\rm{surface}}}}}}}+{G}_{y}^{{{{{{\rm{bulk}}}}}}}}{{G}_{x}^{{{{{{\rm{surface}}}}}}}+{G}_{x}^{{{{{{\rm{bulk}}}}}}}}$, where bulk conductance (proportional to the sample thickness) is isotropic ${G}_{y}^{{{{{{\rm{bulk}}}}}}}$ ≈ ${G}_{x}^{{{{{{\rm{bulk}}}}}}}$ while surface conductance is anisotropic since only the surface layer of MoS_2_ with certain thickness (within the Thomas-Fermi screening length) can be tuned for carrier accumulation with SiP_2_ dielectric based on our numerical Poisson-Schrödinger calculations (Supplementary Figs. 19 and 20). For SiP_2_-gated 1L-MoS_2_ case, ${G}_{y}^{{{{{{\rm{bulk}}}}}}}$ = ${G}_{x}^{{{{{{\rm{bulk}}}}}}}$ = 0 and only the MoS_2_ layer on the interface (namely the whole monolayer) contributes to the conductance, so the anisotropy index can be written as $\frac{{G}_{y}}{{G}_{x}}=\frac{{G}_{y}^{{{{{{\rm{surface}}}}}}}}{{G}_{x}^{{{{{{\rm{surface}}}}}}}}$, whose value can be as high as 1000. When increasing the thickness of MoS_2_, ${G}_{y}^{{{{{{\rm{bulk}}}}}}}$ and ${G}_{x}^{{{{{{\rm{bulk}}}}}}}$ begin to increase and gradually dominate the total conductance with ${G}^{{{{{{\rm{surface}}}}}}}$ ≪ ${G}^{{{{{{\rm{bulk}}}}}}}$ at the thick limit. As a result, the anisotropy index is reduced to $\frac{{G}_{y}}{{G}_{x}}=\frac{{G}_{y}^{{{{{{\rm{bulk}}}}}}}}{{G}_{x}^{{{{{{\rm{bulk}}}}}}}}=1$, which is consistent with our experimental observation of less anisotropy in SiP_2_-gated MoS_2_ with a thickness of 20 nm. This result indicates that the anisotropic conductance behavior is contributed exactly from the interface of the MoS_2_/SiP_2_ heterostructures (details in Supplementary Figs. 18–20). Compared to those vdW materials with in-plane anisotropic crystal lattices and electronic structures^18,35^, our SiP_2_-gated 1L-MoS_2_ not only has the largest anisotropy index but also hosts the greatest capability to tune such an anisotropy index (Fig. 3h).

### Anisotropic moiré potential at 1L-MoS_2_/SiP_2_ interface

**Fig. 4:**
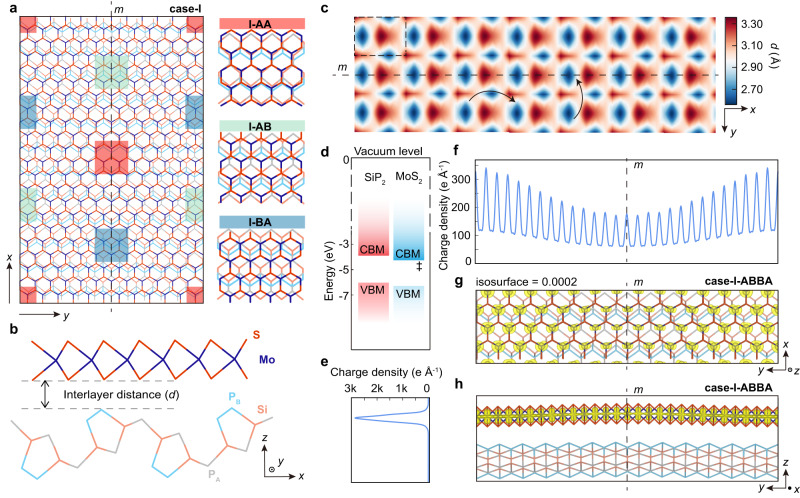
Structural and electronic properties of the MoS_2_/SiP_2_ heterointerface. **a** Moiré pattern of case-I formed by stacking MoS_2_ monolayer on SiP_2_ monolayer. The colored rectangles mark different stacked configurations: I-AA (red area), I-AB (green area), and I-BA (blue area). The vertical dashed line represents the mirror plane *m*. **b** The side view of the case-I heterostructure model. The interlayer distance is marked by the black lines, which corresponds to the difference between the *z* coordinates of adjacent S atoms and P atoms in the vdW gap. **c** The real space distribution of the interlayer distance in the moiré pattern of case-I. The dashed rectangular area corresponds to the moiré superlattice shown in (**a**). The effective hopping paths between the trapped states along the *x* and *y* directions are shown by the black curve arrows. **d** The band alignment between unstrained monolayer MoS_2_ and unstrained monolayer SiP_2_. CBM and VBM represent the conduction band minimum and valence band maximum, respectively. The work function and bandgap for each layer are calculated with the HSE-06 functional. The conduction band edge contributed by monolayer MoS_2_ is marked by “‡”. **e**, **f** The illustrations of the plane-averaged charge density on the lowest conduction band edge contributed by 1L-MoS_2_ along the *z* direction (**e**) and *y* direction (**f**) of the case-I-ABBA heterostructure. **g**, **h** Top view (**g**) and side view (**h**) for the calculated charge density for the lowest conduction band edge contributed by monolayer MoS_2_ in case-I-ABBA heterostructure. The isosurface is set as 0.0002 e Å^–3^.

To further understand the influence of interface structures on the anisotropic optical and transport behavior, we explore the structural and electronic properties of the 1L-MoS_2_/SiP_2_ heterostructure by using density functional theory (DFT) calculations. Note that the mirror symmetry of the constructed MoS_2_/SiP_2_ heterointerface originates from the parallel or antiparallel alignment of the zigzag chain in MoS_2_ along the *y* direction of SiP_2_ (see details in Supplementary Fig. 21). Thus, two kinds of moiré patterns can be obtained (labeled as case-I and case-II, details in Section 13 in Supplementary Information). Taking the moiré pattern of case-I as an example (Fig. 4a, b), due to the lattice mismatch between MoS_2_ and SiP_2_, there are three typical stacking structures, labeled as I-AA, I-AB, and I-BA, as indicated by the colored rectangular areas in Fig. 4a. The details about atomic stacked configurations and stacked structures with II-AA, II-AB, and II-BA in the moiré pattern of case-II are shown in Supplementary Fig. 21d.

The structural corrugations in 1L-MoS_2_/SiP_2_ heterostructure with two kinds of moiré patterns are simulated via DFT calculations (Fig. 4c and Supplementary Fig. 27b, e). After being placed on the SiP_2_ lattice, the atomic flat structure of 1L-MoS_2_ will be deformed due to the interface coupling, resulting in the formation of moiré potential on 1L-MoS_2_ that effectively breaks *C*_3_-rotational symmetry of the pristine MoS_2_. In contrast, the structural corrugations in 1L-SiP_2_ are much smaller compared with that in 1L-MoS_2_ (Supplementary Fig. 25). Furthermore, we plot the distribution of the interlayer distance (marked in Fig. 4b) for the moiré pattern of case-I in Fig. 4c to demonstrate the moiré potential in this heterostructure. In the moiré pattern of case-I, the stacked region of I-BA hosts the smallest interlayer distance between MoS_2_ and SiP_2_, indicating the largest structural deformation on 1L-MoS_2_ and interlayer coupling. However, in the moiré pattern of case-II such region with the largest moiré potential and lattice deformation becomes II-AA (Supplementary Fig. 27e). On the other hand, the mirror symmetry parallel to the armchair direction of 1L-MoS_2_ (also the *x* direction of the heterostructure) is always observed in both moiré patterns of 1L-MoS_2_/SiP_2_ heterostructures that is determined by the specific stacked regulations in the fabrication of experimental devices. Such characters with reduced symmetry in corrugated 1L-MoS_2_ are consistent with the symmetry analysis to stacking structures with moiré patterns. Our simulated structural deformation of corrugated 1L-MoS_2_ with moiré patterns gives a consistent interpretation on the change of the symmetric shape of the experimental SHG spectra from sixfold (1L-MoS_2_) to twofold (1L-MoS_2_/SiP_2_).

The out-of-plane structural corrugations of 1L-MoS_2_ with moiré patterns can strongly modulate its electronic structures and thus influence the optical properties. Since the direct simulation of the large-size moiré lattice by using DFT are too expensive to afford, to overcome this issue, we use a strained MoS_2_ and SiP_2_ to construct a heterostructure guaranteeing the conduction band offset between MoS_2_ and SiP_2_ same with that in the moiré heterostructure, then simulate the influence of the moiré potential on the electronic states of 1L-MoS_2_ (details in Sections 14–17 in Supplementary Information). The exemplified results are presented in the heterostructure model with stacking regions of I-AB and I-BA (named as case-I-ABBA). Figure 4g and h demonstrates the charge density distribution for conduction band edge in case-I-ABBA, and Fig. 4e and f shows the plane-averaged charge density along the *z* and *y* directions (Section 17 in Supplementary Information). In the heterostructure model with fully relaxation, out-of-plane corrugation can be clearly observed with the retaining of the mirror symmetry in the moiré pattern. The conduction band edge in 1L-MoS_2_/SiP_2_ heterostructure is dominated by the state from the MoS_2_ layer, while its charge density distribution has been strongly modified by the lattice deformation. The calculated results for other heterostructure models containing different stacked regions are shown in Section 17 in Supplementary Information. The conduction band edge in 1L-MoS_2_/SiP_2_ heterostructure is always strongly modified by the moiré potential. For the case-II moiré pattern, we build similar models and obtain the same conclusions (details in Sections 16 and 17 in Supplementary Information). Compared with pristine 1L-MoS_2_ with *C*_3_-rotational symmetry, the symmetry engineering on the conduction band edge can be clearly observed in 1L-MoS_2_/SiP_2_ heterostructure. With breaking *C*_3_-rotational symmetry by SiP_2_, the conduction band edge on deformed 1L-MoS_2_ only keeps the mirror symmetry (Fig. 4f). Therefore, when one electron is excited on conduction band edge and couples with hole states, the optical matrix elements in formed exciton should be strongly modified by the moiré potential with lower symmetry and the lowest bright exciton absorption becomes highly anisotropic, which is consistent with the observation from our PL experiments.

The formation of moiré potential with symmetry engineering can also explain the experimentally observed giant anisotropic conductance in SiP_2_-gated MoS_2_ transistors. At the off-state with low carrier density (~5 × 10^9 ^cm^–2^) and low temperature (2 K), the charge carriers in 1L-MoS_2_/SiP_2_ heterostructure are mainly trapped by charged impurities^22,36–38^ and the moiré potentials, thus the giant anisotropic conductance of 1L-MoS_2_ is mainly contributed by the effective hopping between trapped charge states in the moiré potentials^22,36–38^. Since the charged defects in 1L-MoS_2_ are distributed randomly without anisotropy, the anisotropic moiré potential should be a critical factor for the anisotropic conductance in the SiP_2_-gated MoS_2_ transistor. Similar to previous discussions, we also take the moiré pattern of case-I as an example (the discussion of the moiré pattern of case-II draws the same conclusion), the distribution of interlayer distance between SiP_2_ and MoS_2_ in real space (Fig. 4c) shows that the smallest interlayer distance is located in the I-BA stacking region, which corresponds to the largest interlayer potential and can effectively trap the charge carriers inside. On the other hand, the anisotropic moiré potential results in highly anisotropic hopping between trapped states. For example, the effective hopping along the parallel direction (||) to the mirror plane is naturally smaller than that along the perpendicular direction (⊥), indicating that, at the off state, the effective mass of these trapped states is highly anisotropic (${m}_{\parallel }\gg {m}_{\perp }$). The large ratio of effective mass (${m}_{\parallel }$/${m}_{\perp }$) leads to the highly-anisotropic conductance in 1L-MoS_2_/SiP_2_ heterostructure. With increasing the electron density to reach the on-state, the moiré potential cannot fully trap these charge carriers and its influence on transport becomes unimportant, delocalizing the 2D electron gas formed on the 1L-MoS_2_ layer. Thus, the conductance turns out to be isotropic at the on-state.

## Discussion

In conclusion, we demonstrate an anisotropic van der Waals dielectric SiP_2_ that can simultaneously tune the electronic states of channel semiconductors and induce symmetry engineering at TMDC/SiP_2_ interfaces. Our first-principles calculations reveal that these anisotropic characteristics originate from the formation of the anisotropic-symmetric moiré potential in the MoS_2_/SiP_2_ heterostructure, which strongly modulates structural and electronic properties of 1L-MoS_2_ with tunable anisotropic symmetry. The tunable interfacial symmetry in the TMDC/SiP_2_ heterostructure can provide a unique platform for investigating symmetry-related interfacial physics and corresponding phenomena, including the generation of the in-plane polarization^5^ (bulk photovoltaic effect and quantum shift current) and the Berry curvature dipole^39–41^ (circular photo-galvanic effect and nonlinear Hall effect). The giant anisotropy generated in the TMDC/SiP_2_ heterostructure, which is absent in the pristine TMDC material, sheds light on the moiré physics of the engineered interface with reduced symmetry, and provides an effective way to control the degree of freedom of electrons in condensed matter systems.

## Methods

### Crystal symmetry analyses of TMDC/SiP_2_ heterostructures

The orthorhombic SiP_2_ crystal exhibits an anisotropic layered structure (space group $P{nma}$) with an embedded quasi-one-dimensional P_B_–P_B_ chains along the *y* direction of the crystal lattice. Specifically, three important spatial symmetry operations should be addressed in this atomic structure of the SiP_2_ crystal when stacking SiP_2_ with monolayer TMDCs. First, there is a non-symmorphic *C*_2_ symmetry about the *z* direction (the screw symmetry *S*_2_ that combines a twofold rotational symmetry with a translation along the *z* direction in the half-unit-cell, *S*_2_ = *C*_2_ + *z*/2) in the SiP_2_ crystal. This non-symmorphic *C*_2_ symmetry of bulk SiP_2_ is incompatible with *C*_3_ symmetry of TMDCs and will result in highly anisotropic nature (*C*_1_ symmetry) of the TMDC/SiP_2_ heterostructures. Second, the atomic structure of SiP_2_ is inversion symmetric, which means there is no SHG signal of SiP_2_ flakes, ensuring that the distorted SHG signals at the TMDC/SiP_2_ heterostructure mainly come from the symmetry breaking at the heterointerface. Third, there is a mirror symmetry perpendicular to the P_B_–P_B_ chains (*y* direction) of the SiP_2_ crystal. This vertical mirror can remain in the TMDC/SiP_2_ heterostructures when stacking the TMDCs and SiP_2_ by aligning their mirror planes (the zigzag direction of TMDCs is parallel to the P_B_–P_B_ chains of SiP_2_), generating a mirror symmetric anisotropic moiré potential at the heterointerface.

### Optical measurements of TMDC/SiP_2_ heterointerfaces

SiP_2_ flakes and TMDC flakes were prepared by mechanical exfoliation onto polydimethylsiloxane (PDMS) stamps and SiO_2_/Si wafers (300-nm-thick SiO_2_ layer). TMDC/SiP_2_ heterointerfaces were fabricated using a dry-transfer method and stacked by parallelly aligning the zigzag direction of MoS_2_ and *y* direction of SiP_2_. The crystal axes of the 1L-MoS_2_ samples are confirmed by their polarized-SHG results. And the crystal axes of SiP_2_ are first identified by their optical image and then confirmed by their polarized PL results. The whole sample fabrication was processed in a glove box to avoid any degradation. Room temperature PL measurements were performed using a confocal Raman system (WITec Alpha 300) using a 50× objective lens with an incident laser (laser power of 1 mW) focused to a 1 μm spot size. Nitrogen-filled environments were established by protecting samples with continuous nitrogen gas flow. Low-temperature PL measurements were performed under vacuum conditions in cryostats (Cryo Instrument of America RC102–CFM Microscopy Cryostat). For polarized PL measurements, the excitation polarization is fixed along the *x* direction, and the detection polarization is changed from *θ* = 0° to 180° (*θ* is the angle between the detection polarization and the *x* direction).

The SHG measurements were performed using a Ti:sapphire oscillator with an excitation wavelength of 810 nm, pulse width of 70 fs, and repetition rate of 80 MHz. The laser pulse was focused to an ~1 μm spot size by a 40× objective lens. The SHG signals are obtained under a configuration with the detection polarization parallel to the excitation polarization. For the SHG signal of 1L-TMDCs on the SiO_2_ substrate, the sixfold symmetric SHG intensities are fitted by Eq. (1):

1 $${I}_{{{{{{\rm{SHG}}}}}}}^{\parallel }\propto {\cos }^{2}3\theta$$

Normally, for those uniaxially-strained TMDCs, the SHG intensities ${I}_{{{{{{\rm{SHG}}}}}}}^{\parallel }$ parallel to the incident laser polarization can be written as:

2 $${I}_{{{{{{\rm{SHG}}}}}}}^{\parallel }\propto {\left[\cos 3\theta+{\varepsilon }_{y}\left({k}_{1}{\cos }^{3}\theta -{k}_{2}{\sin }^{2}\theta \cos \theta \right)\right]}^{2}$$

where ${\varepsilon }_{y}$ is the strain along the *y* direction, ${k}_{1}$ and ${k}_{2}$ are parameters related to TMDCs. In our case of the 1L-MoS_2_/SiP_2_, the *C*_3_ symmetry of 1L-MoS_2_ is also reduced to low symmetry such as *C*_1_. Therefore, we fit our data by Eq. (2) to describe the anisotropic SHG response in our 1L-MoS_2_/SiP_2_.

### Electrical transport measurements

The 1L-MoS_2_ (or few-layer MoS_2_) and SiP_2_ flakes for electronic transport measurements were exfoliated onto a PDMS stamp and transferred onto a silicon substrate with prepatterned electrodes (Ti/Au with a thickness of 3/9 nm) in sequence. A top gate on the SiP_2_ flake (Ti/Au with a thickness of 5/45 nm) was then made using electron-beam lithography and electron-beam evaporation. Electrical transport measurements were performed in a cryo-free superconducting magnet system (Oxford Instruments Teslatron^PT^). Four-terminal resistance ${R}_{{xx}}$ was acquired using a Keithley 2182 voltmeter with a DC current supplied by a Keithley 2400 sourcemeter. The gate voltage is supplied by a Keithley 2400 sourcemeter. The sheet carrier density (${n}_{2{{{{{\rm{D}}}}}}}$) is obtained based on Hall effect measurements on Au/SiP_2_/MoS_2_ sandwiched devices^22^. The Au/SiP_2_/MoS_2_ device can be considered as a parallel plate capacitor, and the amount of charge per unit area can be written as:

3 $$e{n}_{2{{{{{\rm{D}}}}}}}=\frac{{\varepsilon }_{0}{\varepsilon }_{{{{{{\rm{r}}}}}}-{{{{{\rm{Si}}}}}}{{{{{{\rm{P}}}}}}}_{2}}}{{t}_{{{{{{\rm{Si}}}}}}{{{{{{\rm{P}}}}}}}_{2}}}{V}_{{{{{{\rm{tg}}}}}}-{{{{{\rm{Si}}}}}}{{{{{{\rm{P}}}}}}}_{2}}$$

where $e$ is the electron charge, ${\varepsilon }_{0}$ is the vacuum permittivity, ${t}_{{{{{{\rm{Si}}}}}}{{{{{{\rm{P}}}}}}}_{2}}$ = 20 nm is the thickness of SiP_2_, and ${\varepsilon }_{{{{{{\rm{r}}}}}}-{{{{{\rm{Si}}}}}}{{{{{{\rm{P}}}}}}}_{2}}$ is the relative dielectric constant of SiP_2_ within the Au/SiP_2_/MoS_2_ sandwiched structure. The ${\varepsilon }_{{{{{{\rm{r}}}}}}-{{{{{\rm{Si}}}}}}{{{{{{\rm{P}}}}}}}_{2}}$ is obtained by linear fitting of Eq. (3).

### DFT calculations

The Vienna Ab initio Simulation package (VASP)^42^ was used for the first-principles calculations. The generalized-gradient approximation (GGA) of the Perdew-Burke-Ernzerhof-type functional^43^ was used with the projected-augmented-wave method^44,45^ and an energy cutoff of 500 eV. For the electronic self-consistent calculations, the convergence criterion was set as 10^–6^ eV. Considering the van der Waals interaction, the DFT-D3 method^46^ was applied as the correction. The force criterion was chosen to be 0.01 eV Å^–1^. After fully relaxing the 2H-MoS_2_ bulk structure, we obtained the lattice constants of 3.16 Å for *a* (and *b*) and 12.39 Å for *c*, which were consistent with previous studies^47,48^. The *k*-point mesh of 15 × 15 × 3 was used to sample the Brillouin zone (BZ). After the full relaxation of the SiP_2_ bulk structure, the lattice constants were 10.11 Å for *a*, 3.44 Å for *b*, and 14.18 Å for *c*, which were consistent with the experimental results^49^. The *k*-point mesh of 5 × 15 × 4 was used to sample the BZ of bulk SiP_2_. Moreover, except for the structural relaxation, the spin-orbit coupling was considered in all DFT calculations. For all slab models used in this work, a vacuum layer with 20 Å was added along the *z* direction. Moreover, the calculations with the HSE06 functional^50,51^ were performed to obtain the accurate values of the work function and bandgap of the unstrained and strained MoS_2_ and SiP_2_ monolayers.

For these heterostructure models with strained MoS_2_ and SiP_2_ which were used to simulate the electronic properties of 1L-MoS_2_/SiP_2_ heterostructure directly, the $2\sqrt{3}\times 1$ rectangle supercell for monolayer MoS_2_ was used and its lattice was tensed to 3.25 Å for *a*, and was compressed to 10.69 Å for *b*. Correspondingly, the lattice of monolayer SiP_2_ was enlarged to 3.50 Å for *a* and 10.69 Å for *b*. In each strained heterostructure model, it contains 13 × 1 monolayer SiP_2_ unitcells and 14 × 1 rectangle supercells for monolayer MoS_2_. The *k*-point mesh of 5 × 1 × 1 was used to sample the BZ of heterostructure model. For strained case-I-ABBA and case-II-AABA lattices, we fully relaxed the whole structures. While, for strained case-I-AA and case-II-AB lattices, we fixed the *x* and *y* coordinates of the two central S atoms in MoS_2_ layer and one central P atom in SiP_2_ layer in the AA/AB stacking region. Then we fully relaxed other atoms in the strained heterostructure model.

### Supplementary information

Supplementary Information

Peer review file

## Data Availability

The Source Data underlying the figures of this study are available at 10.6084/m9.figshare.23623722. All raw data generated during the current study are available from the corresponding authors upon request.
