# Supplementary material for: An anisotropic van der Waals dielectric for symmetry engineering in functionalized heterointerfaces
Source: Nat Commun. 2023 Sep 9;14:5568. doi: 10.1038/s41467-023-41295-6 (PMC10492835; doi:10.1038/s41467-023-41295-6)
Supplement: Supplementary file 1 — Supplementary Information [file 41467_2023_41295_MOESM1_ESM.pdf]

Supplementary Information for

An Anisotropic van der Waals Dielectric for Symmetry Engineering in Functionalized Heterointerfaces

Zeya Li[1,2†], Junwei Huang[1,2†], Ling Zhou[1,2†], Zian Xu[3†], Feng Qin[1,2], Peng Chen[1,2], Xiaojun Sun[1,2], Gan Liu[1,4], Chengqi Sui[1,2], Caiyu Qiu[1,2], Yangfan Lu[5], Huiyang Gou[6], Xiaoxiang Xi[1,4], Toshiya Ideue[7,8]*, Peizhe Tang[3,9]*, Yoshihiro Iwasa[7,10], Hongtao Yuan[1,2]*

[1] *National Laboratory of Solid State Microstructures, and Collaborative Innovation Center of Advanced Microstructures, Nanjing University, Nanjing 210093, China.*

[2] *College of Engineering and Applied Sciences, and Jiangsu Key Laboratory of Artificial Functional Materials, Nanjing University, Nanjing 210023, China.*

[3] *School of Materials Science and Engineering, Beihang University, Beijing 100191, China.*

[4] *School of Physics, Nanjing University, Nanjing 210093, China.*

[5] *College of Materials Sciences and Engineering, National Engineering Research Center for Magnesium Alloys, Chongqing University, Chongqing 400030, China.*

[6] *Center for High Pressure Science and Technology Advanced Research, Beijing 100094, China.*

[7] *Quantum Phase Electronic Center and Department of Applied Physics, The University of Tokyo, Tokyo 113-8656, Japan.*

[8] *Institute for Solid State Physics, The University of Tokyo, Chiba 277-8581, Japan.*

[9] *Max Planck Institute for the Structure and Dynamics of Matter, Center for Free Electron Laser Science, Hamburg 22761, Germany.*

[10] *RIKEN Center for Emergent Matter Science, Hirosawa 2-1, Wako 351-0198, Japan.*

[†] These authors contributed equally to this work.

E-mail: htyuan@nju.edu.cn (H.T.Y.); peizhet@buaa.edu.cn (P.T.); ideue@issp.u-tokyo.ac.jp (T. I.).

Outline:

1. **Symmetry and its roles in electrical conductance in crystals**
2. **Transfer characteristics of SiP_2-gated MoS_2 transistors**
3. **Gate-tuned activation energy in 1L-MoS_2/SiP_2 device**
4. **Estimation of the dielectric constant of SiP_2 via Hall measurements**
5. **Comparisons of the anisotropic effective mass and the dielectric constant between SiP_2 and other dielectric materials**
6. **Berry curvature dipole and circular photo-galvanic effect in symmetry-mismatched heterointerface**
7. **Linear dichroic PL spectra generated in 1L-MoS_2 and 1L-WS_2 via symmetry engineering**
8. **SHG signals and rotational symmetry of 1L-WS_2/SiP_2 and 1L-MoS_2/SiP_2**
9. **Twist-angle dependent anisotropic SHG and PL responses in 1L-MoS_2/SiP_2**
10. **Anisotropic transport properties of 1L-MoS_2 device gated with SiP_2 dielectric**
11. **Thickness-dependent anisotropic conductance in SiP_2-gated MoS_2 devices**
12. **Energy band alignment and carrier density profile calculated with the Poisson-Schrödinger equation**
13. **The detailed illustration of the moiré patterns of case-I and case-II**
14. **Band alignment of the unstrained 1L-MoS_2/SiP_2 heterostructure**
15. **The construction of the heterostructure models used in the DFT calculations**
16. **The relaxation of the heterostructure models for the DFT calculations**
17. **The electronic structures of the heterostructures in the DFT calculations**
18. **The roles of the interlayer distance and the charge density distribution on the conduction band edge of the moiré superlattices of case-I and case-II**

1. Symmetry and its roles in electrical conductance in crystals

When an electric field $\boldsymbol{E}$ is applied to a material, an electrical current density $\boldsymbol{j}$ will be generated, and the motion of free charges can be characterized by the electrical conductance tensor σ through Ohm's law[1]:

$$\boldsymbol{j} = \sigma \boldsymbol{E} \quad (1)$$

The electrical conductance tensor σ can be regarded as a scalar if the material is isotropic. Based on the spatial symmetry of crystals, the direction and magnitude of the electrical current density for certain crystals can be determined by the direction of the applied external electric field:

$$j_a = \sigma_{ab} E_b \quad (2)$$

where the subindices a and b denote the in-plane components of the Cartesian coordinate in two dimensions. Note that the Einstein summation convention is applied here, and the summation is understood over all three components of Cartesian coordinates for those subindices appearing twice within a single term.

Mathematically, each element of the electrical conductance tensor σ_{ab} can be qualitatively known to be zero or nonzero, and σ_{ab} should be invariant under the symmetry operation of the crystal. Importantly, for the system holding the time reversal symmetry, Onsage's reciprocal law indicates that the electrical conductance tensor σ_{ab} must be symmetric, that is $\sigma_{ab} = \sigma_{ba}$. Therefore, we only consider the spatial symmetry of the crystal below, such as the rotation and the reflection operations.

For the rotation symmetry along the z-axis (labeled as R_z), the rotation operation for the φ angle takes the form:

$$R_z(\varphi) = \begin{pmatrix} \cos\varphi & \sin\varphi \\ -\sin\varphi & \cos\varphi \end{pmatrix} \quad (3)$$

For the vertical mirror symmetry perpendicular to the y-axis M_y, the reflection operation for the mirror takes the form:

$$M_y = \begin{pmatrix} 1 & 0 \\ 0 & -1 \end{pmatrix} \quad (4)$$

Therefore, σ_{ab} should be invariant under the rotation operation

$$\sigma = R_z^{-1}(\varphi)\sigma R_z(\varphi) \quad (5)$$

and the reflection operation

$$\sigma = M_y^{-1}\sigma M_y \quad (6)$$

Note that the electrical conductance tensor σ is symmetric and positive semidefined, and there always exist two orthogonal main axes that diagonalize the σ. On such a basis, one can clearly see that, only the materials with rotation operations of one and two folds are anisotropic, and the material with rotation operations of higher folds must be isotropic.

Specifically, in our case, the isotropic conductance in 1L-MoS_2 is protected by its C_{3v} symmetry, while once the MoS_2 is placed on the SiP_2, the symmetry of the heterostructure reduces to C_1 symmetry, which is expected to result in the anisotropic transport in 1L-MoS_2/SiP_2. Such phenomenological descriptions cannot reveal detailed information on the magnitude of elements of the electrical conductance tensor σ_{ab} and their dependence on other tuning parameters, such as temperature and gate voltage. The magnitude of σ_{ab} is the main target of the theory of the microscopic mechanism for electrical conductance.

Supplementary Table 1: The form of the electrical conductance tensor σ for various crystal symmetries

Crystal symmetry	Rotation	Reflection	Electrical conductance	Property
C_1	$R(2\pi)$	–	$\sigma = \begin{pmatrix} \sigma_{11} & \sigma_{12} \\ \sigma_{12} & \sigma_{22} \end{pmatrix}$	Anisotropic
C_{1v}	$R(2\pi)$	M_y	$\sigma = \begin{pmatrix} \sigma_{11} & 0 \\ 0 & \sigma_{22} \end{pmatrix}$	Anisotropic
C_2	$R(\pi)$	–	$\sigma = \begin{pmatrix} \sigma_{11} & \sigma_{12} \\ \sigma_{12} & \sigma_{22} \end{pmatrix}$	Anisotropic
C_{2v}	$R(\pi)$	M_y	$\sigma = \begin{pmatrix} \sigma_{11} & 0 \\ 0 & \sigma_{22} \end{pmatrix}$	Anisotropic
C_3	$R(2\pi/3)$	–	$\sigma = \begin{pmatrix} \sigma_{11} & 0 \\ 0 & \sigma_{11} \end{pmatrix}$	Isotropic
C_{3v}	$R(2\pi/3)$	M_y	$\sigma = \begin{pmatrix} \sigma_{11} & 0 \\ 0 & \sigma_{11} \end{pmatrix}$	Isotropic
C_4	$R(\pi/2)$	–	$\sigma = \begin{pmatrix} \sigma_{11} & 0 \\ 0 & \sigma_{11} \end{pmatrix}$	Isotropic
C_{4v}	$R(\pi/2)$	M_y	$\sigma = \begin{pmatrix} \sigma_{11} & 0 \\ 0 & \sigma_{11} \end{pmatrix}$	Isotropic
C_6	$R(\pi/3)$	–	$\sigma = \begin{pmatrix} \sigma_{11} & 0 \\ 0 & \sigma_{11} \end{pmatrix}$	Isotropic
C_{6v}	$R(\pi/3)$	M_y	$\sigma = \begin{pmatrix} \sigma_{11} & 0 \\ 0 & \sigma_{11} \end{pmatrix}$	Isotropic

2. Transfer characteristics of SiP_2-gated MoS_2 transistors

To evaluate the gating tunability of the SiP_2 dielectric, we performed electrical transport measurements under dual-gate configurations in MoS_2/SiP_2 field-effect transistor (FET) devices on a SiO_2/Si substrate (Supplementary Fig. 1a). Supplementary Figure 1b shows the transfer curve obtained by sweeping voltages of the top SiP_2 gate ($V_{\mathrm{tg-SiP_2}}$) at various voltages of the back SiO_2 gates ($V_{\mathrm{bg-SiO_2}}$) in a 5-nm-MoS_2/SiP_2 transistor. When $V_{\mathrm{bg-SiO_2}}$ = 0 V, the on/off ratio of MoS_2 can reach 10^4 with $V_{\mathrm{tg-SiP_2}}$ lower than 5 V. After increasing $V_{\mathrm{bg-SiO_2}}$ to 35 V, the on-state current can further increase. The four-probe conductance G exhibits a similar transfer characteristic and results in an on/off ratio of 10^4, which indicates the good ohmic contact of our MoS_2 dual-gated transistor (Supplementary Fig. 1c). Supplementary Fig. 2a shows the transfer characteristics of 1L-MoS_2 transistors with a SiP_2 top gate. With sweeping $\mathrm{V_{tg-SiP_2}}$ up to 4 V, the 1L-MoS_2 transistor shows an on/off ratio as high as 10^5 (similar to the 5-nm-MoS_2). The measured leakage current density of the SiP_2-gated 1L-MoS_2 transistor is as small as approximately 10^{-5} A cm^{-2} at an external electric field strength of 1.2 MV cm^{-1} (Supplementary Fig. 2b).

Based on gate-dependent four-terminal conductance G, the field-effect mobility μ can be extracted using the expression $\mu = [dG/dV_{\mathrm{tg-SiP_2}}]\times[L_{12}/(WC_{\mathrm{SiP_2}})]$,[2] where L_{12} = 4 μm and W = 11 μm are the length and width of the channel, respectively. The capacitance of SiP_2, $C_{\mathrm{SiP_2}} = \varepsilon_0\varepsilon_\mathrm{r}/t_{\mathrm{SiP_2}}$, where ε_0 is the vacuum permittivity, $t_{\mathrm{SiP_2}}$= 20 nm is the thickness of SiP_2, and ε_r = 8.1 is the dielectric constant of SiP_2. When $V_{\mathrm{bg-SiO_2}}$ = 0 V, the field-effect mobility of the 5-nm-thick MoS_2 can reach approximately 200 cm^2 V^{-1} s^{-1} with $V_{\mathrm{tg-SiP_2}}$ lower than 5 V. After increasing $V_{\mathrm{bg-SiO_2}}$ to 35 V, the field-effect mobility of MoS_2 further increased to about 600 cm^2 V^{-1} s^{-1} (Fig. 1e in main text). More interestingly, in another 1L-MoS_2/SiP_2 device, a maximum μ value of 330 cm^2 V^{-1} s^{-1} at 2 K can be reached in such a dual-gate configuration (Supplementary Fig. 2c), which is comparable to the high mobility reported in HfO_2-gated 1L-MoS_2 devices[2] (174 cm^2 V^{-1} s^{-1} at 4 K). Such high mobility in few-layer MoS_2 indicates that the SiP_2 dielectric, as a typical vdW material, might form an atomically-flat interface and reduce charge scattering[3,4] in MoS_2, preserving the intrinsic properties of 2D materials and allowing the fabrication of high-performance devices. Supplementary Table 2 summarizes typical parameters of the MoS_2 FETs based on different dielectrics, including the on/off ratio, field-effect mobility, operation voltage, and breakdown field. We find that our SiP_2-gated MoS_2 FETs host a high on/off ratio, high mobility, low operation voltage, and large breakdown field,

which are comparable to those of widely-used high-κ dielectric gated MoS_2 FETs[2,5-8].

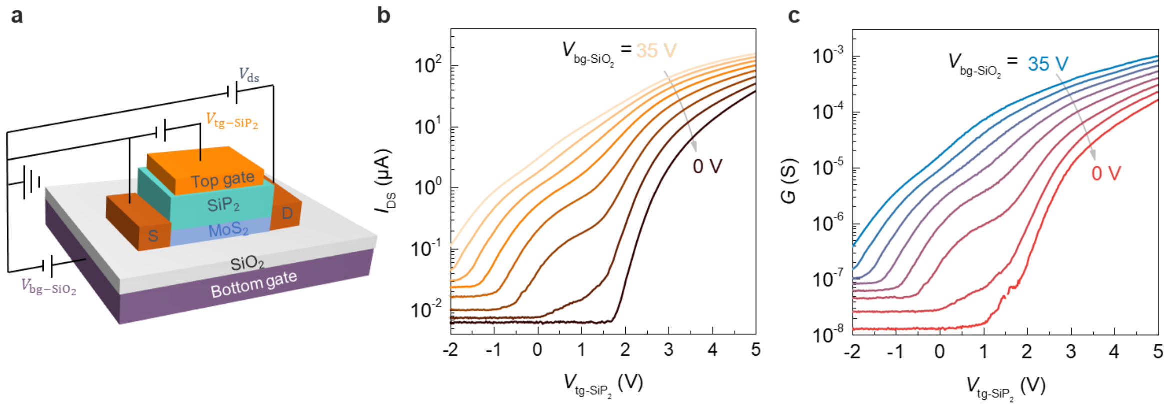

Supplementary Figure 1. Transfer curves of a MoS_2 dual-gated transistor. a, Schematic illustration of the MoS_2/SiP_2 dual-gated device. **b**, Drain current versus top gate (SiP_2) voltage for a 5-nm-MoS_2 transistor under different back gate (SiO_2) voltages. **c**, Four-probe conductance G as a function of V_{tg-SiP_2} acquired at different back gate voltages.

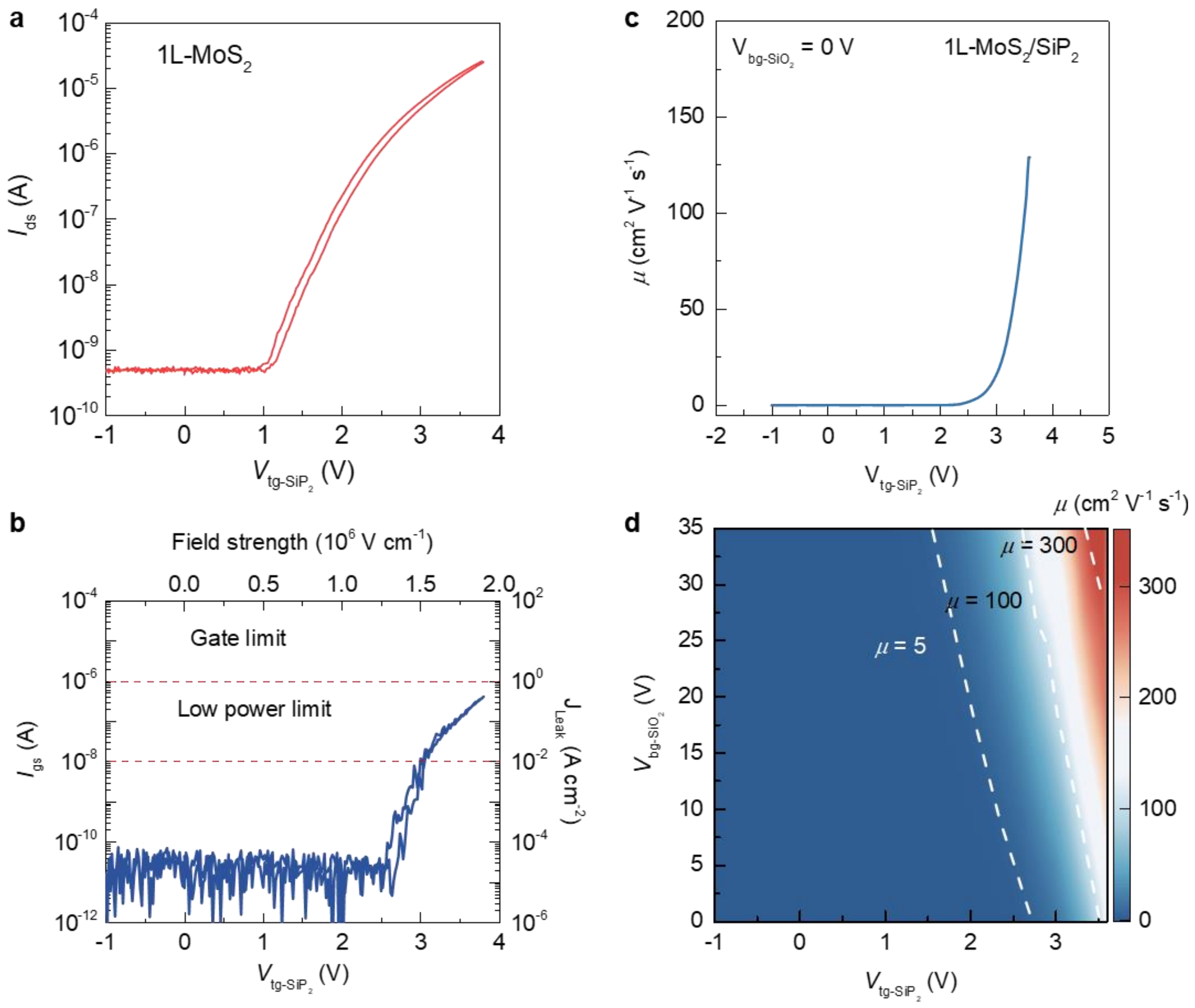

Supplementary Figure 2. Transfer characteristic and field effect mobility of a SiP_2-gated 1L-MoS_2 transistor. a, Transfer curves of the 1L-MoS_2-based transistor at 2 K when sweeping V_{tg-SiP_2}. **b**, Leakage current I_{gs} as a function of V_{tg-SiP_2} on a 1L-MoS_2 device at 2 K. I_{gs} and V_{tg-SiP_2} are rescaled to the leakage current density (J_{Leak}) and the electric field strength for a better comparison (right and top axes). Horizontal red lines mark the limits of leakage current density for various types of integrated circuits. **c**, Field effect mobility μ as a function of V_{tg-SiP_2} at fixed V_{bg-SiO_2} = 0 V for a 1L-MoS_2/SiP_2 device. **d**, Field effect mobility μ as a function of V_{tg-SiP_2} and V_{bg-SiO_2} for a 1L-MoS_2/SiP_2 device.

Supplementary Table 2. Comparison of 1L-MoS_2 FETs gated with different dielectrics

Dielectrics	On/off ratio	Mobility ($cm^2\ V^{-1}\ s^{-1}$)	E_{bd} (MV cm^{-1})	Operation voltage (V)	Refs.
SiP_2	10^5	300 (2 K)	> 2	< 5	This work
SiO_2	10^3–10^5	~1 (300 K)<br>~20 (40 K)	> 3	~ 80	6,9
HfO_2	10^8	174 (4 K)	> 2	< 5	2
Al_2O_3	10^7	28 (300 K)	> 4.5	< 3	5
h-BN	10^5	60 (300 K)<br>1000 (5 K)	> 2	< 4	3,10
Sb_2O_3	10^8	80 (40 K)	1.8	> 20	6
FS-$SrTiO_3$	10^7	40 (300 K)	> 2	< 2	7
CaF_2	10^4–10^7	NA	10–15	< 2	8

* NA: not applicable. E_{bd}: breakdown field.

3. Gate-tuned activation energy in 1L-MoS_2/SiP_2 device

To further understand the insulator-to-metal transition in the SiP_2-gated 1L-MoS_2 device, we replot the temperature-dependent resistance at varying V_{tg-SiP_2} in an Arrhenius plot[11]. As shown in Supplementary Fig. 3a, the resistance follows a thermal activation dependence. We extract the activation energy for charge transport in Supplementary Fig. 3b. The activation energy decreases monotonically from ~ 7 meV to near 0 meV when increasing V_{tg-SiP_2} from 2.5 V to 3.8 V. Such an activation energy decrease directly indicates the insulator-to-metal transition in 1L-MoS_2/SiP_2 device.

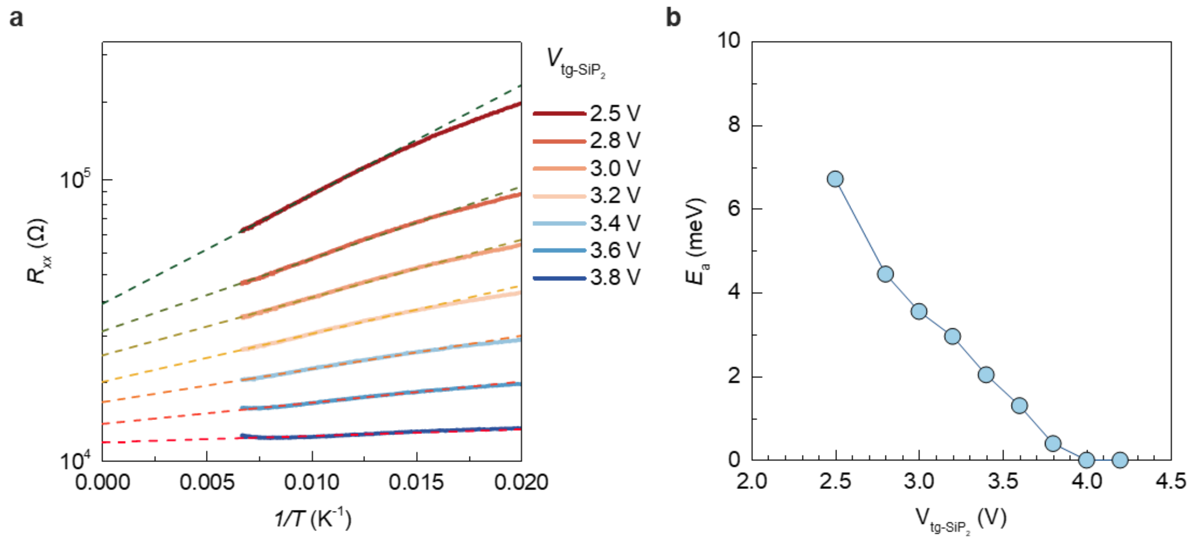

Supplementary Figure 3. Gate-tuned activation energy in 1L-MoS_2/SiP_2 device. a, Temperature dependence of the resistance at varying V_{tg-SiP_2} in an Arrhenius plot. Thermal activation behavior is observed at high temperatures (> 80 K). The dashed lines are fits of $R_{xx} \propto \exp[E_a/(2kT)]$ to the data, from which the activation energy E_a is extracted. **b**, The activation energy E_a as a function of V_{tg-SiP_2}.

4. Estimation of the dielectric constant of SiP_2 via Hall measurements

To experimentally evaluate the dielectric constant ε_r of vdW SiP_2, we measured the 2D carrier density (n_{2D}) as a function of V_{tg-SiP_2} based on the Hall effect measurements, and finally deduced the dielectric constant via the relation $n_{2D} = \varepsilon_0 \varepsilon_r V_{tg-SiP_2}/(e t_{SiP_2})$ (details in Methods), where e is the electron charge, ε_0 is the vacuum permittivity, t_{SiP_2} = 20 nm is the thickness of SiP_2. As shown in Supplementary Fig. 4a, the n_{2D} values remain nearly unchanged before the threshold voltage of 1.7 V and start to increase linearly with the top gate afterward. This value of threshold voltage V_{tg-SiP_2}= 1.7 V is close to its pinch-off voltage estimated from the transfer curve (Fig. 1c in main text). One can see that n_{2D} can be continually modulated from 5×10^9 cm^{-2} to 8×10^{12} cm^{-2} by applying V_{tg-SiP_2} to 5 V (Supplementary Fig. 4a). We can obtain the effective dielectric constant ε_r = 8.1 for SiP_2 by fitting the linear part of the n_{2D}–V_{tg-SiP_2} data using $n_{2D} = \varepsilon_0 \varepsilon_r V_{tg-SiP_2}/(e t_{SiP_2})$. More interestingly, the n_{2D} values can be further modulated by dual-gate control with V_{tg-SiP_2} and V_{bg-SiO_2}, and a maximum n_{2D} close to 10^{13} cm^{-2} can be achieved. An insulator–metal transition (phase diagram shown in Supplementary Fig. 4b) of MoS_2 can be obtained in such a dual-gate configuration, where the critical n_{2D} value for the transition is estimated to be 6.7×10^{12} cm^{-2} (black dashed line) according to previous work[12].

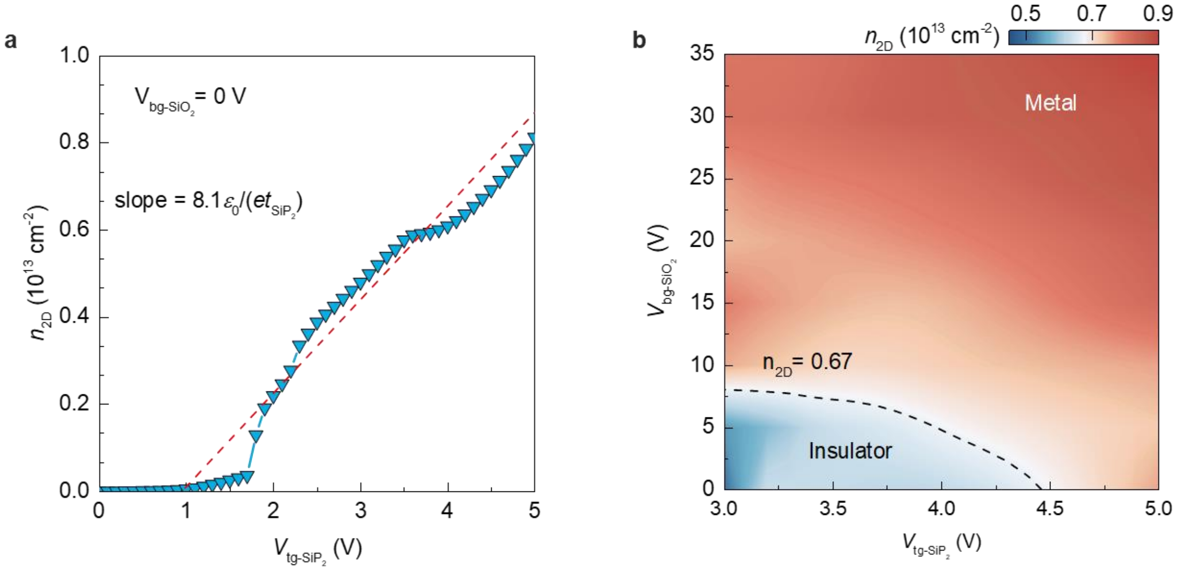

Supplementary Figure 4. Estimation of the dielectric constant of SiP₂ via Hall measurements. a, Two-dimensional carrier density (n_{2D}) as a function of V_{tg-SiP_2}. The dashed line is a linear fitting of data at V_{tg} > 2 V with $n_{2D} = \varepsilon_0 \varepsilon_r V_{tg-SiP_2} / (e t_{SiP_2})$, in which the fitting slope is $8.1\varepsilon_0 / (e t_{SiP_2})$. **b**, Two-dimensional carrier density (n_{2D}) as a function of V_{tg-SiP_2} and V_{bg-SiO_2}. The dashed line highlights n_{2D} = 6.7×10^{12} cm^{-2}.

5. Comparisons of the anisotropic effective mass and the dielectric constant between SiP_2 and other dielectric materials

To quantitatively highlight the difference between the SiP_2 dielectric with non-symmorphic S_2 symmetry (Supplementary Table 3) and the previously investigated isotropic dielectrics (e.g., oxides and h-BN), we compare the calculated effective mass for the conduction and valence bands along different directions and the dielectric constant in SiP_2 with those of other dielectric materials (Supplementary Table 4). We define the "anisotropy ratio" as the ratio of effective mass along the x direction (the [100] direction) and the y direction (the [010] direction). One can see that the effective mass anisotropic ratio in bulk SiP_2 is as large as 15.93 for electron states on the conduction band edge, which is in sharp contrast to those values (less than 2) in other dielectric materials. Furthermore, the calculated dielectric constant of SiP_2 is ~ 8, higher than that of SiO_2 and h-BN, and is comparable to that of some oxides such as Al_2O_3. These facts make SiP_2 a competitive candidate for anisotropic vdW dielectrics.

Supplementary Table 3. Crystal symmetries and symmetry operations of SiP_2 sample[13]

Symmetry	(x, y, z) form	Operation
Identity	x, y, z	$\{1\|0\}$
Glide plane	$\frac{1}{2} - x, \frac{1}{2} + y, \frac{1}{2} + z$	$\left\{M_{100}\middle\|\frac{1}{2}, \frac{1}{2}, \frac{1}{2}\right\}$
Glide plane	$x, y, \frac{1}{2} - z$	$\left\{M_{001}\middle\|0, 0, \frac{1}{2}\right\}$
Glide plane	$\frac{1}{2} + x, \frac{1}{2} - y, z$	$\left\{M_{010}\middle\|\frac{1}{2}, \frac{1}{2}, 0\right\}$
Inversion	$\bar{x}, \bar{y}, \bar{z}$	$\{\bar{1}\|0\}$
Rotation	$\frac{1}{2} + x, \frac{1}{2} - y, \frac{1}{2} - z$	$\left\{2_{100}\middle\|\frac{1}{2}, \frac{1}{2}, \frac{1}{2}\right\}$
Rotation	$\bar{x}, \bar{y}, \frac{1}{2} + z$	$\left\{2_{001}\middle\|0, 0, \frac{1}{2}\right\}$
Rotation	$\frac{1}{2} - x, \frac{1}{2} + y, \bar{z}$	$\left\{2_{010}\middle\|\frac{1}{2}, \frac{1}{2}, 0\right\}$

Supplementary Table 4. Comparison of the anisotropic effective mass and bandgap in dielectric materials

Materials	Space group	Bandgap (eV)	ε_r	Effective mass of electron (m_0)			Effective mass of hole ($-m_0$)			Anisotropy ratio	Refs.
				Γ–K (I1) [100]	Γ–M (I2) [010]	Γ–A (O) [001]	Γ–K (I1) [100]	Γ–M (I2) [010]	Γ–A (O) [001]		
**SiP_2**	$\boldsymbol{Pnma}$	**2.14**	**7.97**	**0.384**	**6.121**	**NA**	**1.555**	**3.683**	**NA**	**15.93**	14
SiO_2	$P3_121$	9	3.9	NA	NA	NA	NA	NA	NA	NA	15-18
SiO_2	$P3_121$	5.68	4.59	0.52	0.52	0.5	9.85	9.85	2.51	1	19-21
α-Al_2O_3	$R\bar{3}c$	8.7	10	0.39	0.39	0.41	6.4	6.2	0.36	1	22
α-Al_2O_3	$R\bar{3}c$	6.48	NA	0.38	0.38	0.38	3.99	3.99	3.99	1	23
α-Al_2O_3	$R\bar{3}c$	8.7~9.4	NA	0.4	0.45	0.16	NA	NA	NA	1.125	24
θ-Al_2O_3	$C2/m$	4.95	NA	0.41	0.41	0.37	0.64	0.64	13.68	1	23
κ-Al_2O_3	$Pna2_1$	5.49	NA	0.37	0.35	0.36	4.9	6.22	0.47	0.95	23
Cubic HfO_2	$Fm\bar{3}m$	3.16	22~25	0.7	0.7	0.7	0.3	3	3	1	15-17
Cubic HfO_2	NA	NA	NA	2	2	2	NA	NA	NA	1	15-17
Tetragonal HfO_2	$P4_2/nmc$	3.75	22~25	0.6	0.6	1.9	0.7	0.7	8.26	1	15-17
Monoclinic HfO_2	$P2_1/c$	3.47	22~25	1.6	1	8.2	2.9	5.5	2.3	0.625	15-17
HfO_2	NA	5.5~6	22~25	NA	NA	NA	NA	NA	NA	NA	16-18
Cubic ZrO_2	$Fm\bar{3}m$	3.1	22~25	0.7	0.7	0.7	0.4	3.3	3.3	1	15-17
Cubic ZrO_2	NA	NA	NA	2.3	2.3	2.3	NA	NA	NA	1	15-17
Tetragonal ZrO_2	$P4_2/nmc$	3.6	22~25	0.9	0.9	2.5	1.6	1.6	2.2	1	15-17
Monoclinic ZrO_2	$P2_1/c$	3.4	22~25	3.2	3.7	3.1	3	3.4	2.9	1.16	15-17
ZrO_2	NA	5.0~7.0	22~25	NA	NA	NA	NA	NA	NA	NA	16-18
TiO_2	NA	3.0~3.5	80~110	NA	NA	NA	NA	NA	NA	NA	18
r-TiO_2	$P4_2/mnm$	NA	NA	NA	NA	NA	0.29	0.16	0.2	NA	25
CaO	$Fm\bar{3}m$	NA	11.95	NA	NA	NA	NA	NA	NA	NA	26
CaO	$Fm\bar{3}m$	3.69	16.47	0.59	0.59	0.59	1.44	1.44	1.44	1	19-21
CaO	$Fm\bar{3}m$	3.55	NA	0.33	0.33	0.33	1.25	1.25	1.25	1	23
MgO	$Fm\bar{3}m$	4.64	10.77	0.35	0.35	0.35	2.34	2.34	2.34	1	19-21
MgO	$Fm\bar{3}m$	NA	9.9	NA	NA	NA	NA	NA	NA	NA	26
MgO	NA	7.58~7.8	NA	0.35	0.35	0.35	0.31	0.35	0.32	1	24
$MgAl_2O_4$	NA	7.8	6.07	0.44	0.44	0.44	NA	NA	NA	1	24
Bi_2SeO_5	$Abm2$	2.88	NA	0.46	0.53	2.3	2.85	4.63	5.49	1.15	19-21
Bi_2SeO_5	$Abm2$	3.9	21	NA	NA	NA	NA	NA	NA	NA	27
hBN	$P6_3$	NA	NA	0.92	1.27	NA	0.82	1.09	NA	1.38	28
1L-hBN	$P6_3$	5.97	3~4	NA	NA	NA	NA	NA	NA	NA	4,29,30

* Anisotropy ratio: the ratio of electron effective mass (m_0) along the [010] direction and [100] direction. NA: not applicable.

6. Berry curvature dipole and circular photo-galvanic effect in symmetry-mismatched heterointerface

The Berry curvature dipole is a crucial topological quantity for characterizing the electron wavefunction in solids and plays a significant role in realizing exotic nonlinear phenomena of quantum materials. In particular, in such symmetry-mismatched van der Waals heterointerfaces, the band structure with interfacial hybridization of atomic orbitals is more sensitive to Berry curvature dipole generation due to symmetry breaking[31]. The Berry curvature dipole should generate an emergent nonlinear optical response (for example, the spin photocurrent) since the optical transition probability is proportional to the value of Berry curvature during the photon-to-electron angular-momentum transfer[31]. To confirm whether such symmetry mismatched heterointerface can generate Berry curvature dipole, we performed the circular photo-galvanic current measurements based on the WSe_2/SiP heterostructure with symmetry breaking similar to that in the SiP_2/TMDC interface. And we indeed observe the circular photo-galvanic effect in such a symmetry-mismatched heterointerface. Supplementary Figure 5 shows the results of the spin photocurrents for a heterointerface under different configurations of incident light. The polarization-dependent photocurrent J can be quantitatively analyzed based on the following relation: $J = C\sin 2\varphi + L_1 \sin 4\varphi + L_2 \cos 4\varphi + D$, where C accounts for the spin photocurrent originating from the Berry curvature dipole, L_1 and L_2 account for the linear photocurrent corresponding to the shift current and thermal photocurrent, and D accounts for a polarization-independent photocurrent. One can see that the circular photo-galvanic current (the component C) can be clearly observed in three different geometries. Such observations of the circular photo-galvanic effect provide experimental evidence that Berry curvature dipole can be generated at the symmetry-mismatched heterointerface. Therefore, the tunable interfacial symmetry in the TMDC/SiP_2 heterostructure can provide a unique platform for investigating symmetry-related interfacial physics and corresponding non-linear phenomena.

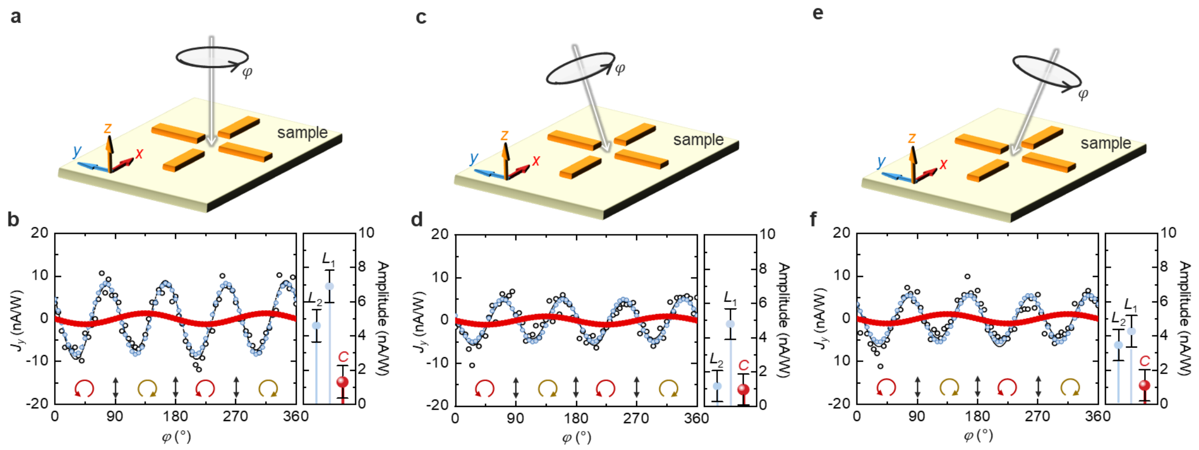

Supplementary Figure 5. Geometric configuration of the spin photocurrent generated from the heterointerface. a–f, Spin photocurrent J_y as a function of φ with λ = 1064 nm, V_{ds} = 0 V, and V_{G} = 0 V. In the schematic figures of the experiment (top), the *x* direction is defined perpendicular to the mirror plane (highlighted in blue) of the interface. The direction of the incident light was set in the *z* direction (**a**–**b**), in the *yz* plane (**c**–**d**) and in the *xz* plane (**e**–**f**). The original data in each panel are shown with black open circles, and the total fitting function in each panel is shown with black lines. The spin photocurrent and linear photocurrent are shown with red and blue circles, respectively. Correspondingly, the fitted parameters of the spin photocurrent component C and linear photocurrent components (L_1 and L_2) are shown in the bar plot on the right side. The error bars are defined by the residual values of error analysis in Fourier series expansion for the original data.

7. Linear dichroic PL spectra generated in 1L-MoS_2 and 1L-WS_2 via symmetry engineering

To understand the symmetry engineering at the interface, we performed polarized PL measurements on 1L-MoS_2/SiP_2 at 77 K. The PL spectra of 1L-MoS_2, SiP_2 (50 nm), and 1L-MoS_2 stacked on SiP_2 are all fitted by the multiple Voigt functions[32] to clarify the peak energies and integrated intensities (Supplementary Fig. 6). For 1L-MoS_2, there are two PL emission peaks located at 1.91 eV and 1.82 eV, of which the higher energy peak (peak 1) corresponds to the exciton of MoS_2 while the lower energy peak (peak 2) corresponds to the defect states[33]. For SiP_2, there are three PL emission peaks, of which the higher energy peak located at 2.06 eV (peak 3) corresponds to the intrinsic exciton of SiP_2, while the other two (peaks 4 and 5) correspond to the defect states[14] in SiP_2. When 1L-MoS_2 is placed on SiP_2, we find a strong lower energy peak on the side of the MoS_2 exciton peak (peak 1) in the PL spectra of the 1L-MoS_2/SiP_2. This exciton state might be related to the defect states[33] in intrinsic 1L-MoS_2 (peak 2), which might be enhanced at the heterointerface.

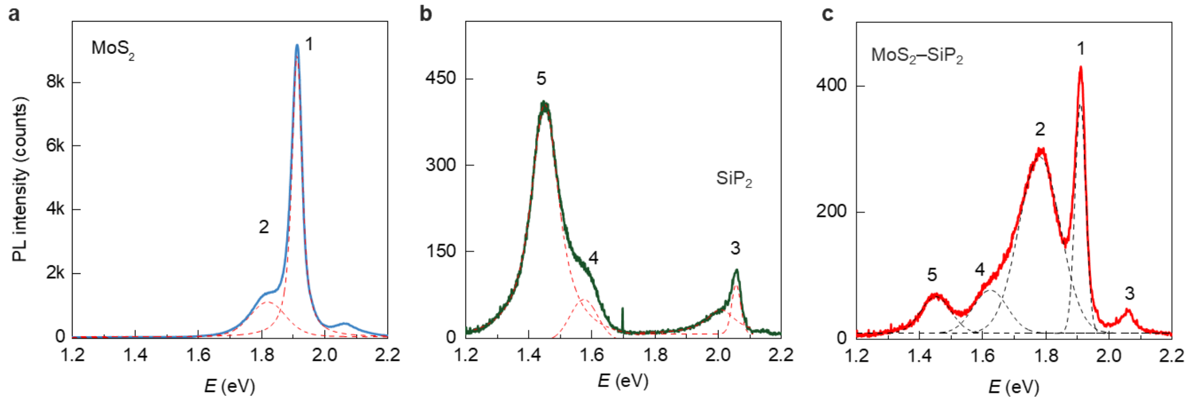

Supplementary Figure 6. Multipeak fitting of PL spectra in the MoS_2/SiP_2 heterostructure. a, PL spectrum of 1L-MoS_2 on SiO_2 substrate at 77 K. Peak 1 corresponds to the exciton of MoS_2, and peak 2 corresponds to the trapped exciton in MoS_2. **b**, PL spectrum of a SiP_2 flake on SiO_2 substrate. Peak 3 corresponds to the exciton of SiP_2, peaks 4 and 5 correspond to the defect states in SiP_2. **c**, PL spectrum of 1L-MoS_2 on SiP_2 dielectric. All PL spectra were fitted with multiple Voigt functions to clarify the peak energies and integrated intensities.

In this study, we mainly focus on the linear polarization properties of intrinsic excitons (peak 1 at 1.91 eV) in 1L-MoS_2 with and without SiP_2 dielectric to understand the symmetry engineering at the heterointerface. In Fig. 2b of the main text, we have demonstrated that the unpolarized exciton state of 1L-MoS_2 at 1.91 eV (peak 1) becomes partially linearly polarized with the polarization along the x direction of the SiP_2 lattice (also the x direction of the heterostructure). More interestingly, we find that the PL emission of the trapped exciton (peak 2) in 1L-MoS_2 also shows similar linear polarization after forming the 1L-MoS_2/SiP_2 interface (Supplementary Fig. 7a–c). Peak 2 of 1L-MoS_2 also becomes partially linearly polarized with the same polarization direction as peak 1. The degree of linear polarization P, which is defined as $P = (I_{\max} - I_{\min})/(I_{\max} + I_{\min})$ is about 0.33, where $I_{\max}$ (or $I_{\min}$) is the maximum (or minimum) PL emission integrated intensity[34].

To further confirm the consistency of the anisotropic PL response at multiple spots of the TMDC/SiP_2 heterostructure, we performed PL mapping on a 1L-MoS_2/SiP_2 heterostructure with detection polarizations along x and y directions (Supplementary Fig. 7d–f). Both peak 1 and peak 2 exhibit a sharp contrast in the PL intensities between x and y detection polarizations. Furthermore, the PL intensity crossing the whole mapping region at a fixed detection angle is modestly uniform, which suggests that the partially linearly polarized exciton states and the symmetry engineering uniformly exist in the heterostructure. Such a result directly shows the great consistency of the anisotropic PL responses at multiple spots of the heterostructure.

To show the universality of the symmetry engineering induced by SiP_2 in other TMDCs, we performed similar polarization-resolved PL measurements in 1L-WS_2/SiP_2. Supplementary Figure 8a shows the PL intensity of 1L-WS_2/SiP_2 as a function of emission photon energy at different detection polarization angles θ at 77 K. Similar to the case of 1L-MoS_2, the nonlinearly-polarized exciton states of 1L-WS_2 (Supplementary Fig. 8c) become partially linearly polarized (Supplementary Fig. 8b). The dramatic changes in the polarization of the PL emission of 1L-WS_2 imply that such symmetry engineering can be extended to other TMDC/SiP_2 systems. To further understand the temperature effect on the observed anisotropic optical properties, we performed polarization-resolved PL measurements in 1L-WS_2/SiP_2 at 300 K (Supplementary Fig. 8d). Similar to the case of 77 K, the nonlinearly-polarized exciton

states of 1L-WS_2 (Supplementary Fig. 8f) at 300 K become partially linearly-polarized with the polarization direction along the *x* direction of the heterostructure (Supplementary Fig. 8e). More specifically, the degree of linear polarization *P* is ~0.15 at 77 K and ~0.14 at 300 K. The linearly-polarized exciton states of 1L-WS_2 show insensitivity to the temperature, indicating the symmetry engineering induced by SiP_2 in TMDCs is robust under different temperatures.

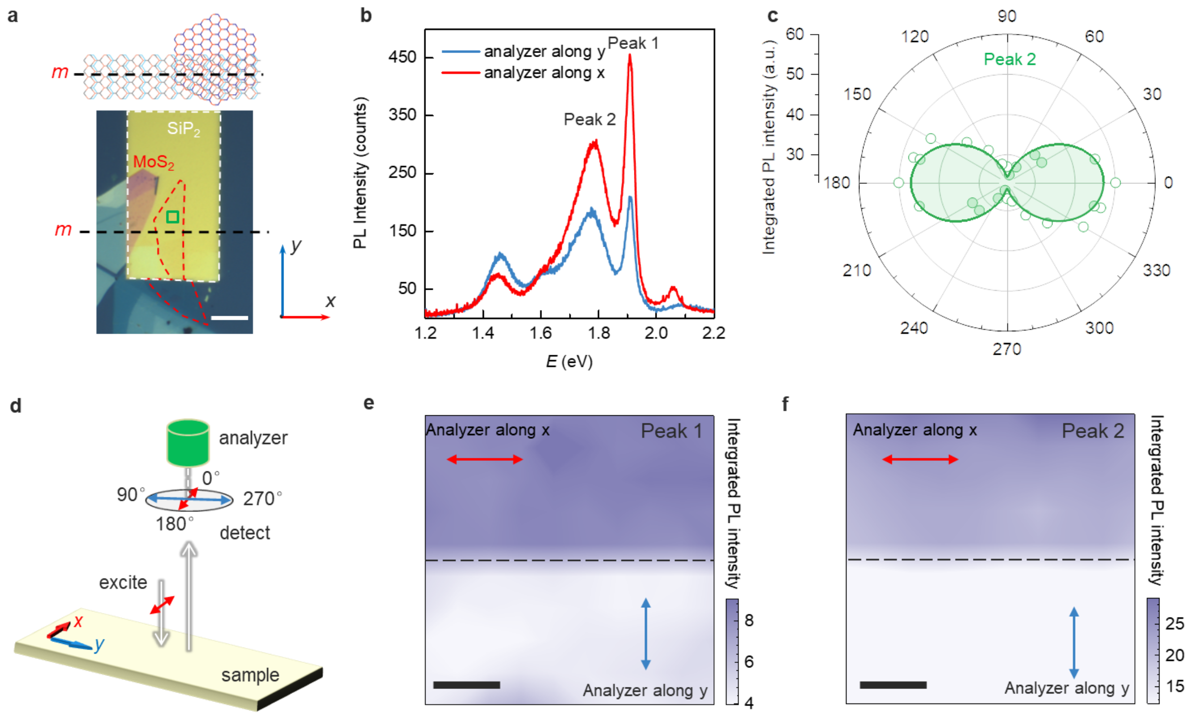

Supplementary Figure 7. Polarization-dependent PL in 1L-MoS_2/SiP_2. a, Schematic (top) and optical image (bottom) of 1L-MoS_2/SiP_2 heterostructure with aligning the armchair direction of MoS_2 and the *x* direction of SiP_2. The red (or white) dashed line outlines the edge of the MoS_2 (or SiP_2) sample. The dashed lines represent the mirror plane. Scale bar is 20 µm. **b**, PL spectra of 1L-MoS_2/SiP_2 with detection polarization along the *x* (red) or *y* (blue) direction. **c**, Polarization-resolved PL integrated intensities of peak 2 in 1L-MoS_2/SiP_2. The solid line is the fitting result using a $\cos^2\theta$ function, in which θ denotes the angle between the detection polarization and the *x* direction. **d**, Schematic of polarized PL measurements geometry. The polarization of the incident laser is fixed along the *x* direction, while the emission light is collected by the analyzer with various polarizations. **e**, **f**, PL mapping of the intensities of peak 1 (**e**) and peak 2 (**f**) in MoS_2 at 77 K. The PL mapping area corresponds to the green square in (**a**). The dashed lines represent the positions where the detection polarization is switched from the *x* direction to the *y* direction. Scale bar is 1 µm.

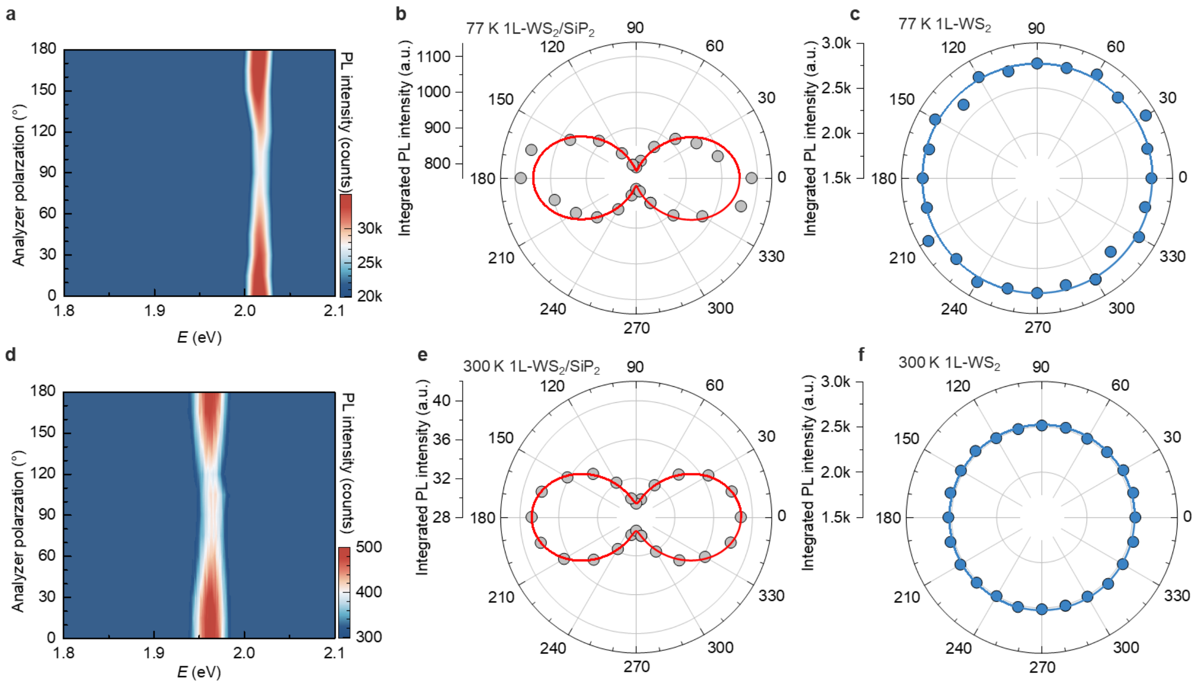

Supplementary Figure 8. Polarization-dependent PL in 1L-WS_2/SiP_2 at 77 K and 300 K. a, Color plot of the PL intensity of 1L-WS_2/SiP_2 as a function of emission photon energy at different detection polarization angles θ at 77 K. **b**, Polarization-resolved PL integrated intensities of the exciton of 1L-WS_2/SiP_2 at 77 K. The solid circle is the experimental data, and the solid line is a fitting result using a $\cos^2\theta$ function. **c**, Polarization-resolved PL integrated intensities of the exciton of 1L-WS_2 on SiO_2 at 77 K. **d**, Color plot of the PL intensity of 1L-WS_2/SiP_2 as a function of emission photon energy at different detection polarization angles θ at 300 K. **e**, Polarization-resolved PL integrated intensities of the exciton of 1L-WS_2/SiP_2 at 300 K. **f**, Polarization-resolved PL integrated intensities of the exciton of 1L-WS_2 on SiO_2 at 300 K.

To investigate whether the trion state in TMDCs can also be modulated by the anisotropic SiP_2 dielectric when forming the SiP_2/TMDC heterostructure, we performed polarization-resolved PL measurements in another 1L-WS_2/SiP_2 heterostructure. As shown in Supplementary Fig. 9a, both the exciton and trion states of 1L-WS_2 are linearly polarized along the x direction of the heterostructure. The degree of linear polarization P in 1L-WS_2 is ~0.16 for excitons and ~0.13 for trions. The dramatic contrast in the polarized PL emission of trion states (polarized state with SiP_2 dielectric and non-polarized state without SiP_2 dielectric) indicates that such symmetry engineering can be extended even to those bound excitonic states.

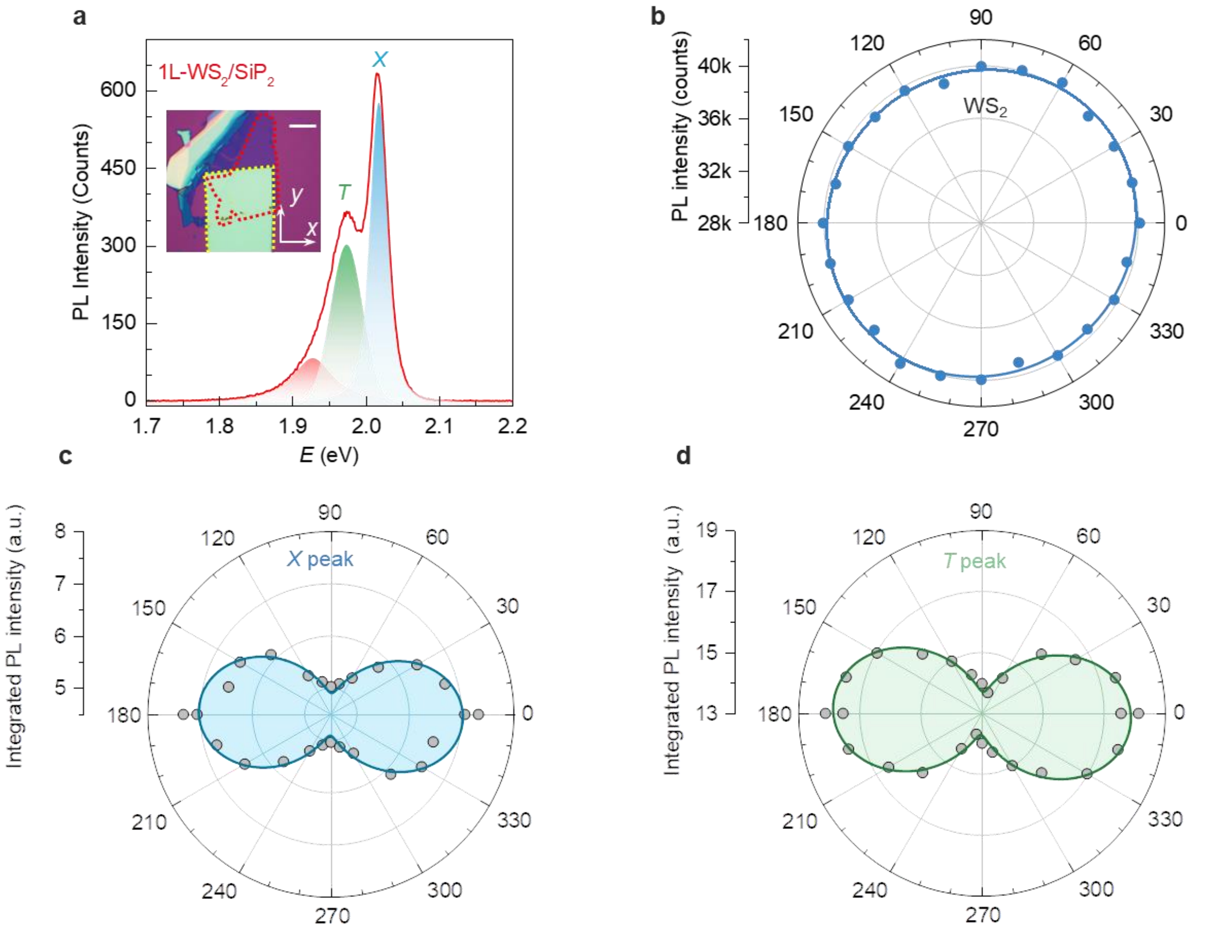

Supplementary Figure 9. Observation of anisotropic trion state in 1L-WS_2/SiP_2. a, PL spectra of 1L-WS_2/SiP_2 with an analyzer along the *x* direction. The blue, green, and red shadows represent the contributions of the exciton (*X*), trion (*T*), and trapped states in 1L-WS_2. Inset: optical image of the 1L-WS_2/SiP_2 heterostructure. The red (or yellow) dashed line represents the WS_2 (or SiP_2) sample. Scale bar is 10 μm. **b,** Polarization-resolved PL integrated intensities of the exciton of 1L-WS_2 on SiO_2. Polarization-resolved PL integrated intensities of excitons (**c**) and trions (**d**) in 1L-WS_2/SiP_2. The solid circle is the experimental data, and the solid line is a fitting result using a $\cos^2\theta$ function.

8. SHG signals and rotational symmetry of 1L-WS_2/SiP_2 and 1L-MoS_2/SiP_2

To confirm symmetry breaking at the interface of monolayer WS_2 with C_3 symmetry and SiP_2 with non-symmorphic S_2 symmetry, we performed polarization-resolved SHG measurements on 1L-WS_2, SiP_2, and 1L-WS_2/SiP_2 heterostructure as shown in Supplementary Fig. 10. The detection polarization is set to be parallel to the excitation polarization. Similar to the 1L-MoS_2 case, the SHG signal of 1L-WS_2 shows a typical sixfold pattern (blue plot), with maxima appearing when the polarizations lie along the armchair directions (Supplementary Fig. 10b), revealing the C_3 symmetry of WS_2 (ref. 35). In contrast, the shape of the six petals becomes asymmetric for the SHG signal of 1L-WS_2/SiP_2 heterostructure (Supplementary Fig. 10a), indicating C_3 symmetry breaking in 1L-WS_2 (refs. 35,36). Such an SHG signal in 1L-WS_2/SiP_2 heterostructure can be well fitted by Equation (2) (more details in the Methods) for SHG signals from strained TMDCs. Note that the SiP_2 substrate has almost no SHG signal due to the existence of inversion symmetry, ensuring that the SHG signal of the 1L-WS_2/SiP_2 is from 1L-WS_2 (Supplementary Fig. 10c).

To investigate the influence of SiP_2 thickness on the anisotropic SHG response on the heterostructure, we performed polarized SHG measurements on 1L-MoS_2/SiP_2 by changing the bottom SiP_2 thickness. As shown in Supplementary Fig. 11, the anisotropic SHG response exists in all the samples with SiP_2 thicknesses ranging from 10 nm to 30 nm. More importantly the anisotropy in the SHG signal changes little with increasing SiP_2 thickness. Such a robust anisotropic SHG response in 1L-MoS_2/SiP_2 and its independence of SiP_2 thickness can be expected since such a phenomenon should only be determined by symmetry breaking at the 1L-MoS_2/SiP_2 heterointerface between the 1L-MoS_2 and the topmost SiP_2 layer and should not be influenced by the bottom bulk SiP_2.

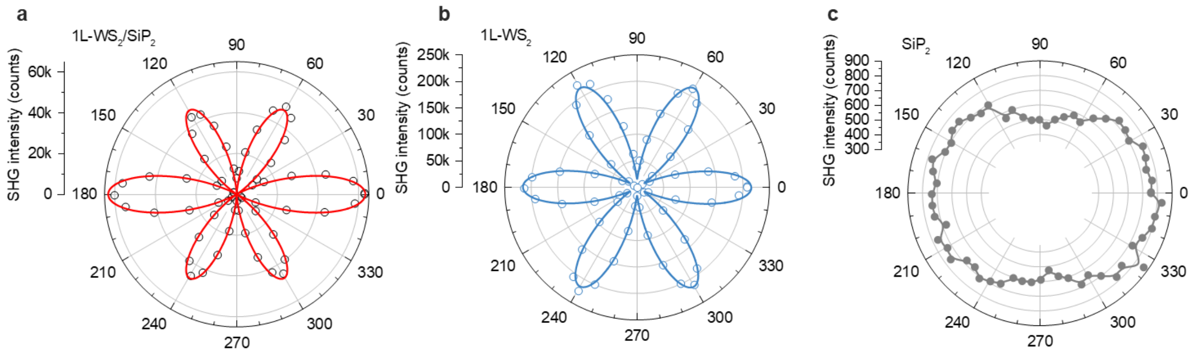

Supplementary Figure 10. SHG signal of 1L-WS_2/SiP_2. a, Polar plot of polarization-resolved SHG intensities of 1L-WS_2 on SiP_2. Black circles represent the measured data, while the red solid line is a fit by Equation (2) in Methods of the main text. **b**, Polar plot of polarization-resolved SHG intensities of 1L-WS_2 on SiO_2. Blue circles represent the measured data, while the blue solid line is a fit by Equation (1). **c**, Polar plot of polarization-resolved SHG intensities of SiP_2.

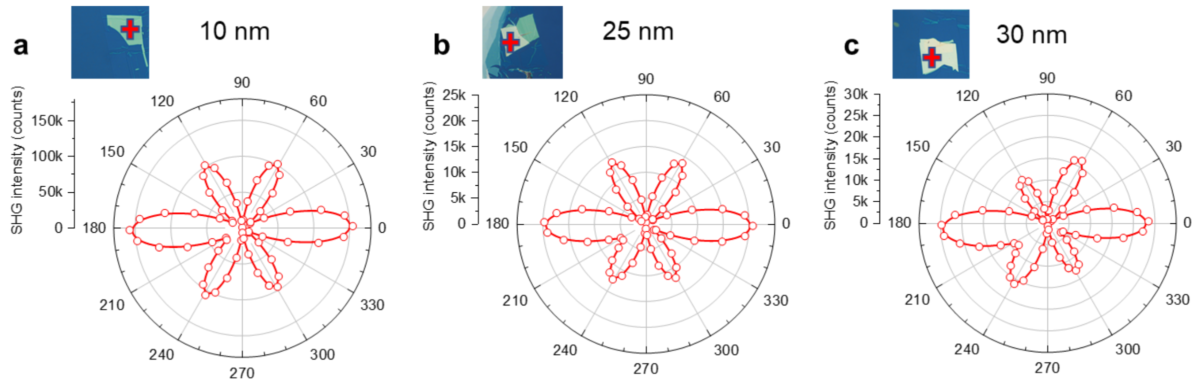

Supplementary Figure 11. Anisotropic SHG responses in 1L-MoS_2/SiP_2 with various SiP_2 thicknesses. a–c, Polar plot of polarization-resolved SHG intensities of 1L-MoS_2/SiP_2 under the parallel configuration (the detection polarization is parallel to the excitation polarization) with SiP_2 thicknesses of 10 nm (**a**), 25 nm (**b**) and 30 nm (**c**). Top inset: optical image of the 1L-MoS_2/SiP_2 heterostructure. The red cross highlights the 1L-MoS_2/SiP_2 region.

9. Twist-angle dependent anisotropic SHG and PL responses in 1L-MoS_2/SiP_2

To investigate the effect of twist angle on the anisotropic properties of the heterostructure, we performed polarization-resolved PL and SHG measurements on various 1L-MoS_2/SiP_2 heterostructures with different twist angles. Specifically, in those 1L-MoS_2/SiP_2 heterostructures with different twist angles (for example 0° and 50° shown in Supplementary Fig. 12), the polarized SHG signals displays the strong anisotropic SHG behavior, which is remarkably different from the six-fold symmetric SHG signals in pristine 1L-MoS_2. Such contrasting behavior before and after stacking SiP_2 to 1L-MoS_2 indicates that the C_3 symmetry of 1L-MoS_2 is broken by the C_2 symmetry of the bottom SiP_2. More importantly, if we compare the polarized SHG signals between those cases with mirror symmetry and without mirror symmetry, one can see that the 0° case with mirror symmetry shows stronger anisotropy in SHG signals than that of the 50° case without mirror symmetry. Such a twist-angle-dependent anisotropic SHG response demonstrates that the mirror symmetry at MoS_2/SiP_2 heterointerface plays key role in generating anisotropic optical responses at the moiré superlattice.

As shown in the polarization-resolved PL results for 1L-MoS_2/SiP_2 heterostructures with various twist angles (Supplementary Figure 13a-f), two important features can be observed as follows: 1) the anisotropic PL responses exist at all twist angles; 2) the polarization degree ($P = \frac{I_{\max}-I_{\min}}{I_{\max}+I_{\min}}$, where $I_{\max}$ and $I_{\min}$ are the maximum and minimum PL intensity) of the PL, which can qualitatively reflect the anisotropic index of PL, shows a strong dependence with the twist angles. For example, the polarization degree P is 0.33 for 0° case and decreases to 0.14 with the twist angle increasing up to 30°. Such a decrease in the anisotropic index of PL might be related to the vanishing of the mirror symmetry with twist angle changing from 0° to 30°. This result indicates that the mirror symmetry of MoS_2/SiP_2 moiré superlattice plays an important role in controlling the magnitude of anisotropic optical responses at the heterointerface.

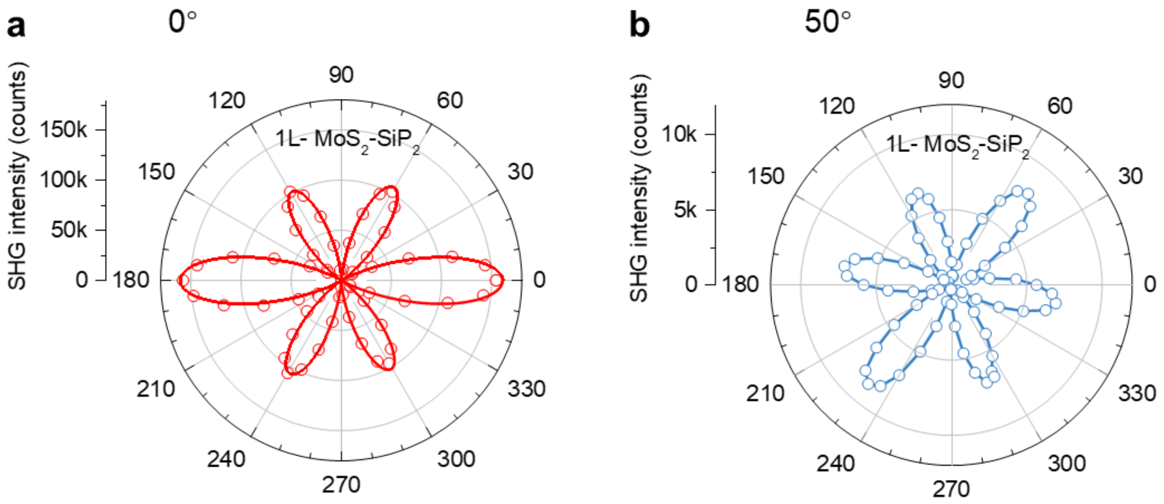

Supplementary Figure 12. Twist-angle-dependent anisotropic SHG response at 1L-MoS_2/SiP_2 heterostructure. a, Polar plot of polarization-resolved SHG intensities of 1L-MoS_2/SiP_2 under the parallel configuration (the detection polarization is parallel to the excitation polarization) with a twist angle of 0° (**a**) and 50° (**b**). The solid lines represent the fitting and the balls represent the experimental data.

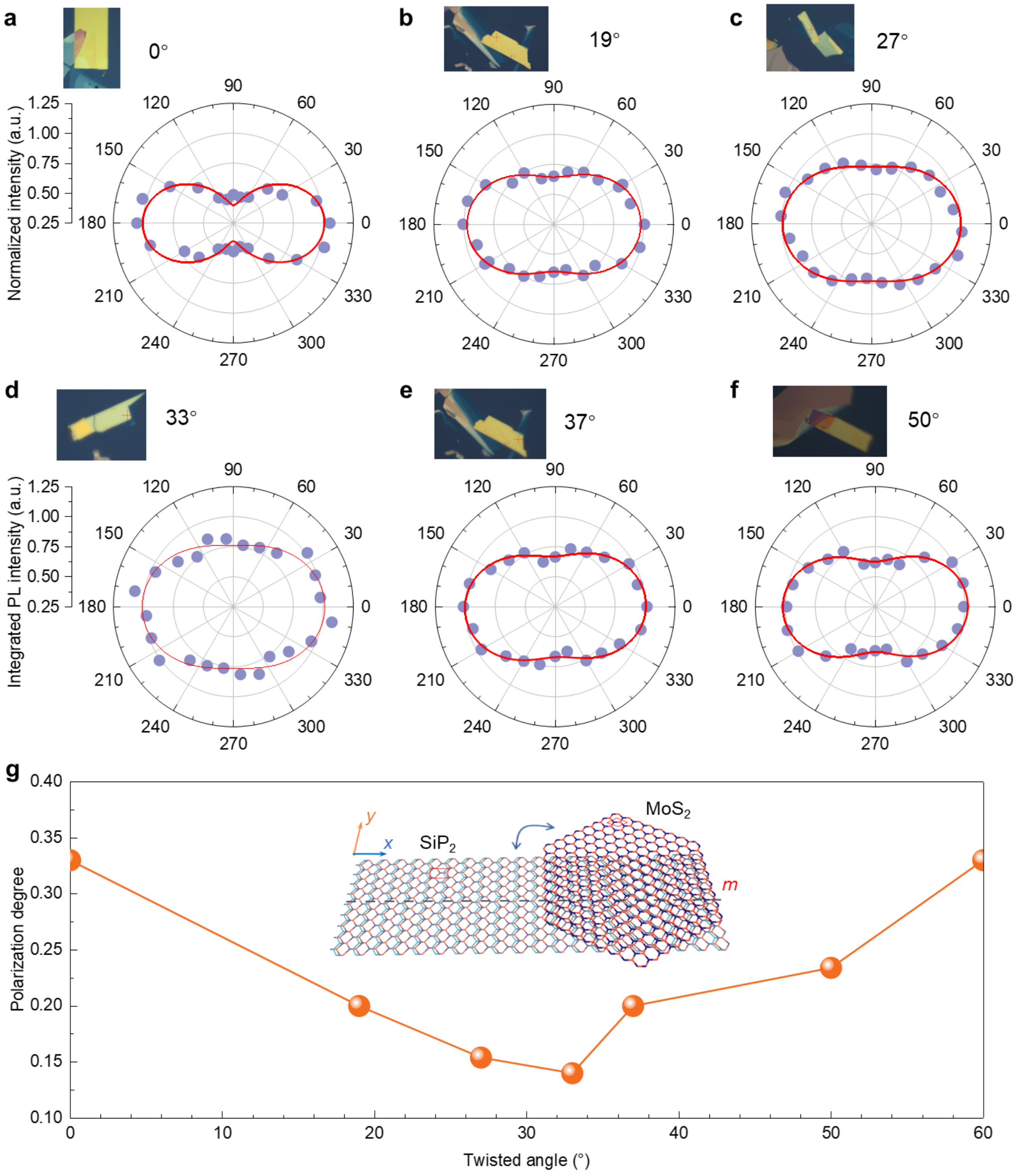

Supplementary Figure 13. Twist-angle-dependent anisotropic PL response at 1L-MoS_2/SiP_2 heterostructure. a–f, Polarization-resolved PL integrated intensities of excitons in 1L-MoS_2/SiP_2 with twisted angle of 0° (**a**), 19° (**b**), 27° (**c**), 33° (**d**), 37° (**e**), 50° (**f**). The solid circle is the experimental data, and the solid line is a fitting result using a $\cos^2\theta$ function. **g**, The polarization degree of the excitons in 1L-MoS_2/SiP_2 as a function of twisted angle.

10. Anisotropic transport properties of 1L-MoS_2 device gated with SiP_2 dielectric

To investigate the anisotropic transfer characteristics of MoS_2 FETs using SiP_2 dielectric, we measured the four-terminal conductance along the x (G_x) and y (G_y) directions of the 1L-MoS_2/SiP_2 heterostructure (Supplementary Fig. 14a,b). Note that the SiP_2/MoS_2 channel used in such measurements is intentionally designed as the square-shape to make the current flow symmetric between x and y directions. We also designed two electrode pads side by side as the source (or drain) electrode, assuming these two big electrode pads can serve as one large electrode for injecting uniform current (Supplementary Fig. 15a,c). Note that such two special designs can make the current uniformly flow through the sample channel and ensure that measurement geometry between x and y directions is equivalent. Specifically, based on the measurement geometry with current flowing along x direction of a typical MoS_2/SiP_2 FET (Supplementary Fig. 15c), we shorted the E1 and E2 as the drain electrode (E3 and E4 as source electrode) to inject a uniform current across the sample, and we measured the conductance between the local electrodes E5 and E6 (or between E7 and E8). Supplementary Fig. 15d shows the conductance between E5 and E6 (or between E7 and E8) as a function of top-gate voltage via SiP_2 dielectric. One can see that the two different channels exhibit almost the same conductance, indicating that the current flow through the sample is relatively uniform. We thus believe such a symmetric measurement geometry can minimize the difference between the measured anisotropic conductance and the anisotropic conductivity. Supplementary Fig. 14c−h shows a comparison of the G_x and G_y values as a function of $V_{\mathrm{tg-SiP_2}}$ at various $V_{\mathrm{bg-SiO_2}}$ (the original data of the G_y/G_x color mapping in Fig. 4g in the main text). One can see that the G_x and G_y values show a large distinction under a relatively small gate voltage, which suggests the anisotropic electrical transport of MoS_2 in the 1L-MoS_2/SiP_2 heterostructure. When increasing $V_{\mathrm{tg-SiP_2}}$ or $V_{\mathrm{bg-SiO_2}}$, the sample becomes more conductive, and the difference between G_x and G_y becomes smaller. Note that the G_x value of the off-state is too small to be detected when $V_{\mathrm{bg-SiO_2}} < 20$ V. Therefore, we use top gate-dependent G_x and G_y at fixed $V_{\mathrm{bg-SiO_2}} = 20$ V as a typical case in the main text to effectively compare the anisotropic conductance.

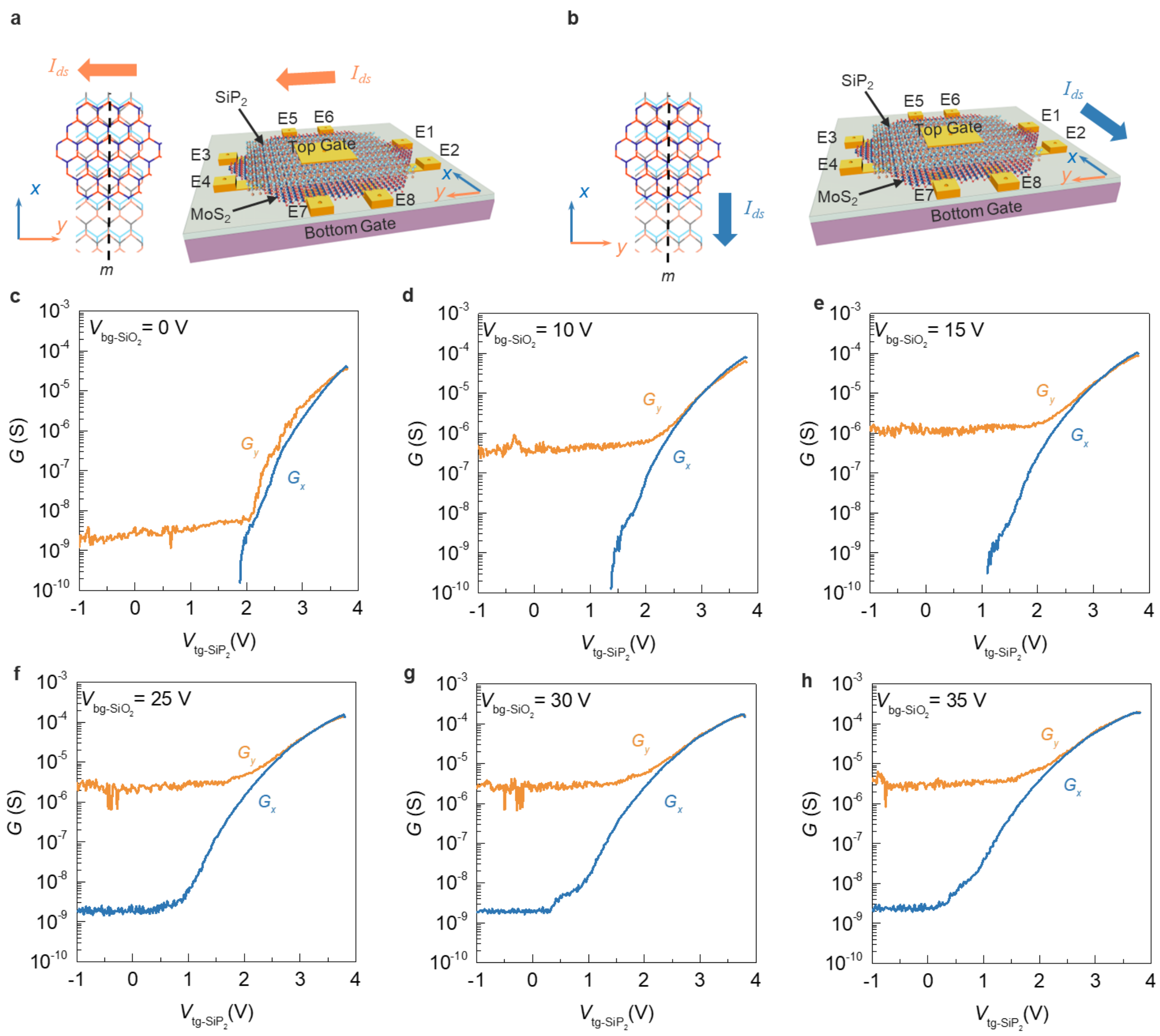

Supplementary Figure 14. Gate-tunable anisotropic transfer characteristics in a 1L-MoS_2/SiP_2 heterostructure. a, Left panel: schematic illustration of the top view of a heterointerface of 1L-MoS_2/SiP_2 for the case where the armchair direction of MoS_2 and x direction of SiP_2 are parallel. The black dashed line represents the mirror plane. Right panel: optical image of the 1L-MoS_2/SiP_2 device with the drain current along the y direction. **b**, Left panel: schematic illustration of the top view of a heterointerface of 1L-MoS_2/SiP_2. Right panel: schematic illustration of the 1L-MoS_2/SiP_2 device with the drain current along the x direction. **c–h**, Comparison of G_x (blue plot) and G_y (orange plot) with sweeping $V_{\mathrm{tg-SiP_2}}$ at fixed $V_{\mathrm{bg-SiO_2}}$= 0, 10, 15, 25, 30 and 35 V. Note that the data obtained at $V_{\mathrm{bg-SiO_2}}$= 20 V are already presented in Fig. 3b in the main text.

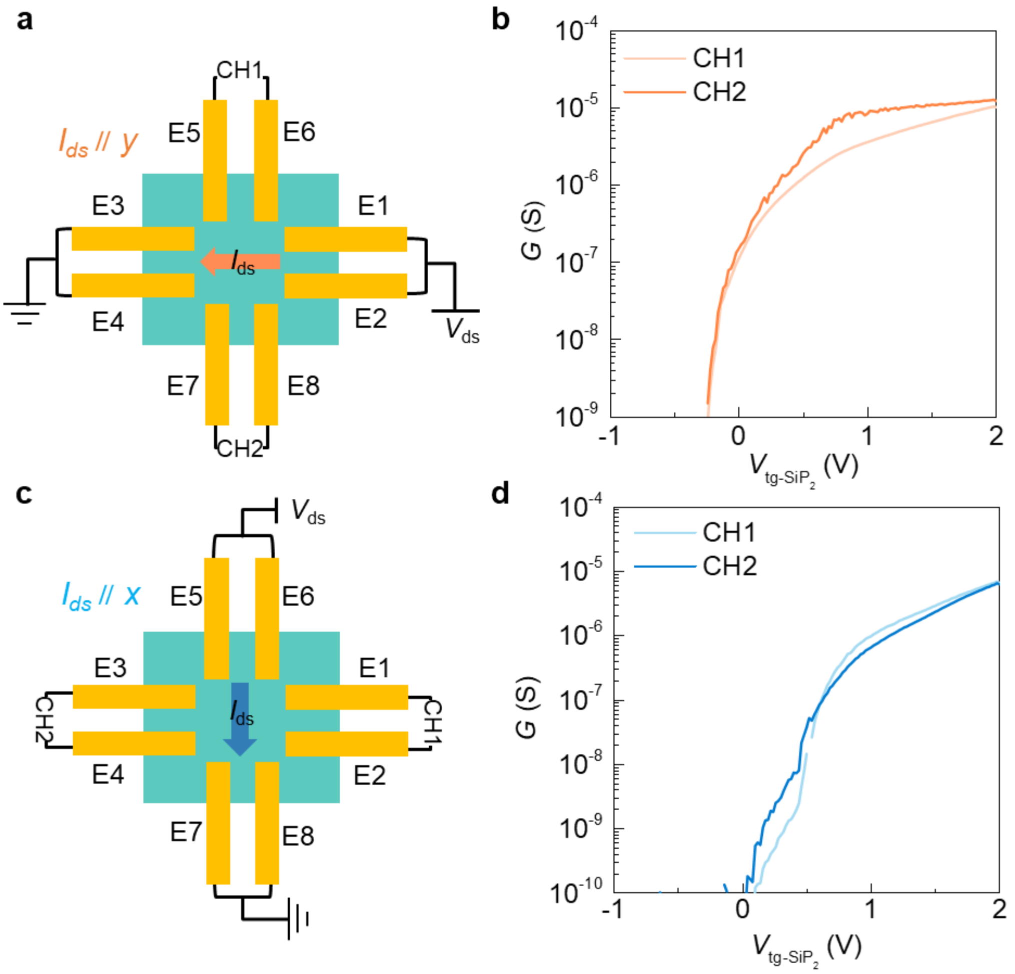

Supplementary Figure 15. Evidence of uniform current through the MoS_2/SiP_2 sample. a, Schematic illustration of our four-wire measurement geometry with current flow along *y* direction. We short the E1 and E2 as the drain electrode while E3 and E4 as the source electrode to inject a relatively uniform current across the sample, and we measure the conductance with the local electrodes E5 and E6 (E7 and E8). **b**, Conductance of channel 1 (E5 and E6) and channel 2 (E7 and E8) as a function of SiP_2 top gate voltage under the measurement geometry in (**a**). **c**, Schematic illustration of our four-wire measurement geometry with current flow along *x* direction. We short the E5 and E6 as the drain electrode while E7 and E8 as the source electrode to inject a relatively uniform current across the sample, and we measure the conductance with the local electrodes E1 and E2 (E3 and E4). **d**, Conductance of channel 1 (E1 and E2) and channel 2 (E3 and E4) as a function of SiP_2 top gate voltage under the measurement geometry in (**c**).

11. Thickness-dependent anisotropic conductance in SiP_2-gated MoS_2 devices

To understand the origin of the anisotropic conductance in MoS_2/SiP_2, we fabricated MoS_2/SiP_2 heterostructures with different thicknesses of MoS_2 and compared their anisotropic conductance ratios. Supplementary Figures 16 and 17 present the corresponding G_y/G_x values as a function of $V_{\mathrm{tg-SiP_2}}$ and $V_{\mathrm{bg-SiO_2}}$ for 5-nm-MoS_2/SiP_2 and 20-nm-MoS_2/SiP_2 devices. Similar to the results of 1L-MoS_2/SiP_2, G_x and G_y also exhibit obvious differences under low gate bias (G_y/G_x >>1) while sharing almost the same values under high gate bias (G_y/G_x ~ 1). However, the maximal anisotropic index G_y/G_x at the off state decreases rapidly with increasing MoS_2 thickness (~ 1000 for 1L-MoS_2, ~ 100 for 5-nm-MoS_2, ~ 5 for 20-nm-MoS_2). This result indicates that the anisotropic conductance behavior is contributed exactly from the interface of the MoS_2/SiP_2 heterostructures (Supplementary Fig. 18) and that the thickness-dependent anisotropic index is the result of the competition between surface and bulk conductance contributions, as discussed in the main text.

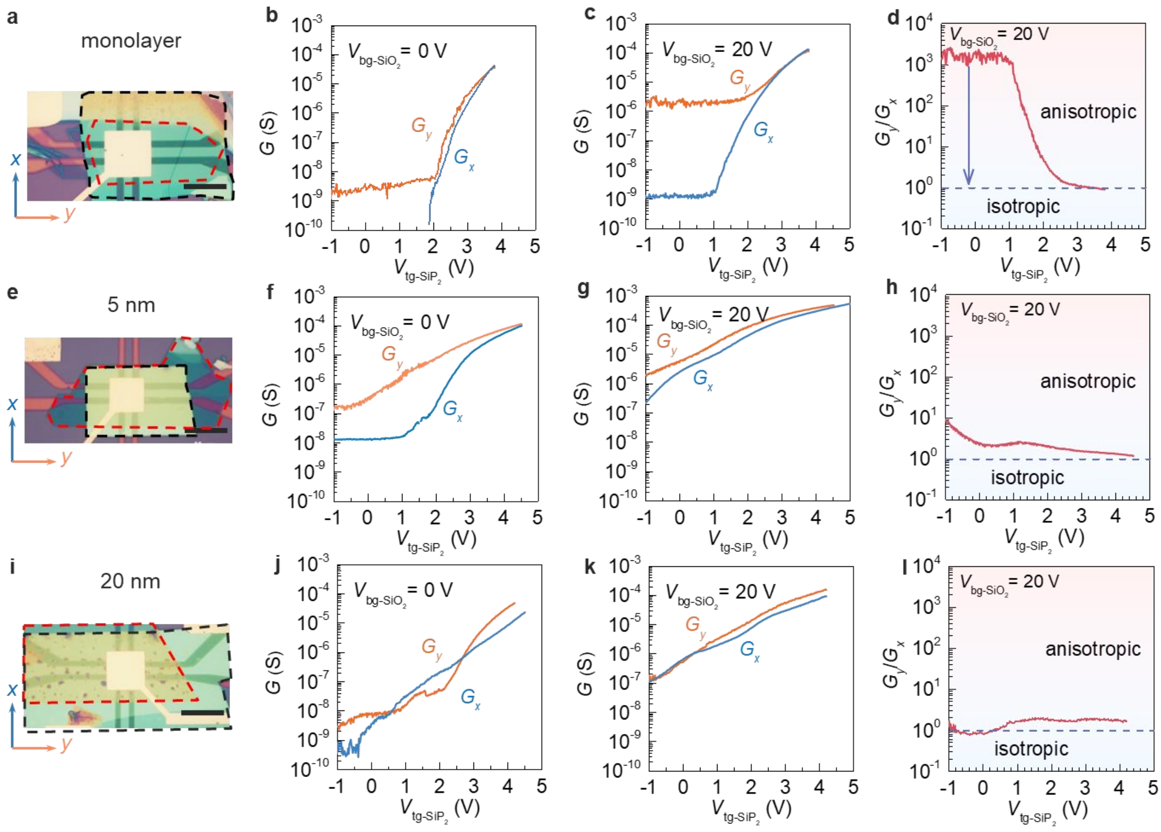

Supplementary Figure 16. Thickness-dependent anisotropic transfer characteristics of the MoS_2/SiP_2 interface. a–d, Optical image of a 1L-MoS_2/SiP_2 device (**a**). The red and black dashed boxes highlight the MoS_2 and SiP_2 samples, respectively. G_x and G_y as a function of V_{tg-SiP_2} at V_{bg-SiO_2}= 0 V (**b**) or 20 V (**c**). G_x and G_y are the sheet conductance of the MoS_2 channel with drain current flowing along and perpendicular to the mirror plane. Anisotropic index "G_y/G_x" as a function of V_{tg-SiP_2} at V_{bg-SiO_2}= 20 V (**d**). The black dashed line highlights G_y/G_x equal to 1. **e–h**, Corresponding results for a 5-nm-MoS_2/SiP_2 device. **i–l**, Corresponding results for a 20-nm-MoS_2/SiP_2 device.

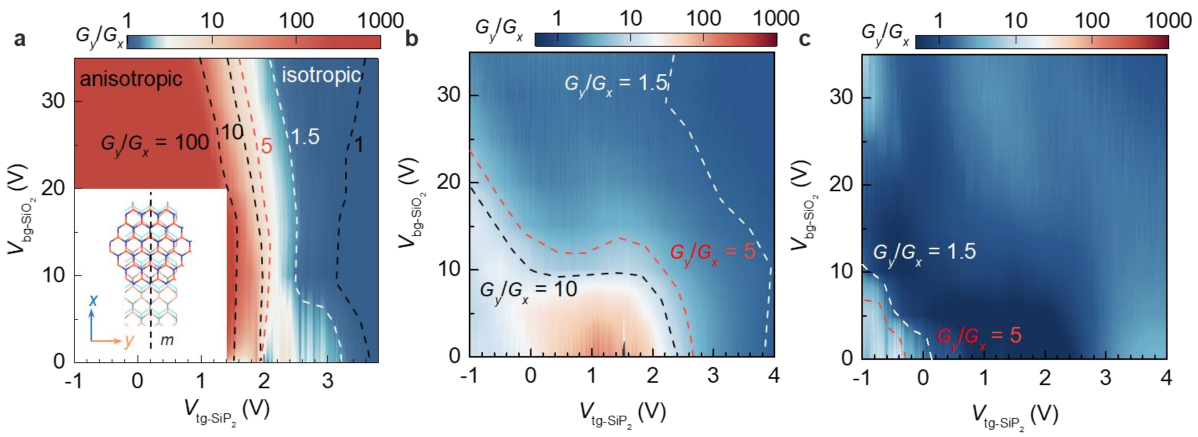

Supplementary Figure 17. Thickness-dependent anisotropic conductance index in MoS_2/ SiP_2 interface. a–c, Color plot of the anisotropic index "G_y/G_x" as a function of V_{tg-SiP_2} and V_{bg-SiO_2} of MoS_2/ SiP_2 device with 1L-MoS_2 (**a**), 5-nm-MoS_2 (**b**) and 20-nm-MoS_2 (**c**). The dashed lines highlight different G_y/G_x values.

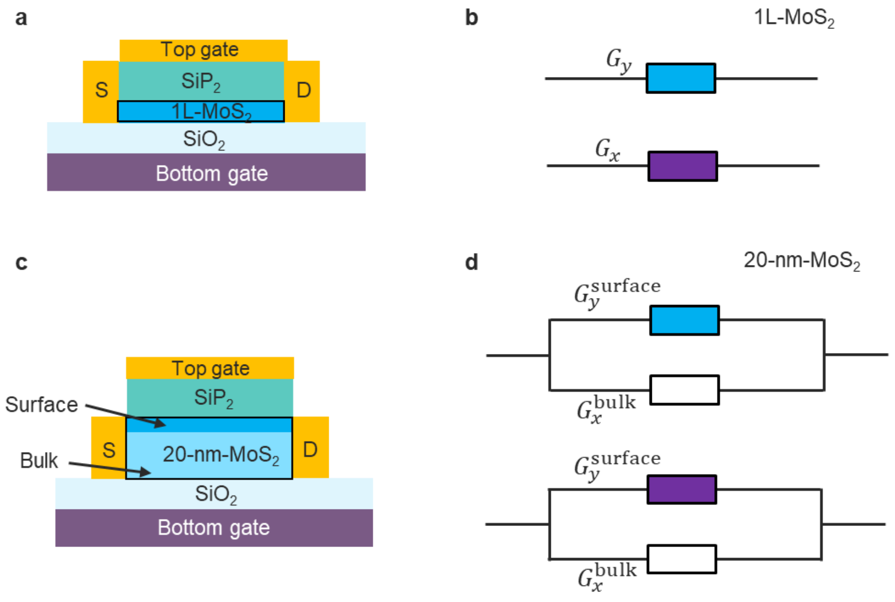

Supplementary Figure 18. Equivalent circuit diagram of monolayer and 20 nm MoS_2 under different measurement geometries. a, Schematic of the SiP_2-gated 1L-MoS_2 device. **b**, Equivalent circuit diagram of monolayer MoS_2 for the measurement geometries with drain current I_{ds} along the x and y directions. All the conductance is contributed by the surface. **c**, Schematic illustration of the SiP_2-gated 20-nm-MoS_2 device. The two arrows highlight the surface and bulk conductance contributions in the MoS_2 channel. **d**, Equivalent circuit diagram of 20-nm-MoS_2 for the measurement geometries with drain current I_{ds} along the x and y directions. Both the surface and the bulk contribute to the conductance.

12. Energy band alignment and carrier density profile with the Poisson-Schrödinger equation

To quantitatively show the electric potential and carrier density distribution along the z direction (out-of-plane direction) in MoS_2 under a dual gate geometry, we solve the Poisson-Schrödinger equations (Supplementary Figs. 19 and 20). Without losing any generality, we neglect the confinement of carriers in the xy plane and only consider the carrier distribution in MoS_2 along the z direction. Therefore, we have the simplified one-dimensional Poisson-Schrödinger equation as follows:

$$\left(-\frac{\hbar^2}{2m^*}\frac{d^2}{dz^2} + U(z)\right)\psi_i(z) = \varepsilon_i \psi_i(z) \tag{7}$$

where ε_i and $\psi_i(z)$ are the eigen energy and eigen wavefunction, respectively, which describe the distribution of carriers along the z direction, m^* is the effective mass of carriers along z direction. Importantly, $U(z)$ is the energy potential of carriers along the z direction, treated as the Hartree potential and described by the Poisson equation[37]

$$\frac{d^2}{dz^2}U(z) = -\frac{e^2}{\varepsilon_0 \varepsilon_{\mathrm{r-MoS_2}}}[n_{\mathrm{D}} - n(z)] \tag{8}$$

where $n(z)$ is the carrier density distribution, n_{D} is the concentration of donor and $\varepsilon_{\mathrm{r-MoS_2}}$ is the relative dielectric constant of MoS_2. Notably, Equations (7) and (8) can be associated with $n(z)$, which determines the potential and can be calculated by the wavefunction $\psi_i(z)$. The boundary conditions are

$$\psi(0) = 0, \qquad \psi(t_{\mathrm{MoS_2}}) = 0 \tag{9}$$

$$\left.\frac{dU(z)}{dz}\right|_{z=0} = \frac{e^2 n_{\mathrm{tg-SiP_2}}}{\varepsilon_0 \varepsilon_{\mathrm{r-SiP_2}}}, \qquad \left.\frac{dU(z)}{dz}\right|_{z=t_{\mathrm{MoS_2}}} = \frac{e^2 n_{\mathrm{bg-SiO_2}}}{\varepsilon_0 \varepsilon_{\mathrm{r-SiO_2}}} \tag{10}$$

where $t_{\mathrm{MoS_2}}$ is the thickness of MoS_2 and $n_{\mathrm{tg-SiP_2}}$ and $n_{\mathrm{bg-SiO_2}}$ are the 2D carrier concentrations accumulated by the top (SiP_2) and bottom (SiO_2) gate.

Based on these analyses, we can consistently calculate the distribution of carrier density $n(z)$,

potential energy $U(z)$, and eigenstate distribution ε_i and $\psi_i(z)$. Taking m^* = 0.49 m_0, ε_{r-MoS_2} = 7.6, t_{MoS_2} = 5 nm, n_D = 1×10^{16} cm^{-3}, ε_{r-SiP_2} = 8, ε_{r-SiO_2} = 4, and temperature T = 2 K, we can obtain the results shown in Supplementary Fig. 20.

Three important points should be addressed here. First, when fixing the effective bottom gate $V_{bg-SiO_2}^{eff}$ ($V_g^{eff} = V_g - V_g^{th}$, where V_g^{th} is the threshold gate voltage) at 0 V and applying a finite $V_{tg-SiP_2}^{eff}$ (Supplementary Fig. 20a), the potential gradient at the top surface creates a planar homogenous electronic system with an inhomogeneous vertical doping profile, where conducting electrons are predominantly confined in the outermost layers of the MoS_2 sample (~ 2 nm) at the SiP_2/MoS_2 interface and show anisotropic transport properties. Second, when fixing $V_{bg-SiO_2}^{eff}$ at 0 V and increasing $V_{tg-SiP_2}^{eff}$ from 0 V to 5 V, the carrier density at the SiP_2/MoS_2 interface can be dramatically increased (Supplementary Fig. 20b). And the moiré potential cannot fully trap the 2D electron gas, and these charge carriers tend to be delocalized on the 2D MoS_2 layer. As a result, in the on-state regime, electrons on the conduction band edge can be treated as a 2D uniform free electron gas, and the conductance turns out to be isotropic. Third, when gradually increasing $V_{bg-SiO_2}^{eff}$ from 0 V to 80 V, carriers accumulate, and a potential well forms at the SiO_2/MoS_2 interface (Supplementary Fig. 20). With such an extra bottom gate, the carrier density at the SiO_2/MoS_2 interface increases, and the sample becomes more conductive than the case with the single top gate. Since the extra carriers induced by the bottom gate are at the SiO_2/MoS_2 interface and not trapped in the moiré potential, the anisotropic properties will fade out with increasing gate voltage, which is consistent with our experimental results in Fig. 3 in the main text and Section 8 in the Supplementary information.

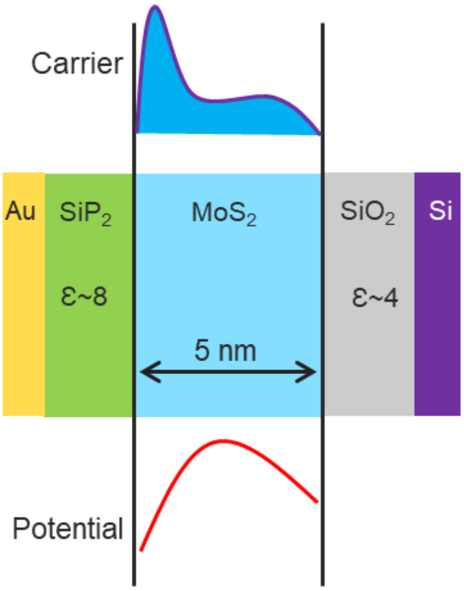

Supplementary Figure 19. Schematic of the potential and carrier density distribution in the MoS_2 layer of a $SiP_2/MoS_2/SiO_2$ dual-gate device. The dielectric constants are 8 for SiP_2 and 4 for SiO_2. The thickness of the MoS_2 layer is 5 nm.

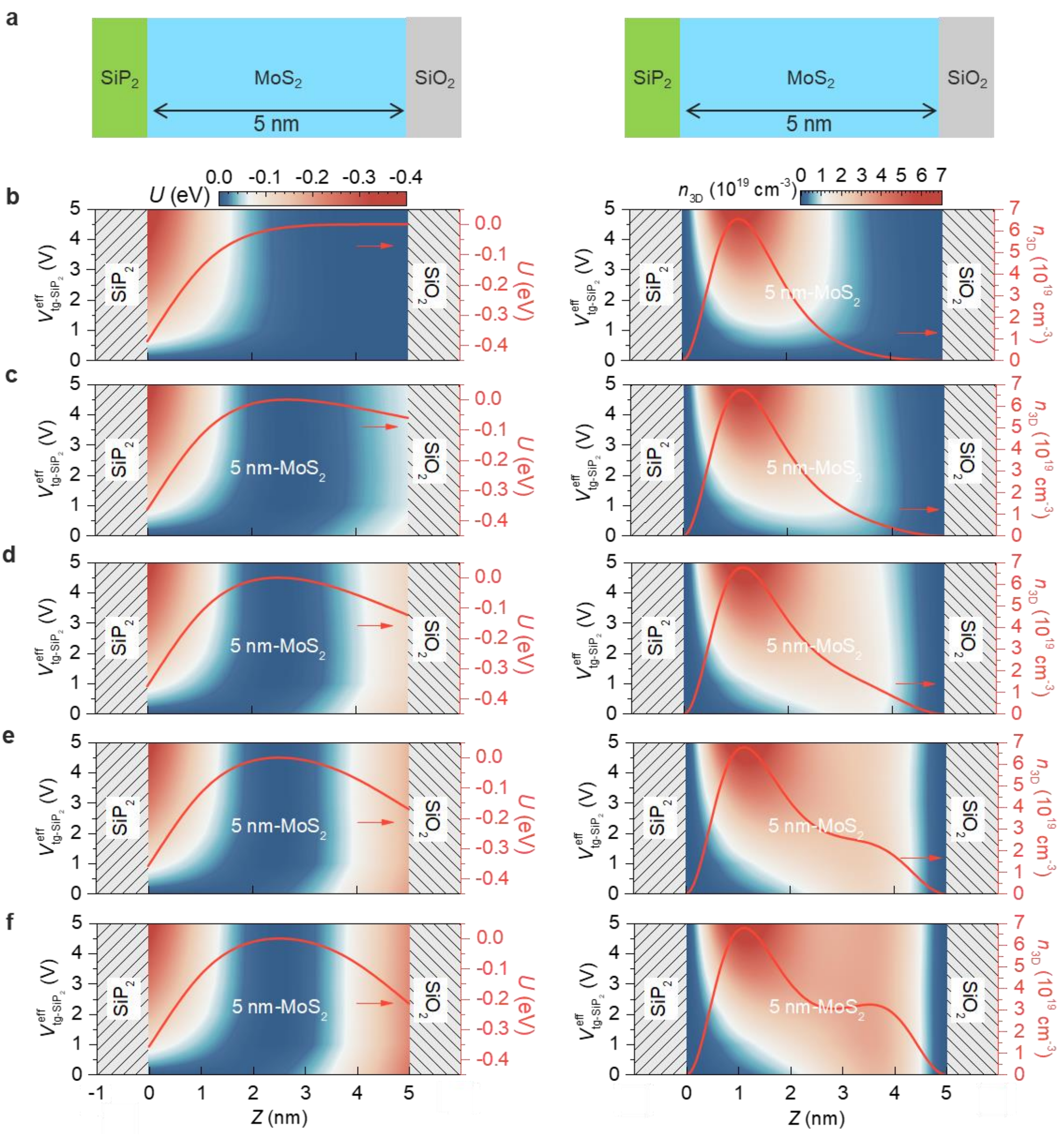

Supplementary Figure 20. Potential and carrier density distribution in the MoS_2 layer simulated by the Poisson-Schrödinger equation. a, Schematic of the $SiP_2/MoS_2/SiO_2$ dual-gate device. The thickness of the MoS_2 layer is 5 nm. **b**, Left panel: potential as a function of $V^{\mathrm{eff}}_{\mathrm{tg-SiP_2}}$ and the distance z at zero $V^{\mathrm{eff}}_{\mathrm{bg-SiO_2}}$. The red solid line is the potential as a function of z at fixed $V^{\mathrm{eff}}_{\mathrm{tg-SiP_2}}$= 5 V. Right panel: three-dimensional carrier density $n_{3\mathrm{D}}$ as a function of $V^{\mathrm{eff}}_{\mathrm{tg-SiP_2}}$ and the distance z at zero $V^{\mathrm{eff}}_{\mathrm{bg-SiO_2}}$. The red solid line is $n_{3\mathrm{D}}$ as a function of z at fixed $V^{\mathrm{eff}}_{\mathrm{tg-SiP_2}}$= 5 V. z = 0 nm represents the interface between the top gate SiP_2 and MoS_2, while z = 5 nm represents the interface between the bottom gate SiO_2 and MoS_2. **c–f**, Similar potential and carrier density distributions in the MoS_2 layer at various $V^{\mathrm{eff}}_{\mathrm{bg-SiO_2}}$ values of 20 V (**c**), 40 V (**d**), 60 V (**e**), and 80 V (**f**).

13. The detailed illustration of the moiré patterns of case-I and case-II

To show the moiré pattern on the heterostructures, we performed first-principles calculations to quantitatively obtain the atomic lattice structures therein. As mentioned in Methods of the main text, the lattice constant *a* (or *b*) of hexagonal 1L-MoS_2 is 3.16 Å; and the lattice constant *a* and *b* of 1L-SiP_2 are 3.44 Å and 10.11 Å, respectively. Due to the large lattice mismatch, we apply a suitable strain to 1L-MoS_2 and 1L-SiP_2, obtaining the commensurate 1L-MoS_2/1L-SiP_2 heterostructure models. Specifically, for 1L-MoS_2, after the formation of the $\sqrt{3} \times 1$ centered rectangular supercell, a compressive strain (–0.008%) is applied along the lattice constant *a*, and the lattice constant *b* is enlarged from 3.47 Å to 3.48 Å. While for 1L-SiP_2, a tensile strain (0.2%) is introduced to the lattice constant *a*, and a compressive strain (–0.6%) is applied to the lattice constant *b*. Finally, the 12 × 11 MoS_2 centered rectangular lattice and 11 × 6 SiP_2 lattice are served as the bricks to form the heterostructure models, where the lattice constants *a* and *b* of the commensurate heterostructure models are 37.917 Å and 60.282 Å, respectively. The magnitude of the corresponding reciprocal lattice vector $\boldsymbol{a^*}$ is 0.166 $Å^{-1}$, while that of the corresponding reciprocal lattice vector $\boldsymbol{b^*}$ is 0.104 $Å^{-1}$, which defines the size of the moiré Brillouin zone. Note that there are two distinguishable commensurate heterostructures with mirror symmetry, marked as case-I and case-II (details are discussed below), as shown in Supplementary Fig. 21a, b. Compared with the moiré superlattice of case-I, the moiré superlattice of case-II can be obtained by rotating the MoS_2 layer by 180° along the *z* direction while fixing the SiP_2 layer. Owing to the lattice mismatch between 1L-MoS_2 and 1L-SiP_2, an evident moiré pattern including AA, AB, and BA stackings (labeled by the red, green, and blue rectangles in Supplementary Fig. 21a, b) inside, can be observed from the top views. Explicit illustrations of the three various stacking configurations in the moiré patterns of case-I and case-II are shown in Supplementary Fig. 21c, d. And the differences between these two commensurate moiré superlattices from the side views are presented in Supplementary Fig. 21e.

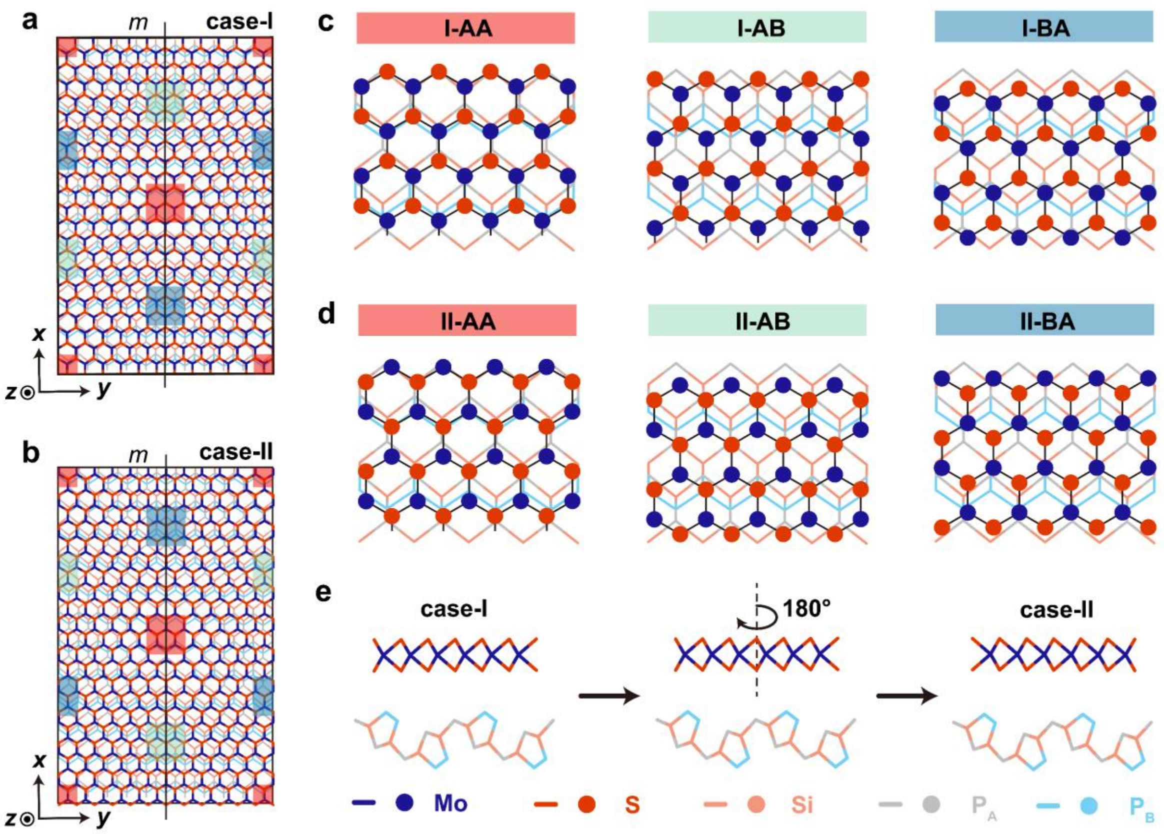

Supplementary Figure 21. Illustrations of the moiré patterns of case-I and case-II. a–b, The moiré patterns of case-I and case-II, respectively. **c**, The top views of AA, AB, and BA stacking configurations in the moiré patterns of case-I, labeled as I-AA, I-AB, and I-BA, respectively. **d**, The top views of AA, AB, and BA stacked structures in the moiré patterns of case-II, marked as II-AA, II-AB, and II-BA, respectively. **e**, Side views of the moiré superlattices of case-I and case-II.

14. Band alignment of the unstrained 1L-MoS_2/SiP_2 heterostructure

In this section, we use DFT calculations to get the band alignment for the unstrained 1L-MoS_2 and 1L-SiP_2, the detailed lattice constant parameters can be found in the Methods section. The work function (Φ) is defined as the energy difference between the valence band maximum (VBM) and the vacuum level (E_{vac}). As listed in Supplementary Table 5, under the PBE functional, the bandgap and the work function of the 1L-MoS_2 are 1.67 eV and 5.85 eV, while those of the 1L-SiP_2 are 1.51 eV and 5.66 eV. These values are consistent with previous studies[38-41]. Note that the bandgaps of the semiconductors are normally underestimated by the LDA or GGA functional. Thus, we correct the electronic structures of the 1L-MoS_2 and 1L-SiP_2 with the HSE06 functional to obtain the band alignment.

Since the 1L-MoS_2 is stacked on the SiP_2 thin flakes instead of the SiP_2 monolayer in the experiments, we first investigate the thickness dependence of the work function of SiP_2 with the PBE functional. As shown in Supplementary Fig. 22a, the work function of SiP_2 decreases with increasing thickness, and saturates when the thickness increases to six layers. Therefore, we treat the work function of the SiP_2 thin flakes as that with the thickness of 6-layer.

Although the values of the work function of SiP_2 multilayers will be different between the results under the PBE functional and HSE06 functional, the thickness dependence of the work function of SiP_2 should be the same. The work function difference between SiP_2 monolayer and one with the thickness of six-layer is defined as Δ (labeled in Supplementary Fig. 22a). Thus, approximately the work function of the SiP_2 thin flake is estimated by subtracting the absolute value of Δ from the work function of 1L-SiP_2 under the HSE06 functional. Moreover, the bandgap of 2.14 eV of the bulk SiP_2 sample[14] is approximately treated as the bandgap of the SiP_2 thin flake. The corrected band alignment between the 1L-MoS_2 and SiP_2 thin film is shown in Supplementary Fig. 22b. One can see that the 1L-MoS_2/SiP_2 heterostructure belongs to type II heterostructure, the conduction band edge is mainly contributed by 1L-MoS_2, which is consistence with results from optical and transport measurements.

Importantly, as shown in Supplementary Fig. 22c, the band alignment of the 1L-MoS_2/1L-SiP_2 heterostructure is similar to that of the 1L-MoS_2/SiP_2 heterostructure, which means that the

electronic properties of the 1L-MoS_2/SiP_2 heterostructure (such as transport behavior of electrons on the conduction band edge) can be approximated by the corresponding electronic properties of the 1L-MoS_2/1L-SiP_2 heterostructure. Therefore, in DFT calculations, we only focus on the heterostructure models formed by 1L-MoS_2 and 1L-SiP_2.

To experimentally determine the band alignment of the 1L-MoS_2/SiP_2 heterostructure, we performed additional Kelvin probe force microscopy (KPFM) measurements. One can see in Supplementary Fig. 23c that the work functions of the monolayer MoS_2 and SiP_2 flake are estimated to be approximately 4.61 eV and 4.96 eV, respectively. Based on the fact that SiP_2 (1L-MoS_2) is a semiconductor with a band gap of 2.25 eV (2.11 eV) according to our calculations and the assumption that their Fermi level is sitting at the middle of the band gap, we confirm that the energy levels of the conduction band minimum and the valence band maximum of SiP_2 are sitting at −3.48 eV and −5.73 eV, respectively (as indicated by the solid lines in Supplementary Fig. 23f), while the conduction band minimum and the valence band maximum of 1L-MoS_2 are sitting at −3.90 eV and −6.01 eV, respectively (as indicated by the solid lines in Supplementary Fig. 23f). Therefore, we can verify that the 1L-MoS_2/SiP_2 heterostructure forms a type-II band alignment. Such a result is well-consistent with our DFT calculated results. Importantly, the morphological characterizations of the heterostructures via KPFM have been proven to be a powerful tool to directly observe the moiré superlattice therein. From this perspective, the morphological characterizations of anisotropic moiré superlattices at the 1L-MoS_2/SiP_2 interface with identified twist angles via KPFM can help understand the moiré physics therein and can be a good research topic in the near future.

Supplementary Table 5. The values of bandgap (E_g) and work function (Φ) of the 1L-MoS_2 and 1L-SiP_2 with the PBE and HSE06 functional, and results from previous studies

	E_g (eV)	E_g^{corr} (eV)	Φ (eV)	Φ^{corr} (eV)	Refs.
1L-MoS_2	1.67	2.11	5.85	6.25	This work
MoS_2	1.59	2.02	NA	NA	38
MoS_2	1.58	2.40	5.91	6.42	39
MoS_2	1.80	NA	NA	NA	40
1L-SiP_2	1.51	2.28	5.66	6.16	This work
SiP_2	1.45	2.25	NA	6.01	41

* E_g and Φ : values are obtained from the calculations under the PBE functionals. E_g^{corr} and Φ^{corr}: values are calculated by the HSE06 method or the GW method. NA: not applicable.

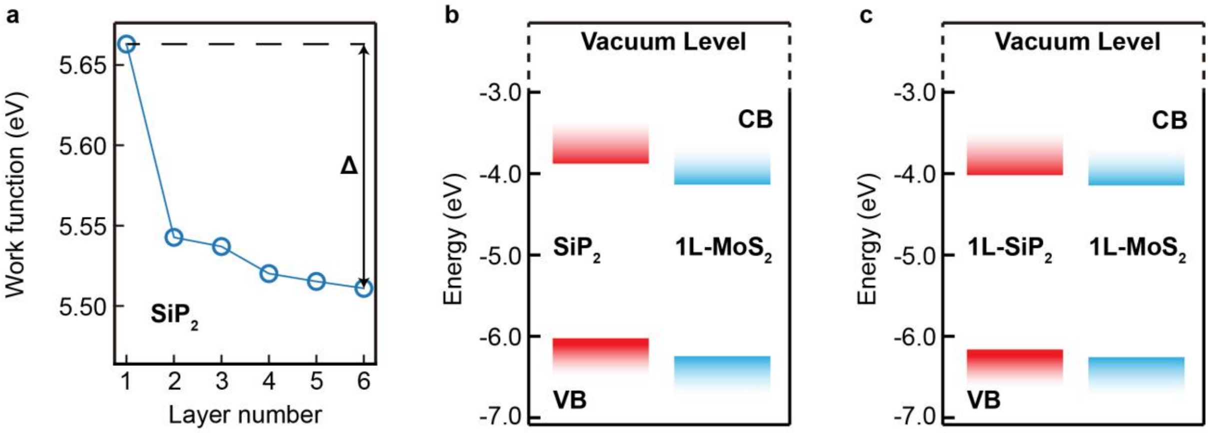

Supplementary Figure 22. Illustrations of work function of SiP_2 and the band alignment. a, The thickness dependence of the work function of SiP_2 with the PBE functional. **b**, The band alignment of the 1L-MoS_2/SiP_2 heterostructure with the HSE06 functional. **c**, The band alignment of the 1L-MoS_2/1L-SiP_2 heterostructure with the HSE06 functional. The vacuum level is set as zero.

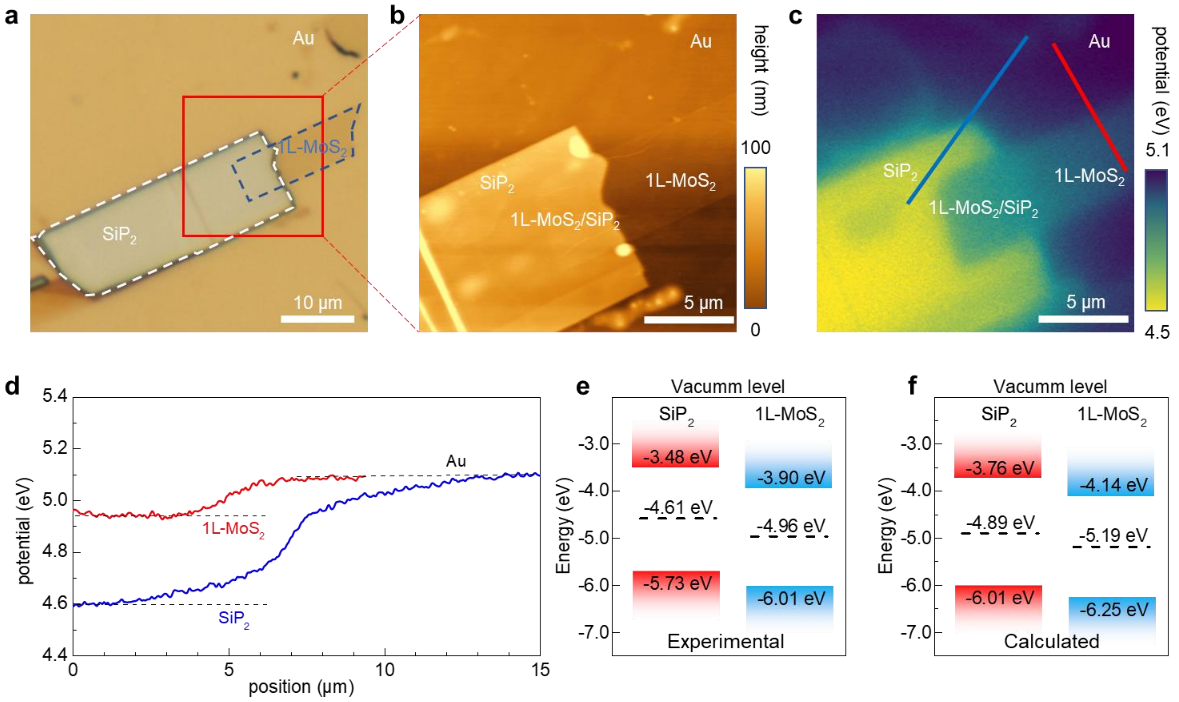

Supplementary Figure 23. Confirmation of the work functions and band alignment of the 1L-MoS_2/SiP_2 heterostructure based on KPFM measurements. **a**, Optical microscopy image of the 1L-MoS_2/SiP_2 heterostructure on Au film. The regions of monolayer 1L-MoS_2 and SiP_2 are highlighted by blue and white dashed polygons. The red solid square indicates the region for the AFM and KPFM measurements. **b**, Atomic force microscope image of the 1L-MoS_2/SiP_2 heterostructure. The regions of the monolayer MoS_2, Au film, 1L-MoS_2/SiP_2 heterostructure and SiP_2 flake are labeled. **c**, KPFM imaging of the 1L-MoS_2/SiP_2 heterostructure. The red and blue lines indicate the profiles from 1L-MoS_2 to Au film and from SiP_2 to Au film. **d**, The profiles from 1L-MoS_2 to Au film (red) and from SiP_2 to Au film (blue) in the KPFM measurement. **e**, Schematic figure for type-II band alignment between monolayer MoS_2 and SiP_2, confirmed from the above KPFM measurements. The dashed lines indicate the energy levels of the Fermi level. The experimental estimations for the conduction band minima and the valence band maxima of 1L-MoS_2 and SiP_2 are labeled. **g**, Schematic figure for type-II band alignment between monolayer MoS_2 and SiP_2 according to the energy levels obtained from our first-principles calculations.

15. The construction of the heterostructure models used in the DFT calculations

The strain is intentionally applied to lower the lattice mismatch between the unstrained 1L-MoS_2 and unstrained 1L-SiP_2, which allow us to build suitable supercells and make direct DFT calculations possible. To confirm that strained 1L-MoS_2 and 1L-SiP_2 can replace the pristine MoS_2 and SiP_2 to build heterostructure models, we comprehensively compare the band structures of the unstrained and strained samples. To avoid confusion, in the following discussions, for 1L-MoS_2, both unstrained and strained $2\sqrt{3} \times 1$ supercells are used in the calculations, instead of the primitive hexagonal unit cells.

On one hand, considering results from the PBE functional, the unstrained 1L-MoS_2 is a semiconductor with a direct band gap of 1.67 eV. Its valence band maximum (VBM) and conduction band minimum (CBM) are located at the K point in the first Brillouin zone (BZ, see details in Supplementary Fig. 24c). For the strained 1L-MoS_2, the calculated bandgap is 1.63 eV that is similar with that in unstrained case. Although the strained 1L-MoS_2 becomes an indirect bandgap semiconductor, the strain induced momentum shift for the band edges is quite small and the band structures of unstrained and strained cases are similar (Supplementary Fig. 24a–b). Supplementary Table 6 shows values of the bandgap and work functions of the unstrained and strained 1L-MoS_2 and 1L-SiP_2 with the PBE functional and the HSE06 functional. One can see that the bandgaps of the unstrained and strained 1L-MoS_2 and 1L-SiP_2 are similar with the same exchange-correlation functional.

On the other hand, with the HSE06 functional, the band alignment of strained 1L-MoS_2/1L-SiP_2 heterostructure is illustrated in Supplementary Fig. 24h. Compared with the band alignment of the unstrained 1L-MoS_2/1L-SiP_2 heterostructure (Supplementary Fig. 24g), one can see that the conduction band offset is not influenced by the strain and the conduction band edge is also contributed by the MoS_2 layer. Although the valence band offset is reversed by applied artificial strain, the preserved conduction band offset enables us to investigate the transport behavior of the electrons correctly with the strained slab models.

In conclusion, although the inevitable influence on electronic structures is introduced when we apply the strain artificially, our model with strained 1L-MoS_2 and 1L-SiP_2 still can be used to

replace the unstrained 1L-MoS_2 and 1L-SiP_2 to build heterostructures that can be afforded for DFT calculations. Although the PBE functional fails to give the correct conduction band offset (Supplementary Fig. 24i), other electronic properties of the semiconductor heterostructure, such as the charge density, electronic state hybridization, and so on, still can be correctly described by it. Considering the calculations of the strained heterostructure with HSE06 functional are so expensive, all the following calculations are performed with PBE functional.

Supplementary Table 6. The values of bandgap (E_g) and work function (Φ) of the unstrained and strained 1L-MoS_2 and 1L-SiP_2 with the PBE and HSE06 functional

	E_g (eV)	E_g^{corr} (eV)	Φ (eV)	Φ^{corr} (eV)
Unstrained 1L-MoS_2	1.67	2.11	5.85	6.25
Strained 1L-MoS_2	1.63	2.06	5.83	6.23
Unstrained 1L-SiP_2	1.51	2.28	5.66	6.16
Strained 1L-SiP_2	1.50	2.29	5.88	6.40

* E_g and Φ: values are obtained from the calculations under the PBE functional. E_g^{corr} and Φ^{corr}: values are calculated with the HSE06 functional.

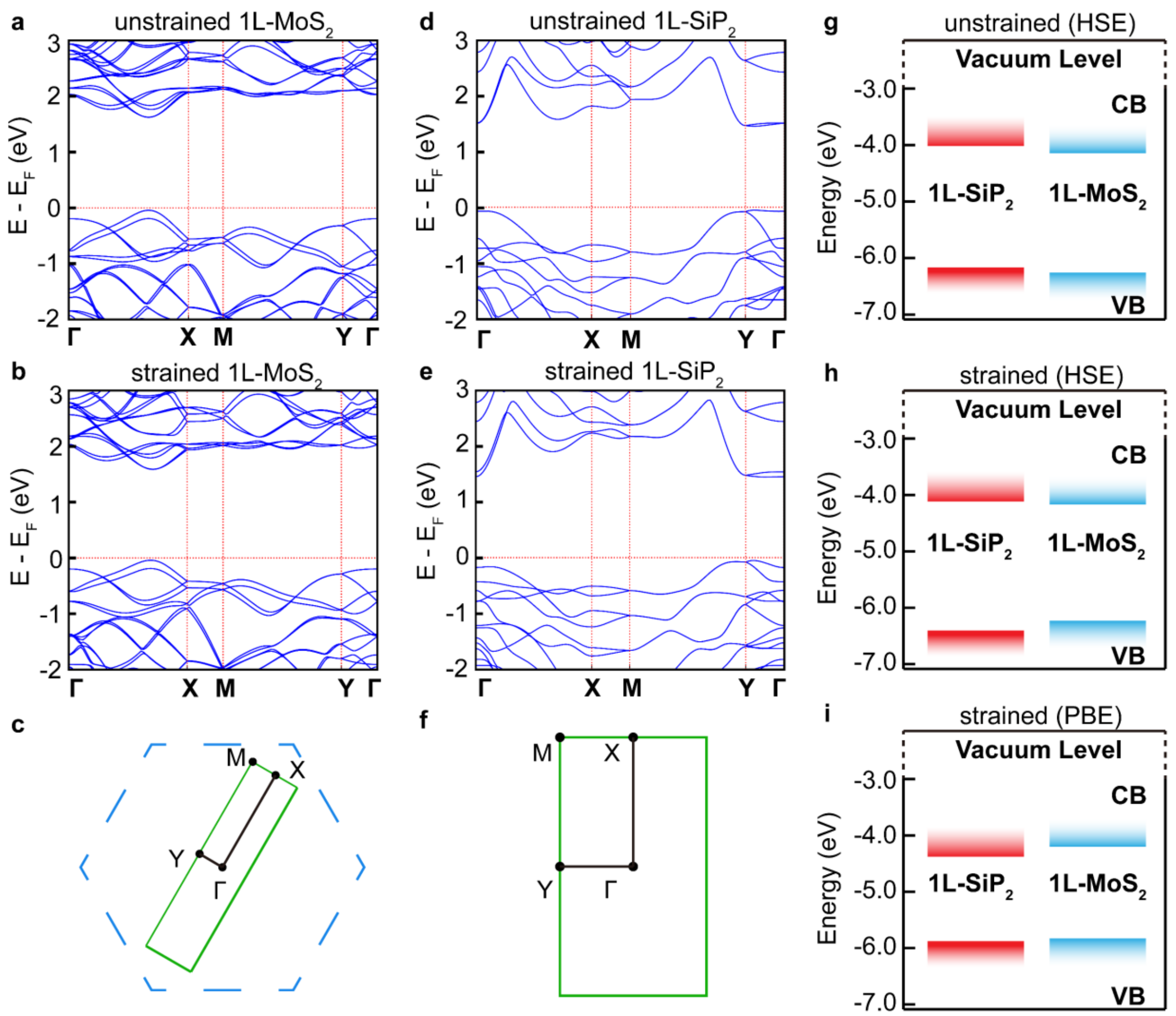

Supplementary Figure 24. The electronic structures of the unstrained and strained 1L-MoS_2 and 1L-SiP_2. a–b, The band structures of the unstrained and strained 1L-MoS_2 unit cells with the PBE functional. **c**, The first Brillouin zones of the 1L-MoS_2 rectangular unit cell (green) and the 1L-MoS_2 primitive hexagonal cell (blue). **d–e**, The band structures of the 1L-SiP_2 unit cells with the PBE functional. **f**, The first Brillouin zone of the 1L-SiP_2 unit cell. **g–h**, The band alignments of the unstrained and strained 1L-MoS_2/1L-SiP_2 heterostructures with the HSE functional. **i**, The band alignment of the strained 1L-MoS_2/1L-SiP_2 heterostructures with the PBE functional. The vacuum level is set as zero.

16. The relaxation of the heterostructure models for the DFT calculations

Aiming to simulate the stacking configurations (AA, AB, and BA) in the moiré patterns of case-I and case-II, four structures are built and labeled as case-I-ABBA (Supplementary Fig. 25d), case-I-AA (Supplementary Fig. 25f), case-II-AABA (Supplementary Fig. 25e), and case-II-AB (Supplementary Fig. 25g). As shown in Supplementary Fig. 25a and b, the 14 × 1 MoS_2 and 13 × 1 SiP_2 are used for the construction of the heterostructure models, in which the lattice constants a and b are 10.69 Å and 45.5 Å, respectively. Specifically, in case-I-ABBA, the lattice model contains AB and BA stackings that exist in the moiré pattern of case-I. In case-I-AA, the lattice model contains only AA stackings. Correspondingly, in case-II-AABA, the lattice model contains AA and BA stackings that exist in the moiré pattern of case-II and in case-II-AB the lattice model contains only AB stackings. For case-I-ABBA and case-II-AABA models, all atoms in the model are allowed to relax. While for case-I-AA and case-II-AB, the initial stacking configurations (I-AA/II-AB) will be destroyed once all the atoms are fully relaxed because the AA and AB stacking are meta-stable structures. Thus, to guarantee these stacking configurations unchanged, three middle atoms in this model are not allowed to move; while other atoms are fully relaxed.

After the relaxation, evident corrugations of the MoS_2 layer can be observed while the structural changes of the SiP_2 layer are negligible (Supplementary Fig. 25d–g). Such a result directly shows the significant influence of the introduced moiré potentials on the 1L-MoS_2/1L-SiP_2 heterointerfaces. As shown in Supplementary Fig. 25, the interlayer distance (d) is used to quantitatively describe the structural corrugations of the MoS_2 layers after the relaxation. Taking the case-I-ABBA structure as an example, the average z coordinate value of the top P layer (pointed by the blue arrow which is nearest to the MoS_2 layer) can be used as the benchmark because of the negligible structural corrugations of the SiP_2 layer. The interlayer distance (d) can be defined as the difference between z coordinates of top P layers in SiP_2 and down S atoms in 1L-MoS_2 (indicated by the red arrow). The values of the interlayer distance (d) for different stacking configurations are listed in Supplementary Table 7.

Supplementary Table 7. The values of the interlayer distance of various stacking configurations

	I-AA	I-AB	I-BA	II-AA	II-AB	II-BA
d (Å)	3.339	3.054	2.614	2.797	3.306	2.945

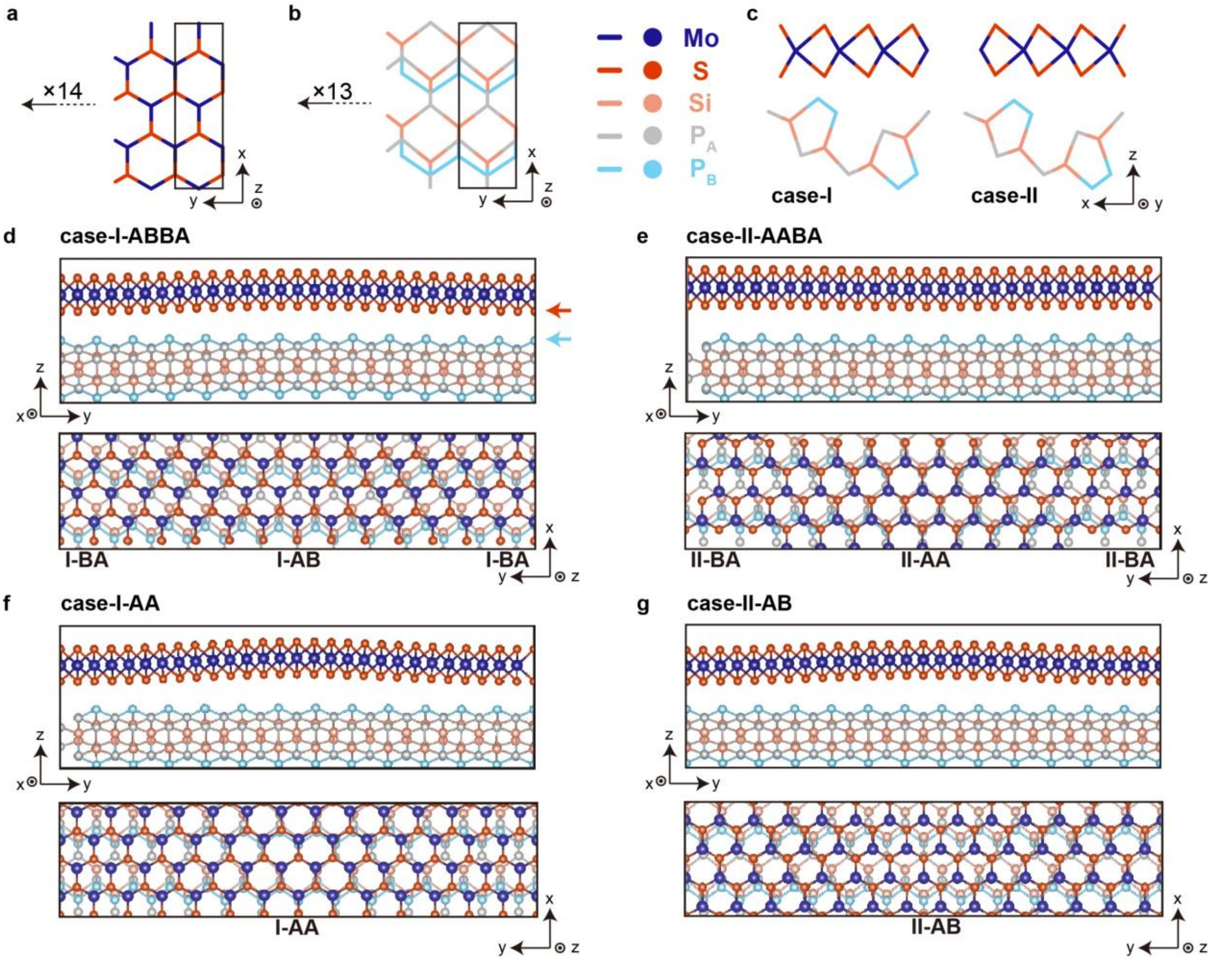

Supplementary Figure 25. Illustrations of the slab models for the DFT calculations. a, The top view of the strained 14 × 1 1L-MoS_2. **b**, The top view of the strained 13 × 1 1L-SiP_2. **c**, The side views of the corresponding slab models. **d**–**g**, The side views and the top views of the four slab models for the DFT calculations, where the slab models contain various stacking configurations and are built by the strained 14 × 1 1L-MoS_2 and 13 × 1 1L-SiP_2 in Supplementary Fig. 25a,b.

17. The electronic structures of the heterostructures in the DFT calculations

To analyze the charge density distribution of the conduction band edge for the 1L-MoS_2 in the heterostructures, we performed DFT calculations with the PBE functional for each strained heterostructure model. In the supercell of heterostructure, the k point of conduction band edge for the strained 1L-MoS_2 is folded to the Γ point in the reduced BZ. Supplementary Fig. 26 shows the charge density distribution of the conduction band edge in real space. One can see that charge density is not distributed uniformly in the heterostructure model and the variation in the charge density distribution in real space results from the structural corrugations of the MoS_2 layer in the heterostructure.

Furthermore, we calculate the plane-averaged charge densities along the *y*/*z* direction of the slab model, which are defined as:

$$\rho(y)' = \frac{\iint \rho(x,y,z)dxdz}{S_{xz}}, \qquad \rho(z)' = \frac{\iint \rho(x,y,z)dxdy}{S_{xy}} \tag{11}$$

where $\rho(x,y,z)$ is the value of the charge density in the real space and S_{xz} (or S_{xy}) is the area of the plane normal to the y (or z) direction. The calculated plane-averaged charge density distributions along the y direction are shown in Supplementary Fig. 26 and guarantee the mirror symmetry along the x direction that is preserved in the heterostructures. Such observation is consistent with the result from SHG measurement. Note that the plane-averaged charge density distribution along the z direction of case-I-ABBA is shown in Fig. 4e in the main text, where electrons are mainly localized in the MoS_2 layer.

Importantly, the CBM of MoS_2 under moiré potential is mainly localized in the I-BA region for the moiré pattern of case-I, and it is in the II-AA stacking areas for the moiré pattern of case-II. Herein, we defined a quantity ($q = \frac{n_e}{N_e}$) to quantitatively describe the charge distribution of conduction band edge in the moiré patterns, where $n_e = \rho(y)'$ is the value of the plane-averaged charge density for the specified stacking configuration along y direction in the supercell model, and N_e is the summation of the charge density in the whole heterostructure supercell. The values of the q of various stackings are listed in Supplementary Table 8.

Taking the case-I-ABBA as the example, the n_e value with I-AB stacking is marked by the circle in Supplementary Fig. 26. Moreover, the N_e values of the case-I-ABBA and case-I-AA are almost the same, which keeps that the absolute values of q of I-AA, I-AB, and I-BA can be used to represent the extent of the localization of the CBM in the corresponding stacking regions. Then we use this quantity to approximate the charge density distribution of the conduction band edge in the moiré patterns (detailed discussions can be found in the next Section). The same methods are used for case-II-AABA and case-II-AB. And, all values of the q for various stackings are listed in Supplementary Table 8.

Supplementary Table 8. The values of the quantity q of various stacking configurations

	I-AA	I-AB	I-BA	II-AA	II-AB	II-BA
q (a.u.)	0.0013	0.0019	0.0035	0.0035	0.0013	0.0020

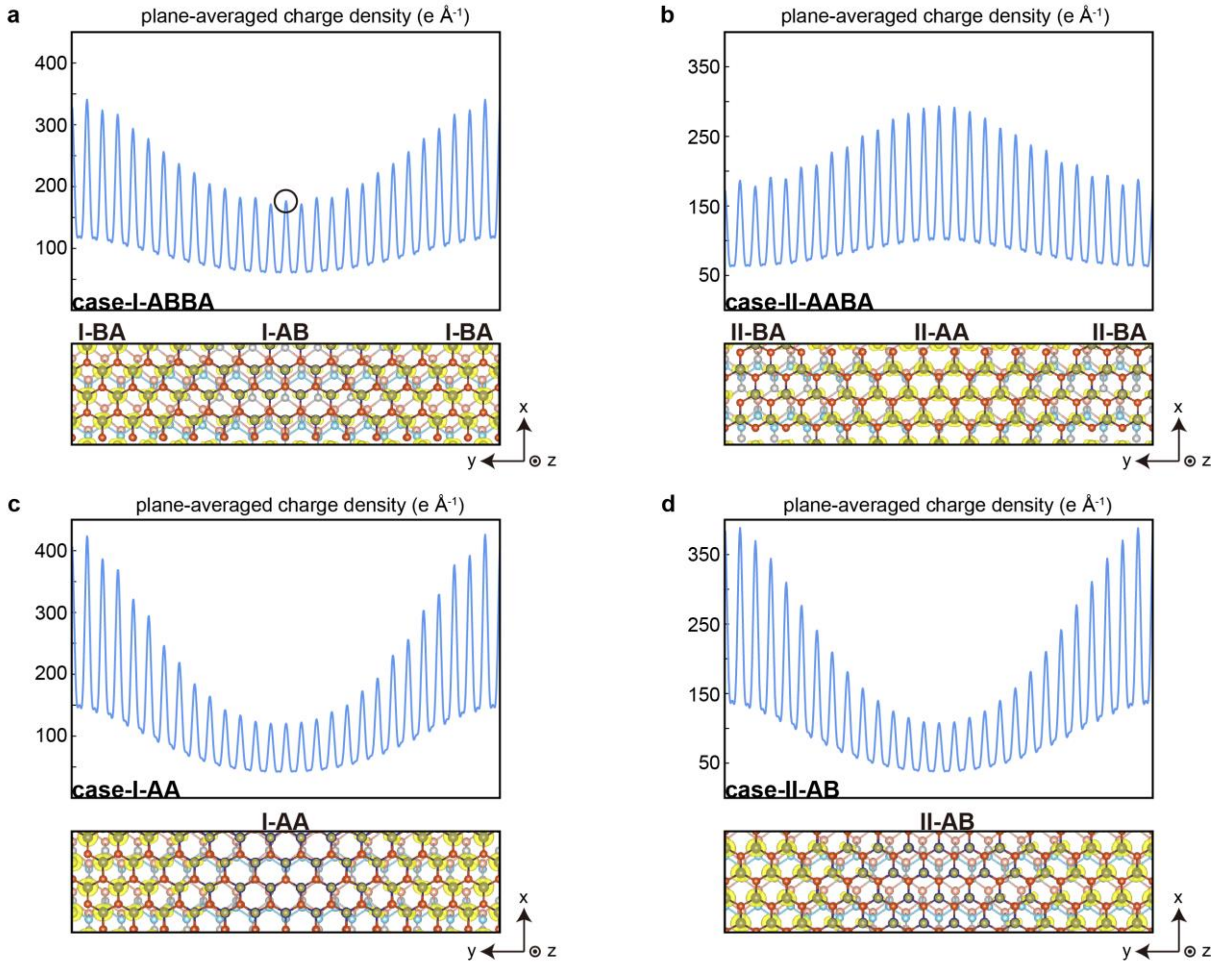

Supplementary Figure 26. Visualizations of the charge density on the conduction band edge in the real space and the corresponding plane-averaged charge density along the *y* direction of the strained slab models. a–d, The visualizations of the charge density distribution in the real space (bottom panel) and the plane-averaged charge density along the *y* direction (top panel) of case-I-ABBA, case-II-AABA, case-I-AA, and case-II-AB, respectively. The iso-surface is set as 0.0002 e $Å^{-3}$.

18. The roles of the interlayer distance and the charge density distribution on the conduction band edge of the moiré superlattices of case-I and case-II

In this section, we show the calculated structural corrugations and charge density distribution of the conduction band edge in the moiré patterns (case-I and case-II). The real space variation of the interlayer distance (d) and the distribution of q are used to depict the moiré potential. For example, for moiré pattern of case-I, we obtain the d and q at the intermediate stacking configurations (I-AA, I-AB, and I-BA stacking configurations) from the DFT calculation with superlattice model (Supplementary Fig. 27a), then do the cubic spline interpolation to get the real space distribution of d and q in the moiré pattern. The colormap (Supplementary Fig. 27b) is plotted to show the structural fluctuations in the moiré superlattice of case-I. The same method is used to other moiré superlattice (Supplementary Fig. 27c, e–f). Note that the values (q) of the stacking configurations (AA, AB, and BA) are enlarged 1,000 times before the interpolation for convenience.

Focusing on the moiré pattern of case-I, it is evident that the stacking areas (I-BA) host the smallest interlayer distances (Supplementary Fig. 27b) and largest electron localization (Supplementary Fig. 27c), indicating the largest moiré potential. One can clearly see that the distribution of the interlayer distance (or the distribution of charge density) shows strong anisotropy with the mirror symmetry parallel to the x direction of the heterostructure (more clearly in Fig. 4 with a wide range of moiré lattices).Thus, the anisotropic moiré potential in the 1L-MoS_2/1L-SiP_2 heterointerface can be described by the structural fluctuation and the real space charge density distribution of the conduction band edge. The states trapped by moiré potential mainly stay at the atomic interface in MoS_2/SiP_2 heterostructure. Such anisotropic moiré potential influences the hopping between the direction along and perpendicular to the mirror plane and eventually corroborates the observed anisotropic conductance. We could use the same methods to the moiré pattern of case-II (Supplementary Fig. 27d–f) and draw the same conclusion.

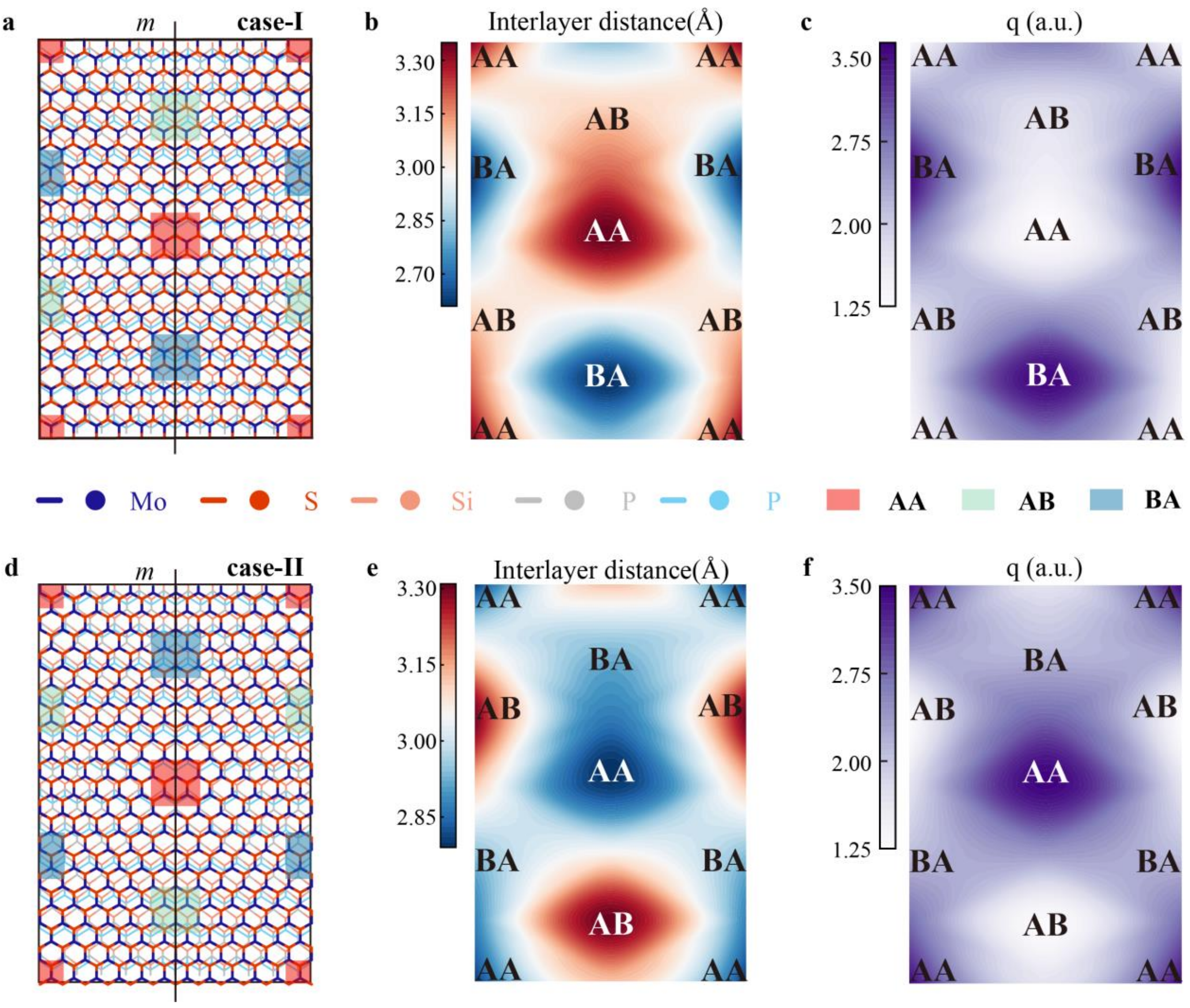

Supplementary Figure 27. The simulations of the structural corrugations and the charge densities at the conduction band edge of the commensurate moiré superlattices of case-I and case-II. a, The top view of the moiré superlattice of case-I. **b**, The simulation of the structural corrugations of the moiré superlattice of case-I. **c**, The charge density at the conduction band edge of the moiré superlattice of case-I. **d**, The top view of the moiré superlattice of case-II. **e**, The simulation of the structural corrugations of the moiré superlattice of case-II. **f**, The charge density at the conduction band edge of the moiré superlattice of case-II.

Supplementary References:

1. Kittel, C. & McEuen, P. *Introduction to solid state physics*. (John Wiley & Sons, 2018).
2. Radisavljevic, B. & Kis, A. Mobility engineering and a metal-insulator transition in monolayer MoS_2. *Nat. Mater.* **12**, 815–820 (2013).
3. Cui, X. *et al.* Multi-terminal transport measurements of MoS_2 using a van der Waals heterostructure device platform. *Nat. Nanotechnol.* **10**, 534–540 (2015).
4. Dean, C. R. *et al.* Boron nitride substrates for high-quality graphene electronics. *Nat. Nanotechnol.* **5**, 722–726 (2010).
5. Yang, W. *et al.* The integration of sub-10 nm gate oxide on MoS_2 with ultra low leakage and enhanced mobility. *Sci Rep-Uk* **5**, 11921 (2015).
6. Liu, K. *et al.* A wafer-scale van der Waals dielectric made from an inorganic molecular crystal film. *Nat. Electron.* **4**, 906–913 (2021).
7. Huang, J. K. *et al.* High-κ perovskite membranes as insulators for two-dimensional transistors. *Nature* **605**, 262–267 (2022).
8. Illarionov, Y. Y. *et al.* Ultrathin calcium fluoride insulators for two-dimensional field-effect transistors. *Nat. Electron.* **2**, 230–235 (2019).
9. Han, T. *et al.* Probing the field-effect transistor with monolayer MoS_2 prepared by APCVD. *Nanomaterials* **9**, 1209 (2019).
10. Lee, G.-H. *et al.* Highly stable, dual-gated MoS_2 transistors encapsulated by hexagonal boron nitride with gate-controllable contact, resistance, and threshold voltage. *ACS Nano* **9**, 7019–7026 (2015).
11. Li, T. *et al.* Continuous Mott transition in semiconductor moire superlattices. *Nature* **597**, 350–354 (2021).

12. Ye, J. T. *et al.* Superconducting dome in a gate-tuned band insulator. *Science* **338**, 1193–1196 (2012).

13. T. Hahn. *International Tables for Crystallography, vol. A: Space-group symmetry* (International Union of Crystallography, 2006).

14. Zhou, L. *et al.* Unconventional excitonic states with phonon sidebands in layered silicon diphosphide. *Nat. Mater.* **21**, 773–778 (2022).

15. Perevalov, T. V. *et al.* in *Defects in High-κ Gate Dielectric Stacks.* (ed Evgeni Gusev) 423–434 (Springer Netherlands).

16. Wilk, G. D., Wallace, R. M. & Anthony, J. M. High-*κ* gate dielectrics: Current status and materials properties considerations. *J. Appl. Phys.* **89**, 5243–5275 (2001).

17. Robertson, J. High dielectric constant oxides. *Eur. Phys. J. Appl. Phys.* **28**, 265–291 (2004).

18. Choi, J. H., Mao, Y. & Chang, J. P. Development of hafnium based high-*κ* materials—A review. *Mater. Sci. Eng. R* **72**, 97–136 (2011).

19. Jain, A. *et al.* Commentary: The Materials Project: A materials genome approach to accelerating materials innovation. *APL Mater.* **1**, 011002 (2013).

20. Petousis, I. *et al.* High-throughput screening of inorganic compounds for the discovery of novel dielectric and optical materials. *Scientific Data* **4**, 160134 (2017).

21. Munro, J. M., Latimer, K., Horton, M. K., Dwaraknath, S. & Persson, K. A. An improved symmetry-based approach to reciprocal space path selection in band structure calculations. *npj Comput. Mater.* **6**, 112 (2020).

22. Perevalov, T. V. *et al.* Electronic structure of α-Al_2O_3: *Ab* initio simulations and comparison with experiment. *JETP Lett.* **85**, 165–168 (2007).

23. Medvedeva, J. E., Teasley, E. N. & Hoffman, M. D. Electronic band structure and carrier

effective mass in calcium aluminates. *Phys. Rev. B* **76**, 155107 (2007).

24. Xu, Y. N. & Ching, W. Y. Self-consistent band structures, charge distributions, and optical-absorption spectra in MgO, α-Al_2O_3, and $MgAl_2O_4$. *Phys. Rev. B* **43**, 4461–4472 (1991).

25. Clima, S. *et al.* Determination of ultimate leakage through rutile TiO_2 and tetragonal ZrO_2 from *ab* initio complex band calculations. *IEEE Electron Device Lett.* **34**, 402–404 (2013).

26. Subramanian, M. A., Shannon, R. D., Chai, B. H. T., Abraham, M. M. & Wintersgill, M. C. Dielectric constants of BeO, MgO, and CaO using the two-terminal method. *Phys. Chem. Minerals* **16**, 741–746 (1989).

27. Li, T. *et al.* A native oxide high-*κ* gate dielectric for two-dimensional electronics. *Nat. Electron.* **3**, 473–478 (2020).

28. Ferreira, F., Chaves, A. J., Peres, N. M. R. & Ribeiro, R. M. Excitons in hexagonal boron nitride single-layer: a new platform for polaritonics in the ultraviolet. *J. Opt. Soc. Am. B* **36**, 674–683 (2019).

29. Watanabe, K., Taniguchi, T. & Kanda, H. Direct-bandgap properties and evidence for ultraviolet lasing of hexagonal boron nitride single crystal. *Nat. Mater.* **3**, 404–409 (2004).

30. Young, A. F. *et al.* Electronic compressibility of layer-polarized bilayer graphene. *Phys. Rev. B* **85**, 235458 (2012).

31. Duan, S. *et al*. Berry curvature dipole generation and helicity-to-spin conversion at symmetry-mismatched heterointerfaces. *Nat. Nanotechnol.* **18**, 867–874 (2023).

32. Zhang, Y. *et al.* Controllable magnetic proximity effect and charge transfer in 2D semiconductor and double-layered perovskite manganese oxide van der Waals heterostructure. *Adv. Mater.* **32**, e2003501 (2020).

33. Mak, K. F., He, K., Shan, J. & Heinz, T. F. Control of valley polarization in monolayer

MoS_2 by optical helicity. *Nat. Nanotechnol.* **7**, 494–498 (2012).

34. Bai, Y. *et al.* Excitons in strain-induced one-dimensional moire potentials at transition metal dichalcogenide heterojunctions. *Nat. Mater.* **19**, 1068–1073 (2020).
35. Liang, J. *et al.* Monitoring local strain vector in atomic-layered $MoSe_2$ by second-harmonic generation. *Nano Lett.* **17**, 7539–7543 (2017).
36. Mennel, L. *et al.* Optical imaging of strain in two-dimensional crystals. *Nat. Commun.* **9**, 516 (2018).
37. Abe, S., Inaoka, T. & Hasegawa, M. Evolution of electron states at a narrow-gap semiconductor surface in an accumulation-layer formation process. *Phys. Rev. B* **66**, 205309 (2002).
38. Kang, J., Tongay, S., Zhou, J., Li, J. & Wu, J. Band offsets and heterostructures of two-dimensional semiconductors. *Appl. Phys. Lett.* **102**, 012111 (2013).
39. Kim, H.-g. & Choi, H. J. Thickness dependence of work function, ionization energy, and electron affinity of Mo and W dichalcogenides from DFT and GW calculations. *Phys. Rev. B* **103**, 085404 (2021).
40. Mak, K. F., Lee, C., Hone, J., Shan, J. & Heinz, T. F. Atomically thin MoS_2: A new direct-gap semiconductor. *Phys. Rev. Lett.* **105**, 136805 (2010).
41. Matta, S. K., Zhang, C., Jiao, Y., O'Mullane, A. & Du, A. Versatile two-dimensional silicon diphosphide (SiP_2) for photocatalytic water splitting. *Nanoscale* **10**, 6369-6374 (2018).
